# Modulation of Uptake and Reactivity of Nitrogen Dioxide in Metal‐Organic Framework Materials

**DOI:** 10.1002/anie.202302602

**Published:** 2023-06-02

**Authors:** Zi Wang, Alena M. Sheveleva, Daniel Lee, Yinlin Chen, Dinu Iuga, W. Trent Franks, Yujie Ma, Jiangnan Li, Lei Li, Yongqiang Cheng, Luke L. Daemen, Sarah J. Days, Anibal J. Ramirez‐Cuesta, Bing Han, Alexander S. Eggeman, Eric J. L. McInnes, Floriana Tuna, Sihai Yang, Martin Schröder

**Affiliations:** ^1^ Department of Chemistry University of Manchester Manchester M13 9PL UK; ^2^ Photon Science Institute University of Manchester Manchester M13 9PL UK; ^3^ Department of Chemical Engineering and Analytical Science University of Manchester Manchester M13 9PL UK; ^4^ Department of Physics University of Warwick Coventry CV4 7AL UK; ^5^ Neutron Scattering Division Oak Ridge National Laboratory Oak Ridge TN 37831 USA; ^6^ Diamond Light Source Harwell Science Campus Oxfordshire OX11 0DE UK; ^7^ Department of Materials University of Manchester Manchester M13 9PL UK

**Keywords:** Conversion, Metal–Organic Framework, Nitrogen Dioxide, Reactivity Modulation, Spectroscopy

## Abstract

We report the modulation of reactivity of nitrogen dioxide (NO_2_) in a charged metal–organic framework (MOF) material, MFM‐305‐CH_3_ in which unbound N‐centres are methylated and the cationic charge counter‐balanced by Cl^−^ ions in the pores. Uptake of NO_2_ into MFM‐305‐CH_3_ leads to reaction between NO_2_ and Cl^−^ to give nitrosyl chloride (NOCl) and NO_3_^−^ anions. A high dynamic uptake of 6.58 mmol g^−1^ at 298 K is observed for MFM‐305‐CH_3_ as measured using a flow of 500 ppm NO_2_ in He. In contrast, the analogous neutral material, MFM‐305, shows a much lower uptake of 2.38 mmol g^−1^. The binding domains and reactivity of adsorbed NO_2_ molecules within MFM‐305‐CH_3_ and MFM‐305 have been probed using in situ synchrotron X‐ray diffraction, inelastic neutron scattering and by electron paramagnetic resonance, high‐field solid‐state nuclear magnetic resonance and UV/Vis spectroscopies. The design of charged porous sorbents provides a new platform to control the reactivity of corrosive air pollutants.

## Graphical abstract

The reactivity of NO_2_ is modulated in a charged metal–organic framework, MFM‐305‐CH_3_, which leads to reaction between NO_2_ and framework Cl^−^ to give nitrosyl chloride (NOCl) and NO_3_^−^ anions. A higher NO_2_ dynamic uptake (6.58 mmol g^−1^) is achieved, compared with the analogous neutral material, MFM‐305 (2.38 mmol g^−1^).

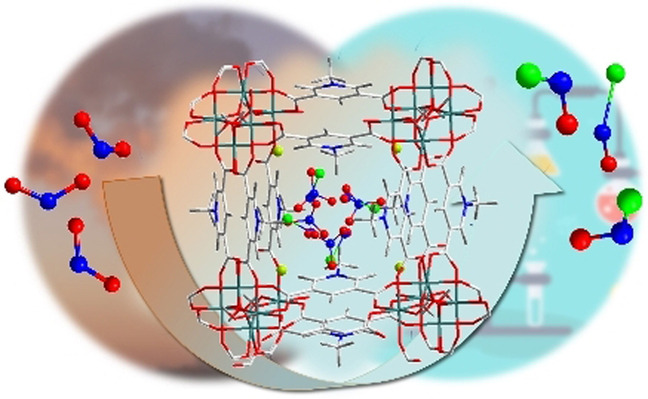

## Introduction

Nitrogen oxide (NO_2_) is an important air pollutant that causes serious environmental and health problems.[^1^, ^2^, ^3^, ^4^] It can irritate the respiratory tract, thus increasing the risk of respiratory infections and asthma, particularly for children and people with respiratory problems. Additionally, constant exposure to NO_2_ has been linked to an increased risk of heart disease, stroke, and other cardiovascular problems.^[5]^ Although many countries have legislated to restrict the emission of NO_*x*_ by vehicles, the concentration of NO_2_ in the atmosphere continues to increase.^[5]^ Selective catalytic reductions (SCR) over precious‐metal catalysts with toxic reductants (e.g., NH_3_) at elevated temperatures are widely applied to convert NO_2_ to N_2_.[^6^, ^7^, ^8^] Alternatively, porous sorbents offer a promising pathways to capture NO_2_ under ambient conditions. Conventional porous materials based upon zeolites and activated carbons generally suffer from low uptake and/or severe structural degradations on adsorption of NO_2_ owing to its highly reactive and corrosive properties.[^9^, ^10^, ^11^, ^12^, ^13^]

Metal‐organic framework materials show exceptional performance in gas adsorption and storage due to their high porosity and stability.[^14^, ^15^, ^16^] Recently, adsorption of NO_2_ in MOFs and MOF‐based composites has been reported.[^17^, ^18^, ^19^, ^20^, ^21^] The first example of reversible adsorption of NO_2_ in MOFs was achieved by MFM‐300(Al), which shows a high uptake of 14.1 mmol g^−1^ at 298 K and 1 bar.^[22]^ UiO‐66‐NH_2_ shows a high adsorption of NO_2_ (up to 31.2 mmol g^−1^) owing to the irreversible chemical reaction with NO_2_ to form diazonium species on the aromatic ring of the organic linker, and additionally by reaction with water molecules under humid conditions.^[23]^ However, the molecular details of the reactivity of adsorbed NO_2_ molecules within the pore of MOFs have been poorly explored, thus restricting the design of new efficient sorbent materials to mitigate the emission of NO_2_.

**Figure 1:**
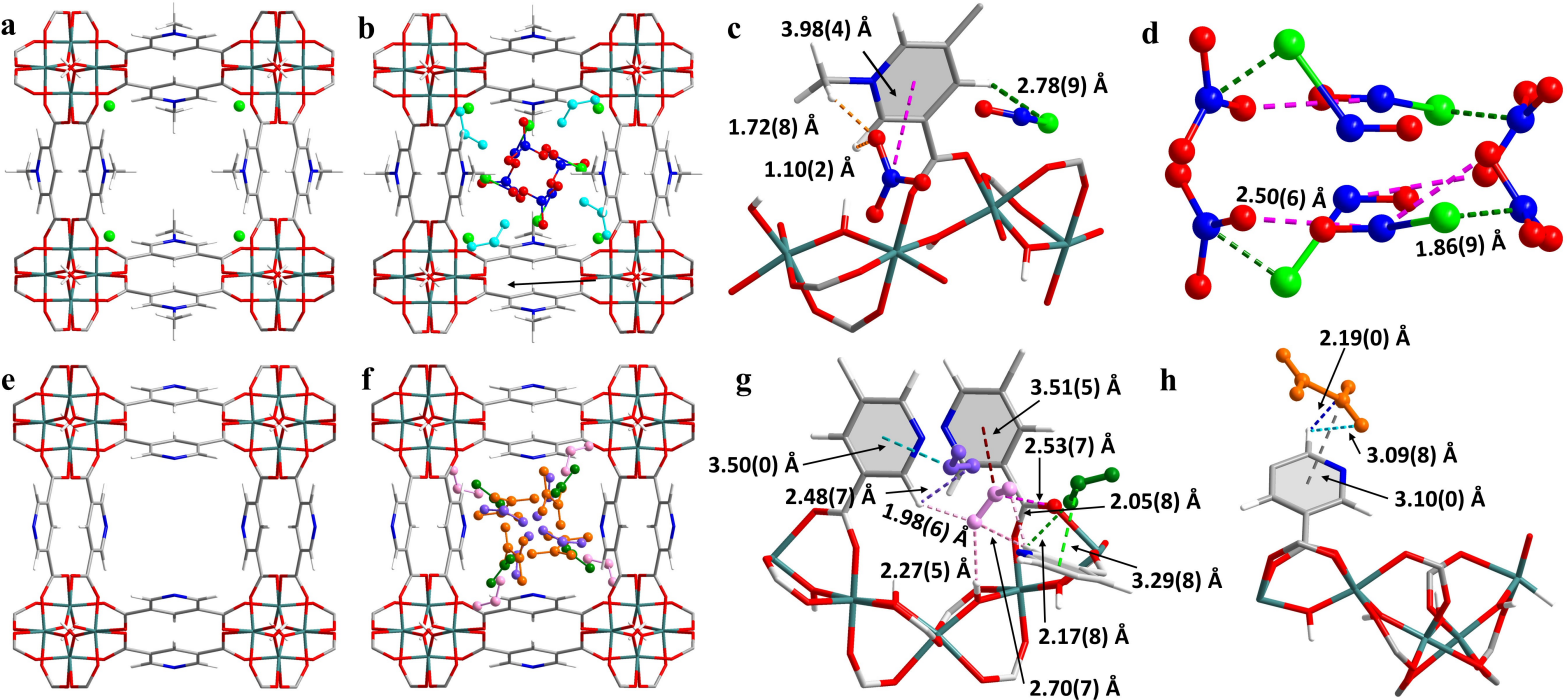
Views of structures of [Al(OH)(C_8_H_6_NO_4_)Cl] (MFM‐305‐CH_3_), [Al(OH)(C_7_H_3_NO_4_)] (MFM‐305), [Al(OH)(C_8_H_6_NO_4_)Cl_0.78_⋅(NOCl)_0.22_⋅(NO_3_)_0.22_⋅(NO_2_)_0.5_] (NO_2_‐loaded MFM‐305‐CH_3_) and [Al(OH)(C_7_H_3_NO_4_)⋅(NO_2_)_1.7_⋅(NO_2_)_1.0_⋅(NO_2_)_0.45_⋅(N_2_O_4_)_0.175_] (NO_2_‐loaded MFM‐305). All structures were derived from Rietveld refinements of in situ synchrotron X‐ray powder diffraction data collected at 298 K (C: grey; N: blue; O: red Al: sea green; H: white; Cl: lime). a) View of MFM‐305‐CH_3_; b) packing of adsorbed NOCl and NO_3_^−^ in MFM‐305‐CH_3_ (viewed down *c* axis) (cyan: NO_2_ left after reaction); c) view of binding site of NOCl and NO_3_^−^ in MFM‐305‐CH_3_; d) packing of NOCl‐NO_3_^−^ in the pore of MFM‐305‐CH_3_; e) view of MFM‐305; f) packing of adsorbed NO_2_ and N_2_O_4_ molecules in MFM‐305 (viewed down the *c* axis) (orange: N_2_O_4_; pink: NO_2_ at site I; green: NO_2_ at site II; purple: NO_2_ at site III); g) view of binding sites of monomer NO_2_ in MFM‐305; h) view of binding site of N_2_O_4_ in MFM‐305.

Here, we report the modulation of reactivity of NO_2_ in a pair of closely related MOFs, MFM‐305‐CH_3_ and MFM‐305. MFM‐305‐CH_3_, [Al(OH)(L)]Cl, (H_2_L)Cl=3,5‐dicarboxy‐1‐methylpyridinium chloride],^[24]^ has an unusual charged structure incorporating cationic (methylpyridinium) and anionic (Cl^−^) components to give a zwitterionic‐type framework (Figure 1a). MFM‐305‐CH_3_ has an open framework structure comprised of corner‐sharing chains of [AlO_4_(OH)_2_]_∞_ bridged by dicarboxylate ligands. The Al^III^ centres show octahedral coordination defined by four carboxylate oxygen atoms from L^2−^ ligands and two oxygen atoms from two μ_2_‐hydroxyl groups. MFM‐305‐CH_3_ can undergo post‐synthetic modification via heating at 180 °C involving the 1‐methylpyridiniumdicarboxylate ligand undergoing in situ demethylation. This is coupled to the loss of Cl^−^ anion as CH_3_Cl to give the pyridyl‐based neutral framework in MFM‐305 (Figure 1e), which has the same network topology and metal‐ligand coordination as MFM‐305‐CH_3_.

We sought to monitor the impact of the differences in these materials on their interaction and reactivity with NO_2_. The framework structure in both MFM‐305‐CH_3_ and MFM‐305 shows high stability upon adsorption of NO_2_. However, adsorption of NO_2_ into MFM‐305‐CH_3_ leads to reaction with Cl^−^ in the pore to give NOCl and NO_3_^−^, confirmed by in situ infrared (IR), high field solid‐state nuclear magnetic resonance (ssNMR) and UV/Vis spectroscopy. The binding domains within the frameworks have been revealed using synchrotron X‐ray powder diffraction. A high dynamic uptake of NO_2_ of 6.58 mmol g^−1^ at 298 K is observed for MFM‐305‐CH_3_ as measured using a flow of 500 ppm NO_2_ in He. NOCl can be extracted with CHCl_3_ and recovered for use as a reagent for organic synthesis, including N‐nitrosation of secondary amines, the structural elucidation of terpenes and the production of aromatic diazonium salts from anilines.^[25]^ Therefore, this work develops a new method for converting NO_2_ to useful chemicals, thus converting and utilising a key air pollutant in subsequent chemical processes. MFM‐305‐CH_3_ can be regenerated via ion‐exchange and extraction of NO_3_^−^ with Cl^−^ ions. In contrast, the adsorbed NO_2_ molecules in MFM‐305 do not react with the framework and an uptake of 2.38 mmol g^−1^ at 298 K is observed. The host–guest binding interactions in both MOFs have been further studied by in situ electron paramagnetic resonance (EPR) spectroscopy and inelastic neutron scattering (INS) coupled with density functional theory (DFT) modelling.

## Results and Discussion

**Figure 2:**
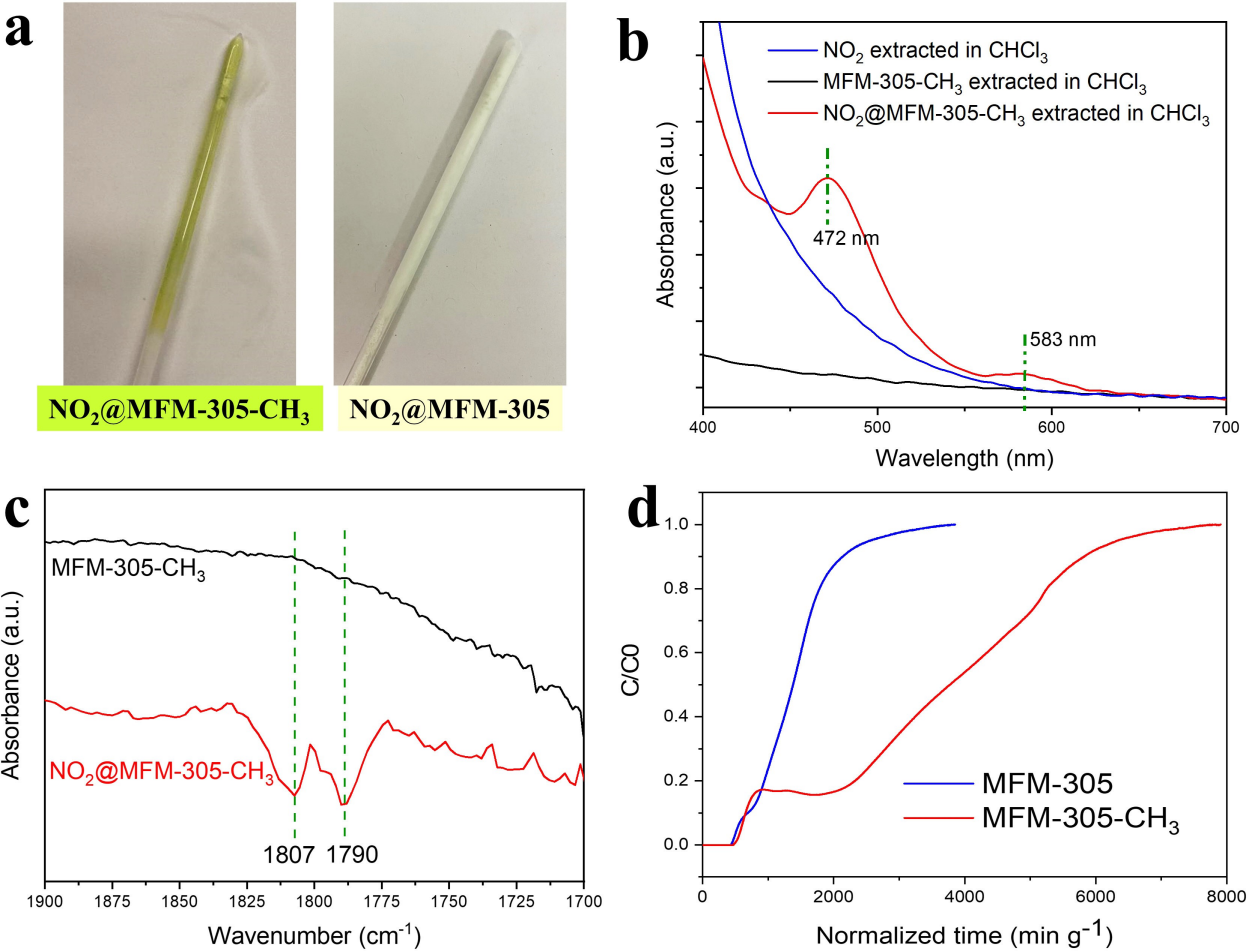
Characterisation of adsorption of NO_2_ in MFM‐305‐CH_3_ and MFM‐305. a) Photographs of NO_2_‐loaded MFM‐305‐CH_3_ and MFM‐305 at 77 K; b) UV/Vis spectra of CHCl_3_ solution extracted from NO_2_@MFM‐305‐CH_3_ (red), bare MFM‐305‐CH_3_ (black), gas NO_2_ (blue); c) in situ DRIFTs of activated (black) and NO_2_‐loaded MFM‐305‐CH_3_ (red); d) dynamic breakthrough plots of MFM‐305‐CH_3_ and MFM‐305 using a gas stream of 500 ppm NO_2_ in dry He at 298 K. The normalised concentration represents C/C_0_ where C=concentration of NO_2_ at outlet, C_0_=concentration of NO_2_ at inlet. C/C_0_=1 indicates complete breakthrough.

Upon introducing NO_2_ into MFM‐305‐CH_3_, a yellow‐green liquid was observed on cooling the sample to 77 K (Figure 2a). This behaviour is, however, not observed for NO_2_‐loaded MFM‐305. Extracting NO_2_‐loaded MFM‐305‐CH_3_ with CHCl_3_ gives a dark orange solution, the UV/Vis spectrum of which shows two bands at 472 and 583 nm (Figure 2b) characteristic for NOCl.^[25]^ These bands are absent in the spectra of NO_2_ dissolved in CHCl_3_, and are not observed on extraction of pristine MFM‐305‐CH_3_ with CHCl_3_, In addition, CHCl_3_ is unreactive towards N−Cl species, including NOCl, and these observations suggest a chemical reaction of NO_2_ taking place within MFM‐305‐CH_3_ involving the Cl^−^ anions.

In situ diffuse reflectance infrared Fourier transform spectroscopy (DRIFTs) was applied to probe the formation of NOCl. Upon adsorption of NO_2_ in activated MFM‐305‐CH_3_, two new peaks appeared at 1790 and 1807 cm^−1^, assigned as the characteristic *ν*(N=O) stretching in NOCl interacting with a solid surface, the doublet feature being due to the rotational barrier of the N=O bond upon confinement in the pore (Figure 2c).[^26^, ^27^, ^28^] Attempts to identify IR bands for NO_3_^−^ near 1360 and 840 cm^−1^ ^[27]^ were hampered due to overlapping peaks from the framework. The reaction between NO_2_ and Cl^−^ has been studied by mixing NO_2_ with NaCl as a non‐porous material.^[27]^ The reaction at room temperature reaches the equilibrium in 1 h controlled by the limited adsorption of NO_2_ onto the external surface of NaCl. In contrast, confined NO_2_ molecules within MFM‐305‐CH_3_ react with Cl^−^ immediately (even at 77 K) due to the presence of strong host–guest interactions. Elemental analysis by inductively coupled plasma atomic emission spectroscopy (ICP‐AES) was performed on: (i) the as‐synthesised MFM‐305‐CH_3_ [Al(OH)(C_8_H_6_NO_4_)Cl⋅3 H_2_O], (ii) re‐activated MFM‐305‐CH_3_ after adsorption of NO_2_ and removal of NOCl by heating at 100 °C for 10 h under dynamic vacuum [Al(OH)(C_8_H_6_NO_4_)NO_3_], and (iii) regenerated MFM‐305‐CH_3_ [Al(OH)(C_8_H_6_NO_4_)Cl⋅3 H_2_O] by soaking the re‐activated material in NaCl‐MeOH solution for 3 days (Table S2, Figure S3). The analysis shows that ca. 40 % of the Cl^−^ anion content was converted to NOCl and removed via the re‐activation process, and that ca. 95 % of MFM‐305‐CH_3_ can be regenerated by ion exchange in NaCl solution.

The conversion of NO_2_ in MFM‐305‐CH_3_ can also be observed by dynamic breakthrough experiments using a gas stream containing 500 ppm of NO_2_ (diluted in He) at 298 K. From 0 to 840 min g^−1^ (quoted as time for breakthrough of NO_2_ per gram of porous material, Figure 2d) breakthrough proceeds as normal with NO_2_ being expelled from the column. However, from 840 to 2000 min g^−1^, the detected concentration of NO_2_ at the outlet remains unchanged at a normalised concentration of C/C_0_=0.2 consistent with NO_2_ molecules introduced to the column being immobilised within the material by reaction with Cl^−^. Thus, no additional NO_2_ was detected at the outlet during this period up to 2000 min g^−1^. Once reaction within the pores is complete, the NO_2_ can breakthrough as normal as observed after 2000 min g^−1^ (Figure 2d). Thus, MFM‐305‐CH_3_ captures NO_2_ by physisorption and then chemisorption via reaction with Cl^−^ anion leading to an observed increase in its dynamic capacity for NO_2_ to 6.58 mmol g^−1^ compared with MFM‐305 (2.38 mmol g^−1^), despite the latter material having a notably larger surface area (256 and 779 m^2^ g^−1^, respectively). Thus, the breakthrough plot of dynamic adsorption of NO_2_ in MFM‐305‐CH_3_ has a distinct profile and demonstrates the positive impact of reactivity of NO_2_ on the adsorption and capture performance of the porous host.[^29^, ^30^]

**Figure 3:**
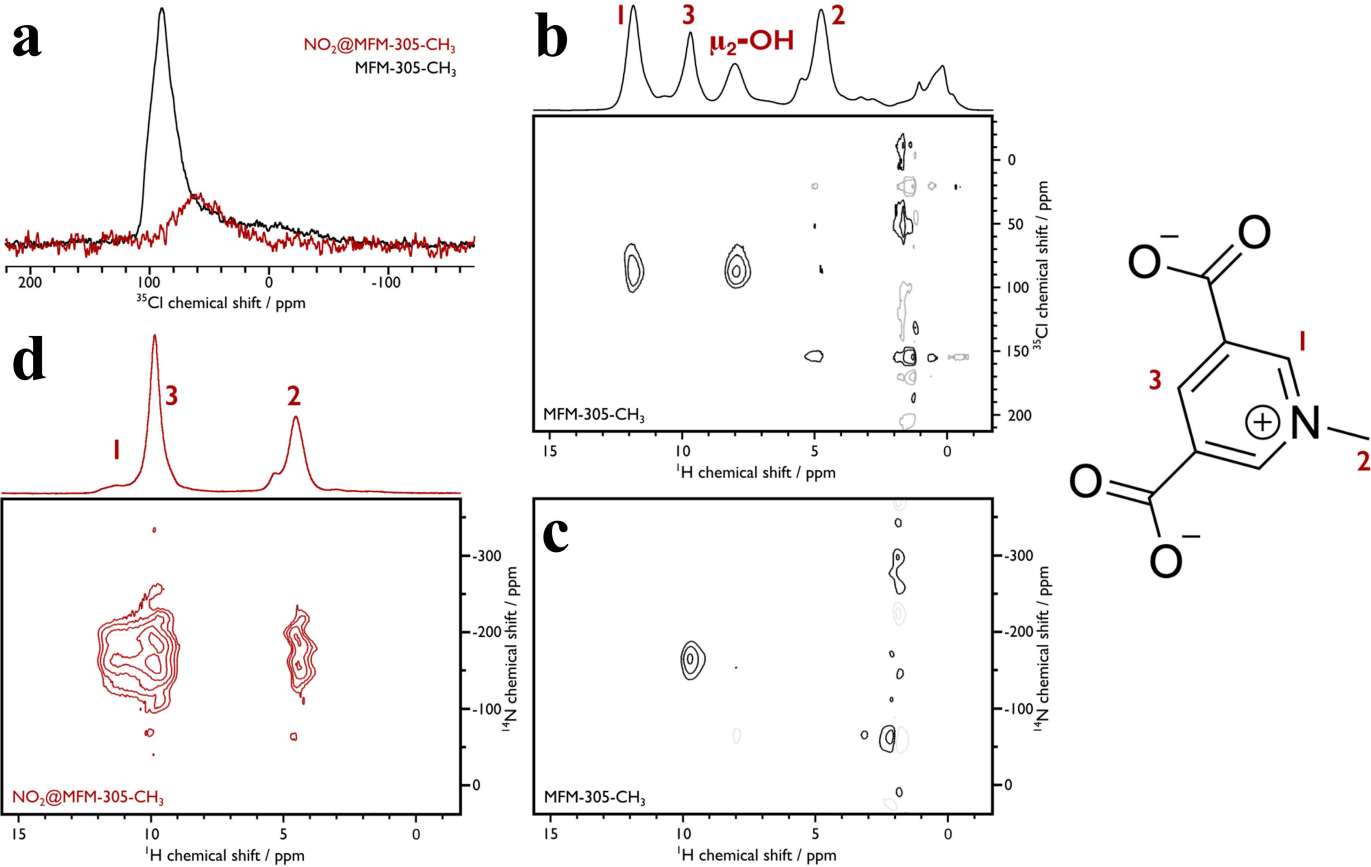
High‐field (23.5 T) MAS NMR spectra. a) ^35^Cl NMR spectra of MFM‐305‐CH_3_ (black) and NO_2_@MFM‐305‐CH_3_ (red); b) 2D ^35^Cl‐^1^H dipolar correlation spectrum of MFM‐305‐CH_3_ with corresponding ^1^H NMR spectrum (top); c) 2D ^14^N‐^1^H dipolar correlation spectrum of MFM‐305‐CH_3_; d) 2D ^14^N‐^1^H dipolar correlation spectrum of NO_2_@MFM‐305‐CH_3_ with corresponding ^1^H NMR spectrum (top). All spectra were recorded at ambient temperature using a MAS frequency of 60 kHz. The 1‐methylpyridiniumdicarboxylate ligand is shown (right) with proton environments numbered for the assignments given for the ^1^H spectra.

To monitor the reaction process, ssNMR spectroscopy was employed at high (23.5 T) and moderate (9.4 T) magnetic fields to investigate the structural changes in MFM‐305‐CH_3_ before and after loading with NO_2_. MFM‐305‐CH_3_ has a ^35^Cl NMR peak at δ{^35^Cl} ≈90 ppm, which decreases markedly in intensity on adsorption of NO_2_ (Figure 3a). To locate the Cl^−^ ions in the structure of MFM‐300‐CH_3_, a two‐dimensional (2D) ^35^Cl‐^1^H through‐space (dipolar) correlation experiment was performed (Figure 3b). Two correlations are observed, with protons at δ{^1^H} ≈8 and 12 ppm, which correspond to μ_2_‐OH and the H1 aromatic proton, respectively. This observation implies the presence hydrogen bonding between these protons and the Cl^−^ anion (Figure S12), consistent with the Cl^−^ position as determined by synchrotron X‐ray powder diffraction (see below), where Cl^−^ ions are found to hydrogen‐bond to these two sites.^[24]^ ^1^H NMR assignments in Figure 3b were determined from 2D ^1^H‐^1^H, ^1^H‐^13^C and ^1^H‐^27^Al dipolar correlation spectra (Figures S9a, b and c, respectively). No correlations were be observed in the corresponding 2D ^35^Cl‐^1^H spectra of NO_2_@MFM‐305‐CH_3_ owing to the reduced ^35^Cl signal (Figure S10).

Upon adsorption of NO_2_, the ^14^N NMR chemical shift of the methylpyridinium nitrogen changes slightly in the 2D ^14^N‐^1^H dipolar correlation spectrum, and another peak is observed upfield (*cf*. Figure 3c and Figure 3d). Neither peak is observed at the frequency expected for confined NO_3_^−^,^[31]^ which indicates that the adsorbed species undergoes rapid motion, which averages the guest‐host ^14^N‐^1^H dipolar coupling. Nevertheless, the (at least) two ^14^N peaks observed for NO_2_@MFM‐305‐CH_3_ indicate that the NO_2_ induces structural changes at the methylpyridinium N‐centre. Moreover, correlations are observed between these two ^14^N resonances and protons at δ{^1^H}=4.6 ppm from the methyl group unlike for pristine MFM‐305‐CH_3_ that does not exhibit ^14^N‐^1^H correlations with the methyl group. This indicates that the methyl rotation has slowed, likely from hydrogen bonding with NO_3_^−^ (see below), enabling a more efficient dipolar‐based transfer of polarisation between methyl ^1^H and framework ^14^N centers. Although the NO_3_^−^ is not observed directly by ^14^N‐^1^H NMR spectroscopy, the presence of hydrogen bonded nitric acid is implied through the differences in ^1^H spectra between samples and the extremely high‐shifted ^1^H NMR peak that appears (at δ{^1^H} ≈18 ppm) upon adsorption of NO_2_ (Figure S11b) that can only come from acidic protons involved in hydrogen bonding.^[32]^ The framework structure of the MOF undergoes only a slight modification upon NO_2_ adsorption and corresponding loss of Cl^−^, with the ligand carbon environments and the octahedral geometry of the aluminium sites undergoing small perturbations, as observed via ^13^C and ^27^Al NMR spectroscopy (Figure S9b and Figure S9c, respectively).

A control experiment was designed to monitor how the disproportionation reaction of NO_2_ takes place via high‐resolution synchrotron X‐ray powder diffraction (SPXRD) using a sample of MFM‐305‐CH_3_ loaded with a small amount of NO_2_. Rietveld refinement of the SPXRD data gave the formula [Al(OH)(C_8_H_6_NO_4_)Cl_0.78_⋅(NOCl)_0.22_⋅(NO_3_)_0.22_⋅(NO_2_)_0.5_]. In the refined model (Figure 1b), 22 % of Cl^−^ anions are converted to NOCl, with formation of the same amount of NO_3_^−^ balancing the charge. The remaining 78 % of Cl^−^ ions remain at their initial positions (i.e., in the corner of the channel), and the newly‐produced guest NOCl is anchored in the pores through the hydrogen bonding to the aromatic proton [Cl^NOCl^⋅⋅⋅H3^aromatic^=2.78(9) Å]. The NO_3_^−^ ions interact with the methyl groups and aromatic protons at a short distance [O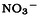 ⋅⋅⋅H2^methyl^=1.72(8) Å and O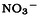 ⋅⋅⋅H1^aromatic^=1.10(2) Å] via hydrogen bonding. In addition, a dipole interaction between NO_3_^−^ and the pyridinium group is observed [O_3_N(δ+)^−^⋅⋅⋅py(δ−)=3.98(4) Å] (Figure 1c). A minor amount of NO_2_ (occupancy of 0.5 NO_2_/Al) was observed near the corners of the pores upon the equilibrium of the reaction, showing strong hydrogen bonding interaction with the aromatic proton [O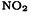 ⋅⋅⋅H1^aromatic^=2.15(9) Å] and the hydroxyl group [O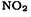 ⋅⋅⋅H^hydroxyl^=2.88(6) Å] (Figure S4). In NO_2_‐loaded MFM‐305 at the same loading, structural analysis gives a model for [Al(OH)(C_7_H_3_NO_4_)⋅(NO_2_)_1.7_⋅(NO_2_)_1.0_⋅(NO_2_)_0.45_⋅(N_2_O_4_)_0.175_] that shows three different NO_2_ binding sites and one N_2_O_4_ binding site (Figure 1f). The NO_2_ molecules at site I (occupancy=1.7 NO_2_/Al) are located in the corner of the pore and exhibit strong hydrogen bonding interaction with the aromatic protons [O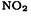 ⋅⋅⋅H1^aromatic^=1.98(6) Å and 2.05(8) Å], and with the hydroxyl group [O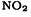 ⋅⋅⋅H^hydroxyl^=2.27(5) Å]. Additional interactions to the carboxylate group [O_2_ N⋅⋅⋅OOC=2.53(7) Å] and the pyridinium ring [O_2_ N⋅⋅⋅py=3.51(5) Å] are also observed. The NO_2_ molecules at site II (occupancy=1.0 NO_2_/Al) are closer to the centre of the pore and interact with the framework via hydrogen bonding [O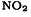 ⋅⋅⋅H1^aromatic^=2.17(8) Å] and are further stabilised by dipole interactions [O_2_ N⋅⋅⋅py=3.29(8) Å]. NO_2_ at site III shows the lowest occupancy (0.45 NO_2_/Al) and is located in the middle of the pore [O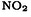 ⋅⋅⋅H1^aromatic^=2.48(7) Å, O_2_ N⋅⋅⋅py=3.50(0) Å] (Figure 1g). Only a small amount of N_2_O_4_ was observed (occupancy=0.35 NO_2_/Al), which also shows hydrogen bonding with aromatic protons [O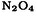 ⋅⋅⋅H1^aromatic^=2.19(0) Å and 3.09(8) Å] and a dipole interaction [O_2_ N⋅⋅⋅py=3.10(0) Å] (Figure 1h). The different binding sites also form various monomer‐to‐monomer, monomer‐to‐dimer and dimer‐to‐dimer dipole interactions that stabilise the adsorbed NO_2_ molecules in the pore (Figure S6).

**Figure 4:**
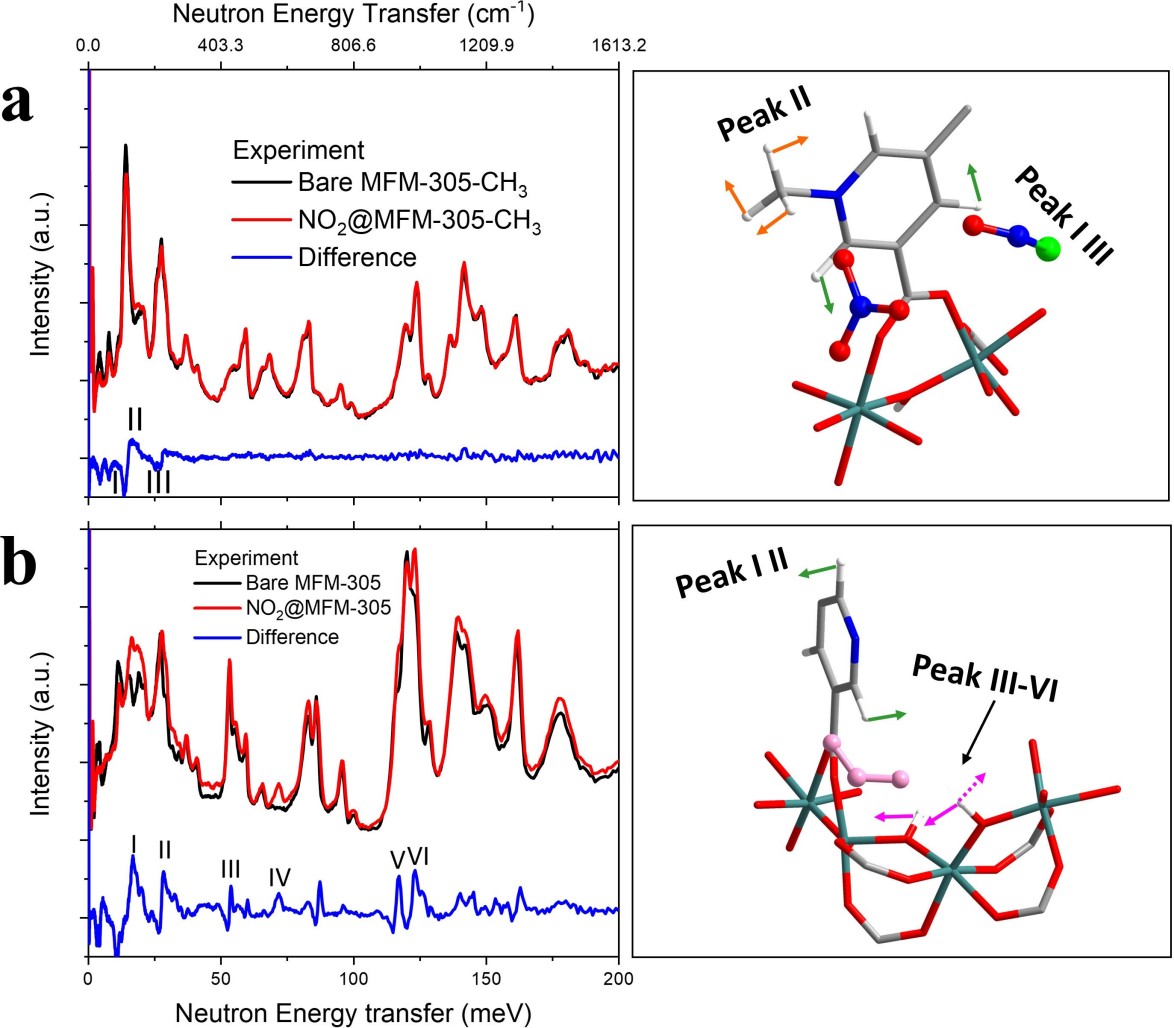
INS spectroscopic data. Comparison of the experimental INS spectra (left) and views of the corresponding structural model (right) for bare and NO_2_‐loaded a) MFM‐305‐CH_3_ and b) MFM‐305.

In situ INS, coupled with DFT calculations, enabled the direct visualisation of the binding dynamics in NO_2_‐loaded MFM‐305‐CH_3_ and MFM‐305 with a focus on the −CH groups involved in supramolecular contacts (Figure 4). Addition of NO_2_ to MFM‐305‐CH_3_ is accompanied by significant changes to peaks at 14 and 26 meV (peaks I and III) consistent with stiffening and deformation of the lattice modes upon binding of NO_2_. Another notable change in intensity is observed at 19 meV (peak II), indicating the hindrance of rotation motion of −CH_3_ groups upon production of NOCl and NO_3_^−^ species in the pore, consistent with the formation of hydrogen bonds as observed in the structural model. Small changes of intensity were also observed in the high energy region (120–200 meV), which correspond to changes of the aromatic −CH groups (twisting/scissoring/wagging modes) (Figure 4a, S13, S15). The INS spectra of NO_2_‐loaded MFM‐305 gave similar changes in intensities at 17 and 28 meV (peaks I–II) assigned to deformational mode of rings. Moreover, significant changes to peaks at 53, 72, 116 and 123 meV (peaks III–VI) indicate the bridging hydroxyl is involved in binding to NO_2_. Changes in C−H modes also can be observed above 120 meV (Figure 4b, S14, S16). Thus, the results of INS are in good agreement with the in situ crystallographic analyses.

**Figure 5:**
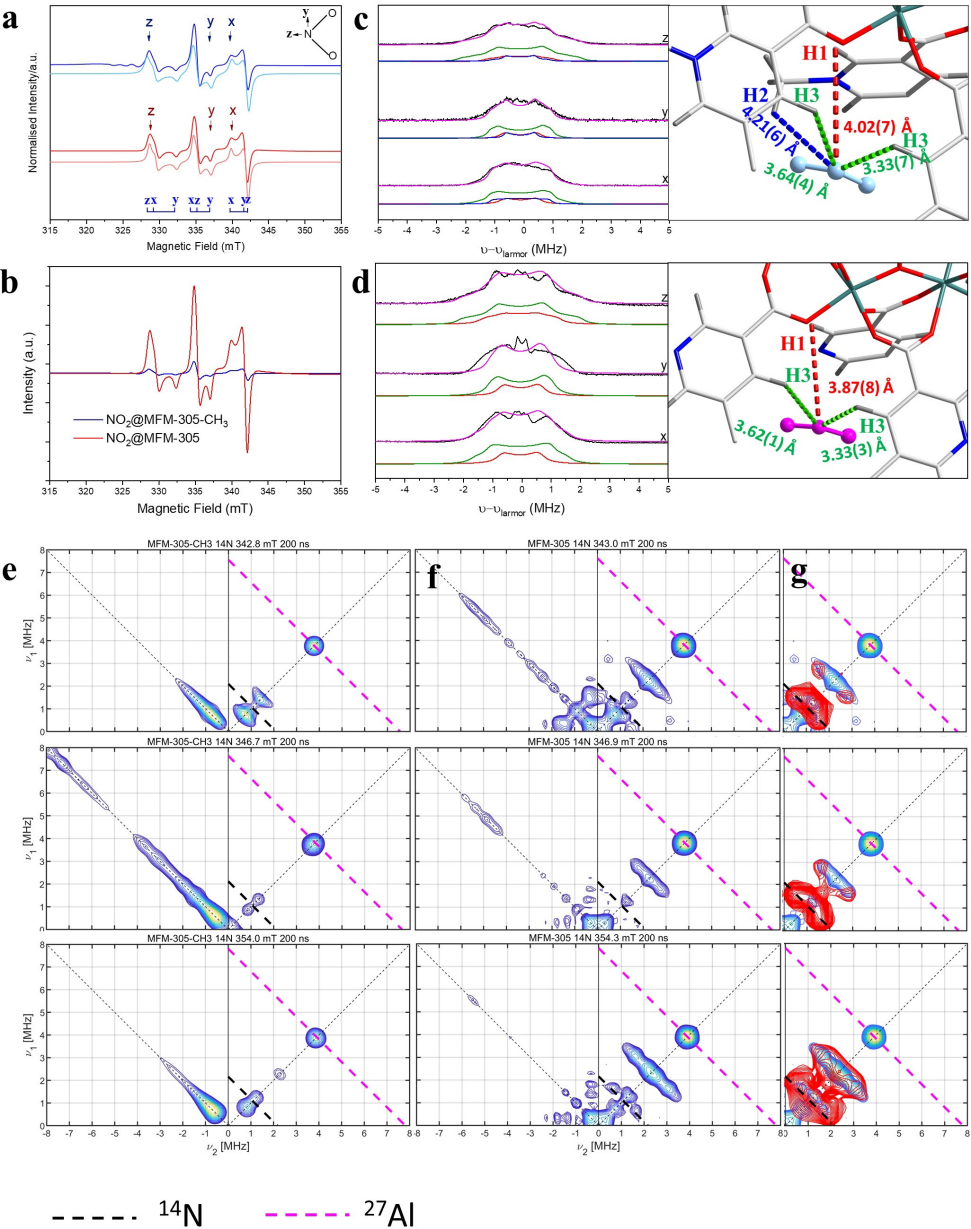
EPR spectroscopic data. a) Continuous‐wave X‐band (9.72 GHz) EPR spectrum of NO_2_@MFM‐305‐CH_3_ at 10 K (blue) and simulation (light blue) with *g_x_* =2.0063, *g_y_* =1.9910 and *g_z_* =2.0030 and ^14^N nuclear hyperfine interactions (nuclear spin, *I*=1) of *A_x_* =148, *A_y_* =133 and *A_z_* =194 MHz, where *x*, *y* and *z* define the NO_2_ molecular axes (inset). NO_2_ has *C*_2*v*_ point symmetry with the z axis along the C_2_ rotational axis, *y* parallel to the O−O vector and *x* normal to the NO_2_ plane; continuous‐wave X‐band (9.72 GHz) EPR spectrum of NO_2_@MFM‐305 at 10 K (red) and simulation (light red) with *g_x_* =2.006, *g_y_* =1.9913 and *g_z_* =2.0022 and ^14^N nuclear hyperfine interactions (nuclear spin, *I*=1) of *A_x_* =144, *A_y_* =133 and *A_z_* =188 MHz; b) comparison of intensity of EPR signal of NO_2_@MFM‐305‐CH_3_ and NO_2_@MFM‐305 under identical conditions; X‐band Davies ENDOR spectrum of c) NO_2_@ MFM‐305‐CH_3_ and d) NO_2_@MFM‐305 (black) at 5 K and the static magnetic fields indicated, shown by the arrows in (a), selecting the NO_2_ *x*, *y* and *z* axes (bottom to top), respectively. ENDOR gives pairs of transitions separated by the effective hyperfine coupling for the orientations selected, centred on the Larmor frequency of the nucleus being probed (14.9 MHz for ^1^H at 350 mT), and its simulated spectra (magenta for sum; red, green and blue for different protons); view of binding site of c) NO_2_ in MFM‐305‐CH_3_ and d) NO_2_ in MFM‐305 after movement and rotation attached; X‐band (9.7368 GHz) ^14^N HYSCORE spectra of e) NO_2_@MFM‐305‐CH_3_ measured at static fields 342.8, 346.7 and 354.0 mT, and of f) NO_2_@MFM‐305 measured at static fields 343.0, 346.9 and 354.3 mT; g) simulated spectra (red) with parameters in Table S7. The anti‐diagonal dashed lines cross the diagonal at the Larmor frequencies for ^14^N and ^27^Al (black: ^14^N; magenta: ^27^Al).

NO_2_ is a free radical and thus EPR spectroscopy was used to investigate the host–guest interactions in these two systems under high NO_2_ loading. EPR spectra of both NO_2_‐loaded MFM‐305‐CH_3_ and NO_2_‐loaded MFM‐305 at 10 K show signals from immobilised NO_2_ with resolution of the anisotropic electronic g‐factor and ^14^N hyperfine interaction (Figure 5a).^[33]^ These results confirm the presence of immobilised NO_2_, and for MFM‐305‐CH_3_, NO_2_ is adsorbed into the pores after the disproportionation reaction to form NOCl and NO_3_^−^ has reached equilibrium. Under identical NO_2_ loading conditions, the intensity of the NO_2_ signal is much lower in MFM‐305‐CH_3_ than in MFM‐305 due to the disproportionation reaction in MFM‐305‐CH_3_ (Figure 5b).

The interaction between NO_2_ and the framework and other guest molecules is revealed by Davies ENDOR (Electron‐Nuclear DOuble Resonance)^[34]^ and ^1^H and ^14^N HYSCORE (Hyperfine Sublevel Correlation) spectroscopies at 10 K. The ENDOR spectra of NO_2_‐loaded MFM‐305‐CH_3_ were taken at different static magnetic fields (Figure 5b) corresponding to different orientations of the NO_2_ molecules. Each spectrum contains features of multiple doublets of protons centred at the proton Larmor frequency. The spectra are dominated by frequencies of ca. 2 MHz which correspond to the nearest e⋅⋅⋅^1^H distances of 3.4 Å in a point dipolar approximation. When MFM‐305‐CH_3_ was synthesised with deuterated methyl groups (MFM‐305‐CD_3_), some weaker ^1^H couplings disappear (ca. 1 MHz; corresponding to distances of ca. 4.3 Å), and so these peaks must arise from the methyl protons (Figure S23). The orientation‐selective ENDOR measurements confirm that the largest ^1^H coupling to the nearest protons are observed along the C_2_ (z) axis of the NO_2_ molecule, hence the nearest NO_2_⋅⋅⋅^1^H distance is approximately in this direction. Calculated ENDOR spectra^[35]^ based on the SPXRD refined structure including the nearest CH_3_ and aromatic ^1^H positions gave couplings that are too large (Figure S19). The ENDOR spectra were measured at 10 K, and the SPXRD at room temperature, and it appears that on cooling the NO_2_ molecules move towards the centre of the pores. Calculated ENDOR spectra based on trial‐and‐error movement of the NO_2_ in the structural model gave good agreement on moving and rotating the NO_2_ to the centre of the pore with the nearest protons (H3) along the molecular C_2_ axis of NO_2_ (Figure 5c, Figure S18), although this model may not be unique. The ENDOR spectra of NO_2_‐loaded MFM‐305 is similar to that of NO_2_‐loaded MFM‐305‐CH_3_ but with a slightly larger ^1^H coupling, indicating the relative position of NO_2_ in these two materials are similar but the N⋅⋅⋅H(aromatic) distances in MFM‐305 are slightly shorter due to the absence of the methyl group (Figure 5d, Figure S21).

^1^H HYSCORE spectra for the above materials were collected at identical magnetic field positions and could be simulated with the same models (Figure S28, S29). In addition, weak hyperfine couplings were observed to ^14^N and ^27^Al nuclei in the HYSCORE, corresponding to remote nuclei (Figure 5e). In the spectra of NO_2_@MFM‐305‐CH_3_, a point‐like signal at ca. (3.8, 3.8) MHz represents the interaction between NO_2_ molecules and ^27^Al metal centres from the framework. For NO_2_@MFM‐305, a similar ^27^Al signal is observed but, in addition, intense cross‐peaks at ca. (2.8, 1.9) and (1.9, 2.8) MHz are observed corresponding to double‐quantum signals from a weakly coupled ^14^N nucleus. These could be reproduced with a point‐dipolar ^14^N hyperfine coupling |*A*(^14^N)|=[1.3, 1.3, −2.0] MHz, corresponding to an electron⋅⋅⋅^14^N distance of 1.85 Å (Table S7). These ^14^N signals are not observed in MFM‐305‐CH_3_, and hence cannot be from the pyridyl group but from other guest molecules, consistent with the higher NO_2_/N_2_O_4_ concentration in MFM‐305 (Figure 5f).

## Conclusion

Our studies reveal a new type of “regenerable reactive adsorption” of NO_2_ by modulating the charge in robust MOF materials. MFM‐305‐CH_3_ with a charged pore environment enables the in situ conversion of NO_2_ to the usable chemical species NOCl. A full investigation of the reactivity of adsorbed NO_2_ molecules, complex and dynamic host–guest binding and detailed structural investigation reveal key molecular details of the conversion of adsorbed NO_2_ molecules. The effective control of reactivity of NO_2_ achieved by MFM‐305‐CH_3_ provides new insights into the design of efficient protocols for abatement of corrosive air pollutants.

## Conflict of interest

The authors declare no conflict of interest.

1

## Supporting information

As a service to our authors and readers, this journal provides supporting information supplied by the authors. Such materials are peer reviewed and may be re‐organized for online delivery, but are not copy‐edited or typeset. Technical support issues arising from supporting information (other than missing files) should be addressed to the authors.

Supporting Information

Supporting Information

Supporting Information

Supporting Information

Supporting Information

## Data Availability

The data that support the findings of this study are available in the Supporting Information of this article.
