# Supplementary material for: Modulation of Uptake and Reactivity of Nitrogen Dioxide in Metal‐Organic Framework Materials
Source: Angew Chem Int Ed Engl. 2023 Jun 2;62(28):e202302602. doi: 10.1002/anie.202302602 (PMC10962595; doi:10.1002/anie.202302602)

checkCIF/PLATON report

You have not supplied any structure factors. As a result the full set of tests cannot be run.

THIS REPORT IS FOR GUIDANCE ONLY. IF USED AS PART OF A REVIEW PROCEDURE FOR PUBLICATION, IT SHOULD NOT REPLACE THE EXPERTISE OF AN EXPERIENCED CRYSTALLOGRAPHIC REFEREE.

No syntax errors found. CIF dictionary Interpreting this report

Datablock: TOPAS_MFM-305-Me-NO2

Bond precision:	C-C = 0.0063 A		Wavelength=0.82684
Cell:	a=21.4771(3)	b=21.4771(3)	c=10.84984(17)
	alpha=90	beta=90	gamma=90
Temperature:	298 K		

	Calculated	Reported
Volume	5004.66(16)	5004.64(17)
Space group	I 41/a m d	I41/amd:2
Hall group	-I 4bd 2	?
Moiety formula	C8 H7 Al N O5, 0.225(Cl N2 O4), 0.546(N O2), 0.775(Cl)	?
Sum formula	C8 H7 Al Cl N2 O7	C8 H7 Al Cl1 N2 O7
Mr	305.36	306.07
Dx,g cm-3	1.621	1.626
Z	16	16
Mu (mm-1)	0.610	0.000
F000	2478.2	0.0
F000’	2484.70	
h,k,lmax	50,50,25	
Nref	8623	
Tmin,Tmax		
Tmin’		

Correction method= Not given

Data completeness= 0.000 Theta(max)=

R(reflections)= wR2(reflections)=

S = Npar=

The following ALERTS were generated. Each ALERT has the format
test-name_ALERT_alert-type_alert-level.
Click on the hyperlinks for more details of the test.

Alert level A

PLAT430_ALERT_2_A Short Inter D...A Contact O2 ..N_3_3 . 1.85 Ang.
1-x,-1/2+y,-z = 20_645 Check

Author Response: The unrealistic short contact is due to the disorder inside the pore, of which occupancy is lower than 0.5. This means the possibility to observe this distance in the real crystal is neglectable.

PLAT430_ALERT_2_A Short Inter D...A Contact O2 ..O_2_3 . 2.23 Ang.
1-x,-1/2+y,-z = 20_645 Check

Author Response: The unrealistic short contact is due to the disorder inside the pore, of which occupancy is lower than 0.5. This means the possibility to observe this distance in the real crystal is neglectable.

PLAT430_ALERT_2_A Short Inter D...A Contact O3 ..O_2_3 . 2.49 Ang.
3/4-y,3/4-x,1/4-z = 21_665 Check

Author Response: The unrealistic short contact is due to the disorder inside the pore, of which occupancy is lower than 0.5. This means the possibility to observe this distance in the real crystal is neglectable.

Alert level B

PLAT430_ALERT_2_B Short Inter D...A Contact O2 ..O_3_3 . 2.78 Ang.
1-x,-1/2+y,-z = 20_645 Check

Author Response: The unrealistic short contact is due to the disorder inside the pore, of which occupancy is lower than 0.5. This means the possibility to observe this distance in the real crystal is neglectable.

PLAT430_ALERT_2_B Short Inter D...A Contact O3 ..N_3_3 . 2.63 Ang.
3/4-y,3/4-x,1/4-z = 21_665 Check

Author Response: The unrealistic short contact is due to the disorder inside the pore, of which occupancy is lower than 0.5. This means the possibility to observe this distance in the real crystal is neglectable.

```
PLAT430_ALERT_2_B Short Inter D...A Contact  O3       ..O_3_3    .       2.82 Ang.
                                       3/4-y,3/4-x,1/4-z  =     21_665 Check
```

Author Response: The unrealistic short contact is due to the disorder inside the pore, of which occupancy is lower than 0.5. This means the possibility to observe this distance in the real crystal is neglectable.

```
PLAT430_ALERT_2_B Short Inter D...A Contact  N5       ..O_1_2    .       2.59 Ang.
                                           1/2+x,y,1/2-z  =     16_555 Check
```

Author Response: The unrealistic short contact is due to the disorder inside the pore, of which occupancy is lower than 0.5. This means the possibility to observe this distance in the real crystal is neglectable.

```
PLAT430_ALERT_2_B Short Inter D...A Contact  N5       ..O_1_2    .       2.59 Ang.
                                           1/2-x,y,1/2-z  =      4_555 Check
```

Author Response: The unrealistic short contact is due to the disorder inside the pore, of which occupancy is lower than 0.5. This means the possibility to observe this distance in the real crystal is neglectable.

Alert level C

```
PLAT041_ALERT_1_C Calc. and Reported SumFormula    Strings  Differ     Please Check
PLAT043_ALERT_1_C Calculated and Reported Mol. Weight Differ by ..       0.71 Check
PLAT048_ALERT_1_C MoietyFormula Not Given (or Incomplete) ........     Please Check
PLAT125_ALERT_4_C No '_symmetry_space_group_name_Hall' Given .....     Please Do !
PLAT241_ALERT_2_C High   'MainMol' Ueq as Compared to Neighbors of         C1 Check
PLAT242_ALERT_2_C Low    'MainMol' Ueq as Compared to Neighbors of         O2 Check
PLAT340_ALERT_3_C Low Bond Precision on  C-C Bonds ..............    0.00625 Ang.
PLAT742_ALERT_1_C Angle   Calc    120.0(4), Rep      120.00 ......    Missing s.u.
              C1      -C3      -C4         1_555  1_555  1_555     #  726 Check
```

Alert level G

```
CELLZ01_ALERT_1_G Difference between formula and atom_site contents detected.
CELLZ01_ALERT_1_G ALERT: check formula stoichiometry or atom site occupancies.
           From the CIF: _cell_formula_units_Z   16
           From the CIF: _chemical_formula_sum  C8 H7 Al Cl1 N2 O7
           TEST: Compare cell contents of formula and atom_site data

           atom    Z*formula  cif sites diff
           C        128.00    128.00    0.00
           H        112.00    112.00    0.00
           Al        16.00     16.00    0.00
           Cl        16.00     16.00    0.00
           N         32.00     31.92    0.08
           O        112.00    111.85    0.15
PLAT004_ALERT_5_G Polymeric Structure Found with Maximum Dimension          3 Info
PLAT092_ALERT_4_G Check: Wavelength Given is not Cu,Ga,Mo,Ag,In Ka    0.82684 Ang.
```

```
PLAT302_ALERT_4_G Anion/Solvent/Minor-Residue Disorder (Resd  2  )       100% Note
PLAT302_ALERT_4_G Anion/Solvent/Minor-Residue Disorder (Resd  3  )       100% Note
PLAT302_ALERT_4_G Anion/Solvent/Minor-Residue Disorder (Resd  4  )       100% Note
PLAT432_ALERT_2_G Short Inter X...Y Contact  Cl_2_1   ..C2       .       3.20 Ang.
                                        3/4+y,3/4+x,1/4+z  =     12_665 Check
PLAT432_ALERT_2_G Short Inter X...Y Contact  O_1_2    ..C6       .       2.53 Ang.
                                            1/2-x,y,1/2-z  =      4_555 Check
PLAT432_ALERT_2_G Short Inter X...Y Contact  O_1_2    ..C4       .       2.82 Ang.
                                            1/2-x,y,1/2-z  =      4_555 Check
PLAT432_ALERT_2_G Short Inter X...Y Contact  O_2_2    ..C6       .       2.66 Ang.
                                      1/4-y,-1/4+x,-3/4+z  =      6_554 Check
PLAT432_ALERT_2_G Short Inter X...Y Contact  O_3_3    ..C4       .       2.92 Ang.
                                        3/4-y,3/4-x,1/4-z  =     21_665 Check
PLAT432_ALERT_2_G Short Inter X...Y Contact  O_2_3    ..C1       .       2.56 Ang.
                                             1-x,1/2+y,-z  =     20_655 Check
PLAT432_ALERT_2_G Short Inter X...Y Contact  O_2_3    ..C4       .       2.75 Ang.
                                        3/4-y,3/4-x,1/4-z  =     21_665 Check
PLAT432_ALERT_2_G Short Inter X...Y Contact  C1       ..N_3_3    .       2.50 Ang.
                                            1-x,-1/2+y,-z  =     20_645 Check
PLAT432_ALERT_2_G Short Inter X...Y Contact  C3       ..N_3_3    .       3.03 Ang.
                                            1-x,-1/2+y,-z  =     20_645 Check
PLAT720_ALERT_4_G Number of Unusual/Non-Standard Labels ..........         10 Note
PLAT779_ALERT_4_G Suspect or Irrelevant (Bond) Angle(s) in CIF ...      40.26 Deg.
              O1   -O1   -AL1     4_555   1_555   6_555 ......     #  179 Check
PLAT779_ALERT_4_G Suspect or Irrelevant (Bond) Angle(s) in CIF ...      40.26 Deg.
              O1   -O1   -AL1     9_555   1_555   1_555 ......     #  188 Check
PLAT779_ALERT_4_G Suspect or Irrelevant (Bond) Angle(s) in CIF ...      19.20 Deg.
              H1   -O1   -O1      9_555   1_555   9_555 ......     #  211 Check
PLAT779_ALERT_4_G Suspect or Irrelevant (Bond) Angle(s) in CIF ...      19.20 Deg.
              H1   -O1   -O1      4_555   1_555   4_555 ......     #  224 Check
PLAT779_ALERT_4_G Suspect or Irrelevant (Bond) Angle(s) in CIF ...      25.69 Deg.
              C1   -O1   -O2     10_555   1_555  10_555 ......     #  238 Check
PLAT779_ALERT_4_G Suspect or Irrelevant (Bond) Angle(s) in CIF ...      24.49 Deg.
              C1   -O1   -O3     10_555   1_555  10_555 ......     #  241 Check
PLAT779_ALERT_4_G Suspect or Irrelevant (Bond) Angle(s) in CIF ...      25.69 Deg.
              C1   -O1   -O2      1_555   1_555   1_555 ......     #  253 Check
PLAT779_ALERT_4_G Suspect or Irrelevant (Bond) Angle(s) in CIF ...      24.49 Deg.
              C1   -O1   -O3      1_555   1_555   1_555 ......     #  254 Check
PLAT779_ALERT_4_G Suspect or Irrelevant (Bond) Angle(s) in CIF ...       0.00 Deg.
              CL1  -O1   -H1      3_554   1_555   1_555 ......     #  306 Check
PLAT779_ALERT_4_G Suspect or Irrelevant (Bond) Angle(s) in CIF ...      39.00 Deg.
              O_2_3-H1   -CL1    17_445   1_555   3_554 ......     #  326 Check
PLAT779_ALERT_4_G Suspect or Irrelevant (Bond) Angle(s) in CIF ...      39.00 Deg.
              O_2_3-H1   -CL1    26_545   1_555   3_554 ......     #  333 Check
PLAT779_ALERT_4_G Suspect or Irrelevant (Bond) Angle(s) in CIF ...      39.60 Deg.
              O1   -H1   -AL1     9_555   1_555   1_555 ......     #  340 Check
PLAT779_ALERT_4_G Suspect or Irrelevant (Bond) Angle(s) in CIF ...      39.60 Deg.
              O1   -H1   -AL1     4_555   1_555   6_555 ......     #  348 Check
PLAT779_ALERT_4_G Suspect or Irrelevant (Bond) Angle(s) in CIF ...      18.70 Deg.
              H1   -H1   -O1      9_555   1_555   9_555 ......     #  353 Check
PLAT779_ALERT_4_G Suspect or Irrelevant (Bond) Angle(s) in CIF ...      18.70 Deg.
              H1   -H1   -O1      4_555   1_555   4_555 ......     #  363 Check
PLAT779_ALERT_4_G Suspect or Irrelevant (Bond) Angle(s) in CIF ...      30.32 Deg.
              H7   -N5   -C6      1_555   1_555   1_555 ......     #  401 Check
PLAT779_ALERT_4_G Suspect or Irrelevant (Bond) Angle(s) in CIF ...      26.30 Deg.
              H4   -N5   -C4      1_555   1_555   1_555 ......     #  406 Check
PLAT779_ALERT_4_G Suspect or Irrelevant (Bond) Angle(s) in CIF ...      26.30 Deg.
```

```
              H4   -N5   -C4      2_655   1_555   2_655 ......     #  412 Check
PLAT779_ALERT_4_G Suspect or Irrelevant (Bond) Angle(s) in CIF ...      25.98 Deg.
              H5   -N5   -C6      1_555   1_555   1_555 ......     #  416 Check
PLAT779_ALERT_4_G Suspect or Irrelevant (Bond) Angle(s) in CIF ...      25.98 Deg.
              H5   -N5   -C6      2_655   1_555   1_555 ......     #  423 Check
PLAT779_ALERT_4_G Suspect or Irrelevant (Bond) Angle(s) in CIF ...      30.04 Deg.
              C3   -N5   -C4      2_655   1_555   2_655 ......     #  433 Check
PLAT779_ALERT_4_G Suspect or Irrelevant (Bond) Angle(s) in CIF ...      30.04 Deg.
              C3   -N5   -C4      1_555   1_555   1_555 ......     #  441 Check
PLAT779_ALERT_4_G Suspect or Irrelevant (Bond) Angle(s) in CIF ...      41.60 Deg.
              O_1_2-N5   -H7      4_555   1_555   1_555 ......     #  449 Check
PLAT779_ALERT_4_G Suspect or Irrelevant (Bond) Angle(s) in CIF ...      36.20 Deg.
              O_1_2-N5   -O_1_2  16_555   1_555   4_555 ......     #  453 Check
PLAT779_ALERT_4_G Suspect or Irrelevant (Bond) Angle(s) in CIF ...      41.60 Deg.
              O_1_2-N5   -H7     16_555   1_555   1_555 ......     #  460 Check
PLAT779_ALERT_4_G Suspect or Irrelevant (Bond) Angle(s) in CIF ...      29.96 Deg.
              C2   -N5   -C3      1_555   1_555   1_555 ......     #  466 Check
PLAT779_ALERT_4_G Suspect or Irrelevant (Bond) Angle(s) in CIF ...      29.96 Deg.
              C2   -N5   -C3      1_555   1_555   2_655 ......     #  467 Check
PLAT779_ALERT_4_G Suspect or Irrelevant (Bond) Angle(s) in CIF ...      33.60 Deg.
              O_1_2-C6   -H7      4_555   1_555   1_555 ......     #  483 Check
PLAT779_ALERT_4_G Suspect or Irrelevant (Bond) Angle(s) in CIF ...      37.00 Deg.
              O_1_2-C6   -O_1_2  16_555   1_555   4_555 ......     #  486 Check
PLAT779_ALERT_4_G Suspect or Irrelevant (Bond) Angle(s) in CIF ...      33.60 Deg.
              O_1_2-C6   -H7     16_555   1_555   1_555 ......     #  488 Check
PLAT779_ALERT_4_G Suspect or Irrelevant (Bond) Angle(s) in CIF ...      27.33 Deg.
              C4   -C6   -N5      2_655   1_555   1_555 ......     #  493 Check
PLAT779_ALERT_4_G Suspect or Irrelevant (Bond) Angle(s) in CIF ...      27.33 Deg.
              C4   -C6   -N5      1_555   1_555   1_555 ......     #  500 Check
PLAT779_ALERT_4_G Suspect or Irrelevant (Bond) Angle(s) in CIF ...       9.40 Deg.
              O_2_2-C6   -O_2_2   7_656   1_555   9_556 ......     #  512 Check
PLAT779_ALERT_4_G Suspect or Irrelevant (Bond) Angle(s) in CIF ...      23.30 Deg.
              H4   -C6   -C4      2_655   1_555   2_655 ......     #  524 Check
PLAT779_ALERT_4_G Suspect or Irrelevant (Bond) Angle(s) in CIF ...      23.30 Deg.
              H4   -C6   -C4      1_555   1_555   1_555 ......     #  534 Check
PLAT779_ALERT_4_G Suspect or Irrelevant (Bond) Angle(s) in CIF ...      24.60 Deg.
              N_1_2-C6   -O_2_2   9_556   1_555   7_656 ......     #  544 Check
PLAT779_ALERT_4_G Suspect or Irrelevant (Bond) Angle(s) in CIF ...      22.50 Deg.
              N_1_2-C6   -O_2_2   9_556   1_555   9_556 ......     #  545 Check
PLAT779_ALERT_4_G Suspect or Irrelevant (Bond) Angle(s) in CIF ...      10.10 Deg.
              N_1_2-C6   -N_1_2   7_656   1_555   9_556 ......     #  554 Check
PLAT779_ALERT_4_G Suspect or Irrelevant (Bond) Angle(s) in CIF ...      22.50 Deg.
              N_1_2-C6   -O_2_2   7_656   1_555   7_656 ......     #  557 Check
PLAT779_ALERT_4_G Suspect or Irrelevant (Bond) Angle(s) in CIF ...      24.60 Deg.
              N_1_2-C6   -O_2_2   7_656   1_555   9_556 ......     #  558 Check
PLAT779_ALERT_4_G Suspect or Irrelevant (Bond) Angle(s) in CIF ...      27.90 Deg.
              C4   -C2   -C3      1_555   1_555   1_555 ......     #  571 Check
PLAT779_ALERT_4_G Suspect or Irrelevant (Bond) Angle(s) in CIF ...      27.90 Deg.
              C4   -C2   -C3      2_655   1_555   2_655 ......     #  574 Check
PLAT779_ALERT_4_G Suspect or Irrelevant (Bond) Angle(s) in CIF ...      32.71 Deg.
              C1   -C2   -C3      1_555   1_555   1_555 ......     #  580 Check
PLAT779_ALERT_4_G Suspect or Irrelevant (Bond) Angle(s) in CIF ...      32.71 Deg.
              C1   -C2   -C3      2_655   1_555   2_655 ......     #  585 Check
PLAT779_ALERT_4_G Suspect or Irrelevant (Bond) Angle(s) in CIF ...      27.11 Deg.
              O2   -C2   -C1      2_655   1_555   2_655 ......     #  588 Check
PLAT779_ALERT_4_G Suspect or Irrelevant (Bond) Angle(s) in CIF ...      27.11 Deg.
              O2   -C2   -C1      1_555   1_555   1_555 ......     #  597 Check
```

```
PLAT779_ALERT_4_G Suspect or Irrelevant (Bond) Angle(s) in CIF ...      28.91 Deg.
              N5   -C2   -C4       1_555   1_555   2_655 ......     #  607 Check
PLAT779_ALERT_4_G Suspect or Irrelevant (Bond) Angle(s) in CIF ...      28.91 Deg.
              N5   -C2   -C4       1_555   1_555   1_555 ......     #  608 Check
PLAT779_ALERT_4_G Suspect or Irrelevant (Bond) Angle(s) in CIF ...      35.50 Deg.
              N_3_3-C2   -O2      32_545   1_555   2_655 ......     #  614 Check
PLAT779_ALERT_4_G Suspect or Irrelevant (Bond) Angle(s) in CIF ...      35.50 Deg.
              N_3_3-C2   -O2      20_645   1_555   1_555 ......     #  624 Check
PLAT779_ALERT_4_G Suspect or Irrelevant (Bond) Angle(s) in CIF ...       9.00 Deg.
              CL_2_-C2   -CL_2_  22_745   1_555  28_445 ......     #  645 Check
PLAT779_ALERT_4_G Suspect or Irrelevant (Bond) Angle(s) in CIF ...      32.40 Deg.
              C3   -H2   -C2       1_555   1_555   1_555 ......     #  658 Check
PLAT779_ALERT_4_G Suspect or Irrelevant (Bond) Angle(s) in CIF ...      32.40 Deg.
              C3   -H2   -C2       2_655   1_555   1_555 ......     #  660 Check
PLAT779_ALERT_4_G Suspect or Irrelevant (Bond) Angle(s) in CIF ...      27.30 Deg.
              C1   -H2   -O2       1_555   1_555   1_555 ......     #  675 Check
PLAT779_ALERT_4_G Suspect or Irrelevant (Bond) Angle(s) in CIF ...      33.55 Deg.
              C1   -H2   -C3       1_555   1_555   1_555 ......     #  677 Check
PLAT779_ALERT_4_G Suspect or Irrelevant (Bond) Angle(s) in CIF ...      27.30 Deg.
              C1   -H2   -O2       2_655   1_555   2_655 ......     #  681 Check
PLAT779_ALERT_4_G Suspect or Irrelevant (Bond) Angle(s) in CIF ...      33.55 Deg.
              C1   -H2   -C3       2_655   1_555   2_655 ......     #  683 Check
PLAT779_ALERT_4_G Suspect or Irrelevant (Bond) Angle(s) in CIF ...      10.00 Deg.
              CL_2_-H2   -CL_2_  22_745   1_555  28_445 ......     #  694 Check
PLAT779_ALERT_4_G Suspect or Irrelevant (Bond) Angle(s) in CIF ...      38.70 Deg.
              N_3_3-H2   -O2      32_545   1_555   2_655 ......     #  708 Check
PLAT779_ALERT_4_G Suspect or Irrelevant (Bond) Angle(s) in CIF ...      38.70 Deg.
              N_3_3-H2   -O2      20_645   1_555   1_555 ......     #  720 Check
PLAT779_ALERT_4_G Suspect or Irrelevant (Bond) Angle(s) in CIF ...      26.30 Deg.
              H4   -C3   -C4       1_555   1_555   1_555 ......     #  729 Check
PLAT779_ALERT_4_G Suspect or Irrelevant (Bond) Angle(s) in CIF ...      24.40 Deg.
              H2   -C3   -C2       1_555   1_555   1_555 ......     #  732 Check
PLAT779_ALERT_4_G Suspect or Irrelevant (Bond) Angle(s) in CIF ...      30.00 Deg.
              O3   -C3   -C1       1_555   1_555   1_555 ......     #  736 Check
PLAT779_ALERT_4_G Suspect or Irrelevant (Bond) Angle(s) in CIF ...      28.23 Deg.
              O2   -C3   -C1       1_555   1_555   1_555 ......     #  742 Check
PLAT779_ALERT_4_G Suspect or Irrelevant (Bond) Angle(s) in CIF ...      33.20 Deg.
              C3   -C3   -C2       2_655   1_555   1_555 ......     #  750 Check
PLAT779_ALERT_4_G Suspect or Irrelevant (Bond) Angle(s) in CIF ...      29.96 Deg.
              N5   -C3   -C4       1_555   1_555   1_555 ......     #  759 Check
PLAT779_ALERT_4_G Suspect or Irrelevant (Bond) Angle(s) in CIF ...      29.98 Deg.
              C4   -C3   -N5       2_655   1_555   1_555 ......     #  760 Check
PLAT779_ALERT_4_G Suspect or Irrelevant (Bond) Angle(s) in CIF ...      30.05 Deg.
              C4   -C3   -C3       2_655   1_555   2_655 ......     #  761 Check
PLAT779_ALERT_4_G Suspect or Irrelevant (Bond) Angle(s) in CIF ...      37.90 Deg.
              N_3_3-C3   -O2      20_645   1_555   1_555 ......     #  772 Check
PLAT779_ALERT_4_G Suspect or Irrelevant (Bond) Angle(s) in CIF ...      30.00 Deg.
              C4   -C4   -N5       2_655   1_555   1_555 ......     #  783 Check
PLAT779_ALERT_4_G Suspect or Irrelevant (Bond) Angle(s) in CIF ...      28.91 Deg.
              C2   -C4   -C3       1_555   1_555   1_555 ......     #  786 Check
PLAT779_ALERT_4_G Suspect or Irrelevant (Bond) Angle(s) in CIF ...      31.59 Deg.
              C1   -C4   -C3       1_555   1_555   1_555 ......     #  791 Check
PLAT779_ALERT_4_G Suspect or Irrelevant (Bond) Angle(s) in CIF ...      32.67 Deg.
              C6   -C4   -N5       1_555   1_555   1_555 ......     #  798 Check
PLAT779_ALERT_4_G Suspect or Irrelevant (Bond) Angle(s) in CIF ...      26.65 Deg.
              O3   -C4   -C1       1_555   1_555   1_555 ......     #  801 Check
PLAT779_ALERT_4_G Suspect or Irrelevant (Bond) Angle(s) in CIF ...      22.43 Deg.
```

```
              H5   -C4   -C6      1_555   1_555   1_555 ......     #  808 Check
PLAT779_ALERT_4_G Suspect or Irrelevant (Bond) Angle(s) in CIF ...      12.00 Deg.
              O_2_3-C4   -H4     21_665   1_555   1_555 ......     #  823 Check
PLAT779_ALERT_4_G Suspect or Irrelevant (Bond) Angle(s) in CIF ...      31.03 Deg.
              C3   -C4   -C2      2_655   1_555   1_555 ......     #  829 Check
PLAT779_ALERT_4_G Suspect or Irrelevant (Bond) Angle(s) in CIF ...      30.05 Deg.
              C3   -C4   -C4      2_655   1_555   2_655 ......     #  830 Check
PLAT779_ALERT_4_G Suspect or Irrelevant (Bond) Angle(s) in CIF ...      43.90 Deg.
              O_3_3-C4   -O_2_3  21_665   1_555  21_665 ......     #  847 Check
PLAT779_ALERT_4_G Suspect or Irrelevant (Bond) Angle(s) in CIF ...      34.50 Deg.
              H7   -C4   -O_1_2   1_555   1_555   4_555 ......     #  858 Check
PLAT779_ALERT_4_G Suspect or Irrelevant (Bond) Angle(s) in CIF ...      34.46 Deg.
              H7   -C4   -H5      1_555   1_555   1_555 ......     #  861 Check
PLAT779_ALERT_4_G Suspect or Irrelevant (Bond) Angle(s) in CIF ...      21.10 Deg.
              H7   -C4   -C6      1_555   1_555   1_555 ......     #  863 Check
PLAT779_ALERT_4_G Suspect or Irrelevant (Bond) Angle(s) in CIF ...      39.84 Deg.
              H7   -C4   -N5      1_555   1_555   1_555 ......     #  868 Check
PLAT779_ALERT_4_G Suspect or Irrelevant (Bond) Angle(s) in CIF ...      22.20 Deg.
              N_3_3-C4   -O_3_3  21_665   1_555  21_665 ......     #  871 Check
PLAT779_ALERT_4_G Suspect or Irrelevant (Bond) Angle(s) in CIF ...      21.90 Deg.
              N_3_3-C4   -O_2_3  21_665   1_555  21_665 ......     #  874 Check
PLAT779_ALERT_4_G Suspect or Irrelevant (Bond) Angle(s) in CIF ...      33.10 Deg.
              N_3_3-C4   -H4     21_665   1_555   1_555 ......     #  883 Check
PLAT779_ALERT_4_G Suspect or Irrelevant (Bond) Angle(s) in CIF ...      33.70 Deg.
              N5   -H4   -C4      1_555   1_555   1_555 ......     #  886 Check
PLAT779_ALERT_4_G Suspect or Irrelevant (Bond) Angle(s) in CIF ...      33.80 Deg.
              C3   -H4   -C4      1_555   1_555   1_555 ......     #  889 Check
PLAT779_ALERT_4_G Suspect or Irrelevant (Bond) Angle(s) in CIF ...      29.70 Deg.
              N_3_3-H4   -O_2_3  21_665   1_555  21_665 ......     #  892 Check
PLAT779_ALERT_4_G Suspect or Irrelevant (Bond) Angle(s) in CIF ...      28.80 Deg.
              O_3_3-H4   -N_3_3  21_665   1_555  21_665 ......     #  907 Check
PLAT779_ALERT_4_G Suspect or Irrelevant (Bond) Angle(s) in CIF ...      26.20 Deg.
              C1   -H4   -O3      1_555   1_555   1_555 ......     #  922 Check
PLAT779_ALERT_4_G Suspect or Irrelevant (Bond) Angle(s) in CIF ...      33.98 Deg.
              C1   -H4   -C3      1_555   1_555   1_555 ......     #  925 Check
PLAT779_ALERT_4_G Suspect or Irrelevant (Bond) Angle(s) in CIF ...      22.04 Deg.
              C6   -H4   -H5      1_555   1_555   1_555 ......     #  930 Check
PLAT779_ALERT_4_G Suspect or Irrelevant (Bond) Angle(s) in CIF ...      35.62 Deg.
              C6   -H4   -N5      1_555   1_555   1_555 ......     #  936 Check
PLAT779_ALERT_4_G Suspect or Irrelevant (Bond) Angle(s) in CIF ...      33.40 Deg.
              H7   -H4   -O_1_2   1_555   1_555   4_555 ......     #  950 Check
PLAT779_ALERT_4_G Suspect or Irrelevant (Bond) Angle(s) in CIF ...      20.42 Deg.
              H7   -H4   -C6      1_555   1_555   1_555 ......     #  951 Check
PLAT779_ALERT_4_G Suspect or Irrelevant (Bond) Angle(s) in CIF ...      33.39 Deg.
              H7   -H4   -H5      1_555   1_555   1_555 ......     #  953 Check
PLAT779_ALERT_4_G Suspect or Irrelevant (Bond) Angle(s) in CIF ...      43.02 Deg.
              H7   -H4   -N5      1_555   1_555   1_555 ......     #  959 Check
PLAT779_ALERT_4_G Suspect or Irrelevant (Bond) Angle(s) in CIF ...      30.40 Deg.
              C2   -C1   -C3      1_555   1_555   1_555 ......     #  969 Check
PLAT779_ALERT_4_G Suspect or Irrelevant (Bond) Angle(s) in CIF ...      28.41 Deg.
              C4   -C1   -C3      1_555   1_555   1_555 ......     #  974 Check
PLAT779_ALERT_4_G Suspect or Irrelevant (Bond) Angle(s) in CIF ...      27.30 Deg.
              O_2_3-C1   -N_3_3  20_645   1_555  20_645 ......     #  979 Check
PLAT779_ALERT_4_G Suspect or Irrelevant (Bond) Angle(s) in CIF ...      23.90 Deg.
              H4   -C1   -C4      1_555   1_555   1_555 ......     #  984 Check
PLAT779_ALERT_4_G Suspect or Irrelevant (Bond) Angle(s) in CIF ...      23.60 Deg.
              H2   -C1   -C2      1_555   1_555   1_555 ......     #  993 Check
```

```
PLAT779_ALERT_4_G Suspect or Irrelevant (Bond) Angle(s) in CIF ...      36.80 Deg.
              AL1  -C1   -O2      1_555   1_555   1_555 ......    # 1005 Check
PLAT779_ALERT_4_G Suspect or Irrelevant (Bond) Angle(s) in CIF ...      36.44 Deg.
              O1   -C1   -AL1     1_555   1_555   1_555 ......    # 1007 Check
PLAT779_ALERT_4_G Suspect or Irrelevant (Bond) Angle(s) in CIF ...      35.78 Deg.
              AL1  -C1   -O1      6_555   1_555   1_555 ......    # 1017 Check
PLAT779_ALERT_4_G Suspect or Irrelevant (Bond) Angle(s) in CIF ...      27.60 Deg.
              AL1  -C1   -O3      6_555   1_555   1_555 ......    # 1027 Check
PLAT779_ALERT_4_G Suspect or Irrelevant (Bond) Angle(s) in CIF ...      38.90 Deg.
              O3   -C1   -AL1    10_555   1_555   1_555 ......    # 1030 Check
PLAT779_ALERT_4_G Suspect or Irrelevant (Bond) Angle(s) in CIF ...      32.60 Deg.
              O_2_3-O2   -N_3_3  20_645   1_555  20_645 ......    # 1044 Check
PLAT779_ALERT_4_G Suspect or Irrelevant (Bond) Angle(s) in CIF ...      23.20 Deg.
              O3   -O2   -C1      1_555   1_555   1_555 ......    # 1049 Check
PLAT779_ALERT_4_G Suspect or Irrelevant (Bond) Angle(s) in CIF ...      34.80 Deg.
              C3   -O2   -C1      1_555   1_555   1_555 ......    # 1054 Check
PLAT779_ALERT_4_G Suspect or Irrelevant (Bond) Angle(s) in CIF ...      41.18 Deg.
              O1   -O2   -AL1     1_555   1_555   1_555 ......    # 1073 Check
PLAT779_ALERT_4_G Suspect or Irrelevant (Bond) Angle(s) in CIF ...      23.40 Deg.
              C2   -O2   -H2      1_555   1_555   1_555 ......    # 1087 Check
PLAT779_ALERT_4_G Suspect or Irrelevant (Bond) Angle(s) in CIF ...      31.06 Deg.
              C2   -O2   -C3      1_555   1_555   1_555 ......    # 1089 Check
PLAT779_ALERT_4_G Suspect or Irrelevant (Bond) Angle(s) in CIF ...      19.00 Deg.
              O_3_3-O2   -N_3_3  20_645   1_555  20_645 ......    # 1104 Check
PLAT779_ALERT_4_G Suspect or Irrelevant (Bond) Angle(s) in CIF ...      44.40 Deg.
              O3   -O2   -AL1     9_555   1_555   1_555 ......    # 1115 Check
PLAT779_ALERT_4_G Suspect or Irrelevant (Bond) Angle(s) in CIF ...      39.30 Deg.
              C3   -O3   -C1      1_555   1_555   1_555 ......    # 1120 Check
PLAT779_ALERT_4_G Suspect or Irrelevant (Bond) Angle(s) in CIF ...      24.50 Deg.
              O2   -O3   -C1      1_555   1_555   1_555 ......    # 1123 Check
PLAT779_ALERT_4_G Suspect or Irrelevant (Bond) Angle(s) in CIF ...      40.70 Deg.
              O_2_3-O3   -H4     21_665   1_555   1_555 ......    # 1128 Check
PLAT779_ALERT_4_G Suspect or Irrelevant (Bond) Angle(s) in CIF ...      43.80 Deg.
              O1   -O3   -AL1     1_555   1_555   6_555 ......    # 1137 Check
PLAT779_ALERT_4_G Suspect or Irrelevant (Bond) Angle(s) in CIF ...      26.80 Deg.
              N_3_3-O3   -O_2_3  21_665   1_555  21_665 ......    # 1140 Check
PLAT779_ALERT_4_G Suspect or Irrelevant (Bond) Angle(s) in CIF ...      24.40 Deg.
              C4   -O3   -H4      1_555   1_555   1_555 ......    # 1149 Check
PLAT779_ALERT_4_G Suspect or Irrelevant (Bond) Angle(s) in CIF ...      31.30 Deg.
              C4   -O3   -C3      1_555   1_555   1_555 ......    # 1151 Check
PLAT779_ALERT_4_G Suspect or Irrelevant (Bond) Angle(s) in CIF ...      19.60 Deg.
              O1   -O3   -H1      4_555   1_555   4_555 ......    # 1163 Check
PLAT779_ALERT_4_G Suspect or Irrelevant (Bond) Angle(s) in CIF ...      40.70 Deg.
              O1   -O3   -AL1     4_555   1_555   6_555 ......    # 1171 Check
PLAT779_ALERT_4_G Suspect or Irrelevant (Bond) Angle(s) in CIF ...      39.90 Deg.
              O2   -O3   -N_3_3   6_555   1_555  21_665 ......    # 1188 Check
PLAT779_ALERT_4_G Suspect or Irrelevant (Bond) Angle(s) in CIF ...      24.90 Deg.
              O_3_3-O3   -N_3_3  21_665   1_555  21_665 ......    # 1201 Check
PLAT779_ALERT_4_G Suspect or Irrelevant (Bond) Angle(s) in CIF ...      24.65 Deg.
              C1   -O3   -O2     10_555   1_555  10_555 ......    # 1211 Check
PLAT779_ALERT_4_G Suspect or Irrelevant (Bond) Angle(s) in CIF ...      22.80 Deg.
              O3   -O3   -C1     10_555   1_555  10_555 ......    # 1223 Check
PLAT779_ALERT_4_G Suspect or Irrelevant (Bond) Angle(s) in CIF ...      36.45 Deg.
              AL1  -O3   -O3      1_555   1_555  10_555 ......    # 1255 Check
PLAT779_ALERT_4_G Suspect or Irrelevant (Bond) Angle(s) in CIF ...      34.10 Deg.
              AL1  -O3   -O1      1_555   1_555   1_555 ......    # 1264 Check
PLAT779_ALERT_4_G Suspect or Irrelevant (Bond) Angle(s) in CIF ...      37.80 Deg.
```

```
             AL1   -O3    -O2       1_555    1_555    1_555 ......     # 1267 Check
PLAT780_ALERT_1_G Coordinates do not Form a Properly Connected Set     Please Do !
PLAT811_ALERT_5_G No ADDSYM Analysis: Too Many Excluded Atoms ....          ! Info
```

```
   3 ALERT level A = Most likely a serious problem - resolve or explain
   5 ALERT level B = A potentially serious problem, consider carefully
   8 ALERT level C = Check. Ensure it is not caused by an omission or oversight
 151 ALERT level G = General information/check it is not something unexpected

   7 ALERT type 1 CIF construction/syntax error, inconsistent or missing data
  19 ALERT type 2 Indicator that the structure model may be wrong or deficient
   1 ALERT type 3 Indicator that the structure quality may be low
 138 ALERT type 4 Improvement, methodology, query or suggestion
   2 ALERT type 5 Informative message, check
```

It is advisable to attempt to resolve as many as possible of the alerts in all categories. Often the minor alerts point to easily fixed oversights, errors and omissions in your CIF or refinement strategy, so attention to these fine details can be worthwhile. In order to resolve some of the more serious problems it may be necessary to carry out additional measurements or structure refinements. However, the purpose of your study may justify the reported deviations and the more serious of these should normally be commented upon in the discussion or experimental section of a paper or in the "special_details" fields of the CIF. checkCIF was carefully designed to identify outliers and unusual parameters, but every test has its limitations and alerts that are not important in a particular case may appear. Conversely, the absence of alerts does not guarantee there are no aspects of the results needing attention. It is up to the individual to critically assess their own results and, if necessary, seek expert advice.

Publication of your CIF in IUCr journals

A basic structural check has been run on your CIF. These basic checks will be run on all CIFs submitted for publication in IUCr journals (*Acta Crystallographica*, *Journal of Applied Crystallography*, *Journal of Synchrotron Radiation*); however, if you intend to submit to *Acta Crystallographica Section C* or *E* or *IUCrData*, you should make sure that full publication checks are run on the final version of your CIF prior to submission.

Publication of your CIF in other journals

Please refer to the *Notes for Authors* of the relevant journal for any special instructions relating to CIF submission.

PLATON version of 12/09/2022; check.def file version of 09/08/2022

Datablock TOPAS_MFM-305-Me-NO2 - ellipsoid plot

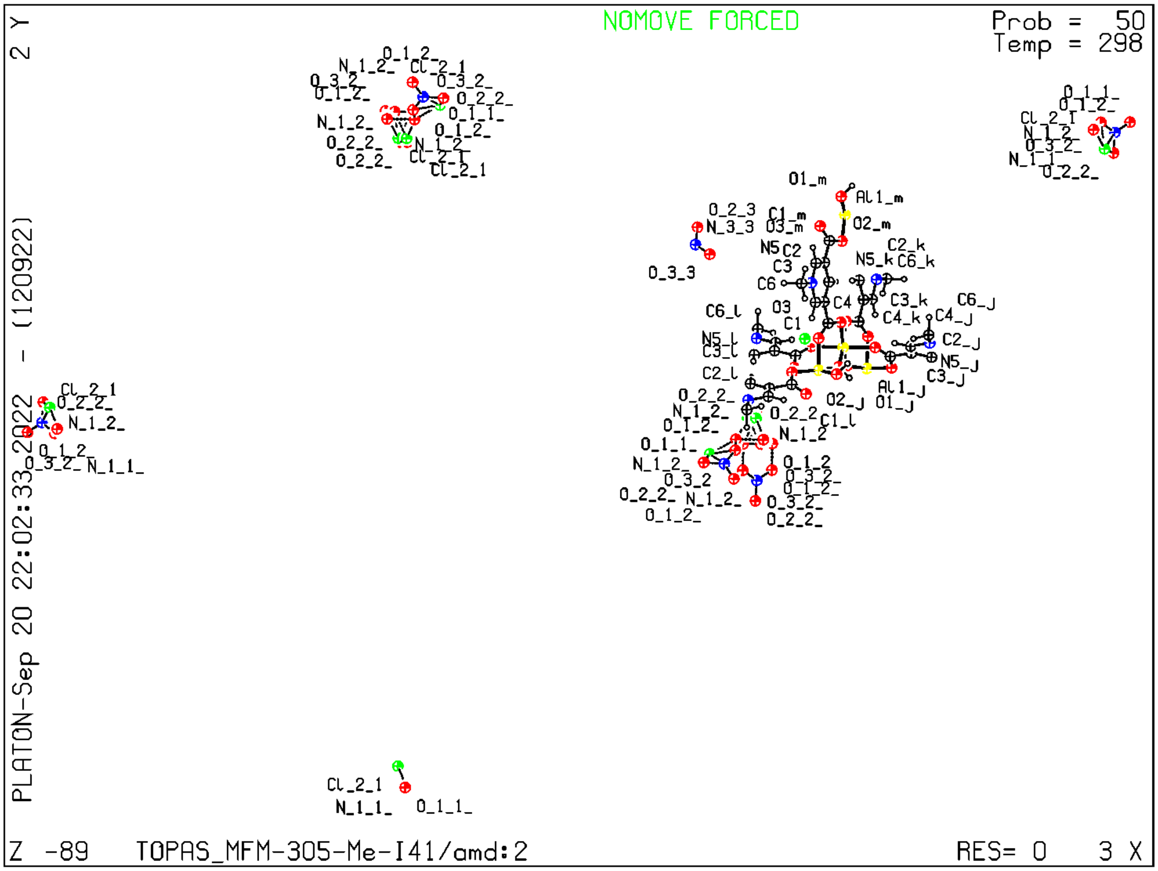

Supplement: Supplementary file 1 — Supporting Information [file ANIE-62-0-s002.pdf]
