# Supplementary material for: Modulation of Uptake and Reactivity of Nitrogen Dioxide in Metal‐Organic Framework Materials
Source: Angew Chem Int Ed Engl. 2023 Jun 2;62(28):e202302602. doi: 10.1002/anie.202302602 (PMC10962595; doi:10.1002/anie.202302602)

checkCIF/PLATON report

You have not supplied any structure factors. As a result the full set of tests cannot be run.

THIS REPORT IS FOR GUIDANCE ONLY. IF USED AS PART OF A REVIEW PROCEDURE FOR PUBLICATION, IT SHOULD NOT REPLACE THE EXPERTISE OF AN EXPERIENCED CRYSTALLOGRAPHIC REFEREE.

No syntax errors found. CIF dictionary Interpreting this report

Datablock: MFM-305-NO2

```
Bond precision:   C-C = 0.0115 A          Wavelength=0.82684

Cell:              a=21.5132(6)      b=21.5132(6)      c=10.7854(3)
                   alpha=90          beta=90           gamma=90
Temperature:       298 K

                 Calculated                        Reported
Volume           4991.7(3)                         4991.6(3)
Space group      I 41/a m d                        I41/amd:2
Hall group       -I 4bd 2                          ?
                 C7 H4 Al N O5, 0.056(N8
Moiety formula   O16), 0.044(N4 O8),               ?
                 0.605(N2 O4), 1.658
Sum formula      C7 H4 Al N4.50 O12                C7 H4 Al N4.50 O12
Mr               369.94                            369.94
Dx,g cm-3        1.969                             1.969
Z                16                                16
Mu (mm-1)        0.373                             0.000
F000             2982.5                            0.0
F000'            2987.28
h,k,lmax         50,50,25
Nref             8600
Tmin,Tmax
Tmin'

Correction method= Not given

Data completeness= 0.000          Theta(max)=

R(reflections)=                                  wR2(reflections)=
S =                          Npar=
```

The following ALERTS were generated. Each ALERT has the format
test-name_ALERT_alert-type_alert-level.
Click on the hyperlinks for more details of the test.

Alert level A

PLAT430_ALERT_2_A Short Inter D...A Contact O_1_1 ..O_1_1 . 1.76 Ang.
-1/4+y,1/4+x,./4-z = 26_558 Check

Author Response: The unrealistic short contact is due to the disorder inside the pore, of which occupancy is lower than 0.5. This means the possibility to observe this distance in the real crystal is neglectable.

PLAT430_ALERT_2_A Short Inter D...A Contact O_1_1 ..N_1_1 . 2.51 Ang.
-1/4+y,1/4+x,./4-z = 26_558 Check

Author Response: The unrealistic short contact is due to the disorder inside the pore, of which occupancy is lower than 0.5. This means the possibility to observe this distance in the real crystal is neglectable.

PLAT430_ALERT_2_A Short Inter D...A Contact O2 ..N_1_1 . 2.54 Ang.
1-x,-1+y,-1+z = 2_644 Check

Author Response: The unrealistic short contact is due to the disorder inside the pore, of which occupancy is lower than 0.5. This means the possibility to observe this distance in the real crystal is neglectable.

PLAT430_ALERT_2_A Short Inter D...A Contact O_2_1 ..O_1_2 . 2.06 Ang.
1/2-x,1-y,3/2+z = 3_566 Check

Author Response: The unrealistic short contact is due to the disorder inside the pore, of which occupancy is lower than 0.5. This means the possibility to observe this distance in the real crystal is neglectable.

PLAT430_ALERT_2_A Short Inter D...A Contact O_2_1 ..N_1_2 . 2.46 Ang.
1/2-x,1-y,3/2+z = 3_566 Check

Author Response: The unrealistic short contact is due to the disorder inside the pore, of which occupancy is lower than 0.5. This means the possibility to observe this distance in the real crystal is neglectable.

PLAT430_ALERT_2_A Short Inter D...A Contact O3 ..O_1_2 . 2.00 Ang.
1/4+y,1/4+x,3/4+z = 28_556 Check

Author Response: The unrealistic short contact is due to the disorder inside the pore, of which occupancy is lower than 0.5. This means the possibility to observe this distance in the real crystal is neglectable.

```
PLAT430_ALERT_2_A Short Inter D...A Contact  O_2_2     ..O_2_2     .       2.30 Ang.
                                                -x,y,z  =      2_555 Check
```

Author Response: The unrealistic short contact is due to the disorder inside the pore, of which occupancy is lower than 0.5. This means the possibility to observe this distance in the real crystal is neglectable.

```
PLAT430_ALERT_2_A Short Inter D...A Contact  N5        ..O_4       .       2.10 Ang.
                                      3/2-x,1-y,-3/2+z  =      3_663 Check
```

Author Response: The unrealistic short contact is due to the disorder inside the pore, of which occupancy is lower than 0.5. This means the possibility to observe this distance in the real crystal is neglectable.

```
PLAT430_ALERT_2_A Short Inter D...A Contact  N5        ..O_4       .       2.10 Ang.
                                     -1/2+x,1-y,-3/2+z  =     15_463 Check
```

Author Response: The unrealistic short contact is due to the disorder inside the pore, of which occupancy is lower than 0.5. This means the possibility to observe this distance in the real crystal is neglectable.

```
PLAT430_ALERT_2_A Short Inter D...A Contact  N5        ..O_3_2     .       2.20 Ang.
                                          x,-1/2+y,2-z  =     32_547 Check
```

Author Response: The unrealistic short contact is due to the disorder inside the pore, of which occupancy is lower than 0.5. This means the possibility to observe this distance in the real crystal is neglectable.

```
PLAT430_ALERT_2_A Short Inter D...A Contact  N5        ..O_3_2     .       2.20 Ang.
                                        1-x,-1/2+y,2-z  =     20_647 Check
```

Author Response: The unrealistic short contact is due to the disorder inside the pore, of which occupancy is lower than 0.5. This means the possibility to observe this distance in the real crystal is neglectable.

```
PLAT430_ALERT_2_A Short Inter D...A Contact  O_2       ..O_2       .       2.15 Ang.
                                     7/4-y,7/4-x,y/4-z  =     21_778 Check
```

Author Response: The unrealistic short contact is due to the disorder inside the pore, of which occupancy is lower than 0.5. This means the possibility to observe this distance in the real crystal is neglectable.

```
PLAT430_ALERT_2_A Short  Inter D...A Contact  O_2      ..O_2      .       2.48 Ang.
                                       7/4-y,-1/4+x,y/4-z  =      8_758 Check
```

Author Response: The unrealistic short contact is due to the disorder inside the pore, of which occupancy is lower than 0.5. This means the possibility to observe this distance in the real crystal is neglectable.

```
PLAT430_ALERT_2_A Short  Inter D...A Contact  O_2      ..O_2      .       2.48 Ang.
                                        1/4+y,7/4-x,y/4-z  =     27_578 Check
```

Author Response: The unrealistic short contact is due to the disorder inside the pore, of which occupancy is lower than 0.5. This means the possibility to observe this distance in the real crystal is neglectable.

```
PLAT430_ALERT_2_A Short  Inter D...A Contact  O_3      ..O_3      .       2.32 Ang.
                                        7/4-y,7/4-x,+/4-z  =     21_779 Check
```

Author Response: The unrealistic short contact is due to the disorder inside the pore, of which occupancy is lower than 0.5. This means the possibility to observe this distance in the real crystal is neglectable.

Alert level B

```
PLAT340_ALERT_3_B Low Bond Precision on  C-C Bonds ...............     0.0115 Ang.
```

Author Response: It is a case solved by powder X-ray diffraction and with highly disordered guest molecule. The bond precision is high enough to demonstrate the inter -action between the host framework and the guest molecule.

```
PLAT430_ALERT_2_B Short  Inter D...A Contact  O_1_1    ..O_2_1    .       2.74 Ang.
                                       -1/4+y,1/4+x,./4-z  =     26_558 Check
```

Author Response: The unrealistic short contact is due to the disorder inside the pore, of which occupancy is lower than 0.5. This means the possibility to observe this distance in the real crystal is neglectable.

```
PLAT430_ALERT_2_B Short  Inter D...A Contact  O2       ..O_1_2    .       2.70 Ang.
                                           1/2+x,-y,1/2+z  =     15_555 Check
```

Author Response: The unrealistic short contact is due to the disorder inside the pore, of which occupancy is lower than 0.5. This means the possibility to observe this distance in the real crystal is neglectable.

PLAT430_ALERT_2_B Short Inter D...A Contact O_1_2 ..N_1_1 . 2.69 Ang.
1/2-x,1-y,-3/2+z = 3_563 Check

Author Response: The unrealistic short contact is due to the disorder inside the pore, of which occupancy is lower than 0.5. This means the possibility to observe this distance in the real crystal is neglectable.

PLAT430_ALERT_2_B Short Inter D...A Contact O_2_2 ..N_1_2 . 2.86 Ang.
-x,y,z = 2_555 Check

Author Response: The unrealistic short contact is due to the disorder inside the pore, of which occupancy is lower than 0.5. This means the possibility to observe this distance in the real crystal is neglectable.

PLAT430_ALERT_2_B Short Inter D...A Contact N5 ..O_3 . 2.87 Ang.
3/2-x,1-y,-3/2+z = 3_663 Check

Author Response: The unrealistic short contact is due to the disorder inside the pore, of which occupancy is lower than 0.5. This means the possibility to observe this distance in the real crystal is neglectable.

PLAT430_ALERT_2_B Short Inter D...A Contact N5 ..O_3 . 2.87 Ang.
-1/2+x,1-y,-3/2+z = 15_463 Check

Author Response: The unrealistic short contact is due to the disorder inside the pore, of which occupancy is lower than 0.5. This means the possibility to observe this distance in the real crystal is neglectable.

PLAT430_ALERT_2_B Short Inter D...A Contact O_2 ..O_2 . 2.77 Ang.
1/4+y,-1/4+x,y/4-z = 10_558 Check

Author Response: The unrealistic short contact is due to the disorder inside the pore, of which occupancy is lower than 0.5. This means the possibility to observe this distance in the real crystal is neglectable.

PLAT430_ALERT_2_B Short Inter D...A Contact O_2 ..O_4 . 2.80 Ang.
2-x,y,z = 2_755 Check

Author Response: The unrealistic short contact is due to the disorder inside the pore, of which occupancy is lower than 0.5. This means the possibility to observe this distance in the real crystal is neglectable.

PLAT430_ALERT_2_B Short Inter D...A Contact O_4 ..O_4 . 2.80 Ang.
x,3/2-y,z = 31_565 Check

Author Response: The unrealistic short contact is due to the disorder inside the pore, of which occupancy is lower than 0.5. This means the possibility to observe this distance in the real crystal is neglectable.

```
PLAT430_ALERT_2_B Short Inter D...A Contact  O_4      ..O_4      .       2.80 Ang.
                                              2-x,3/2-y,z  =     19_765 Check
```

Author Response: The unrealistic short contact is due to the disorder inside the pore, of which occupancy is lower than 0.5. This means the possibility to observe this distance in the real crystal is neglectable.

Alert level C

```
PLAT048_ALERT_1_C MoietyFormula Not Given (or Incomplete) ........     Please Check
PLAT125_ALERT_4_C No '_symmetry_space_group_name_Hall' Given .....     Please Do !
PLAT241_ALERT_2_C High   'MainMol' Ueq as Compared to Neighbors of         C1 Check
PLAT242_ALERT_2_C Low    'MainMol' Ueq as Compared to Neighbors of         O2 Check
PLAT351_ALERT_3_C Long   C-H (X0.96,N1.08A)  C2       - H2       .       1.11 Ang.
PLAT351_ALERT_3_C Long   C-H (X0.96,N1.08A)  C4       - H4       .       1.11 Ang.
PLAT430_ALERT_2_C Short Inter D...A Contact  O_1      ..O_3      .       2.87 Ang.
                                                    x,y,z  =      1_555 Check
```

Author Response: The unrealistic short contact is due to the disorder inside the pore, of which occupancy is lower than 0.5. This means the possibility to observe this distance in the real crystal is neglectable.

```
PLAT701_ALERT_1_C Bond    Calc     1.30(3), Rep   1.261(14), Dev..       1.30 Sigma
              C1       -O3             1_555   1_555 ........     #    9 Check
PLAT702_ALERT_1_C Angle   Calc    93.8(11), Rep    92.6(10), Dev..       1.09 Sigma
              C4      -C1      -O3         1_555  1_555  1_555     #   15 Check
```

Alert level G

```
CELLZ01_ALERT_1_G Difference between formula and atom_site contents detected.
CELLZ01_ALERT_1_G ALERT: check formula stoichiometry or atom site occupancies.
           From the CIF: _cell_formula_units_Z   16
           From the CIF: _chemical_formula_sum  C7 H4 Al N4.50 O12
           TEST: Compare cell contents of formula and atom_site data

           atom    Z*formula  cif sites diff
           C        112.00    112.00    0.00
           H         64.00     64.00    0.00
           Al        16.00     16.00    0.00
           N         72.00     71.94    0.06
           O        192.00    191.87    0.13
PLAT004_ALERT_5_G Polymeric Structure Found with Maximum Dimension          3 Info
PLAT092_ALERT_4_G Check: Wavelength Given is not Cu,Ga,Mo,Ag,In Ka    0.82684 Ang.
PLAT301_ALERT_3_G Main Residue  Disorder ..............(Resd  2  )       100% Note
PLAT302_ALERT_4_G Anion/Solvent/Minor-Residue Disorder (Resd  3  )       100% Note
PLAT302_ALERT_4_G Anion/Solvent/Minor-Residue Disorder (Resd  4  )       100% Note
```

```
PLAT302_ALERT_4_G Anion/Solvent/Minor-Residue Disorder (Resd  5  )       100% Note
PLAT302_ALERT_4_G Anion/Solvent/Minor-Residue Disorder (Resd  6  )       100% Note
PLAT395_ALERT_2_G Deviating  X-O-Y   Angle From 120 for O_3_2    .       86.9 Degree
PLAT395_ALERT_2_G Deviating  X-O-Y   Angle From 120 for O_2_2    .      146.2 Degree
PLAT432_ALERT_2_G Short Inter X...Y Contact  O_1_1    ..C1       .       2.70 Ang.
                                              1-x,1+y,1+z  =      2_666 Check
PLAT432_ALERT_2_G Short Inter X...Y Contact  O_1_1    ..C4       .       2.86 Ang.
                                              1-x,1-y,2-z  =     14_667 Check
PLAT432_ALERT_2_G Short Inter X...Y Contact  O_2_1    ..C4       .       2.83 Ang.
                                        3/4-y,5/4-x,3/4+z  =      7_666 Check
PLAT432_ALERT_2_G Short Inter X...Y Contact  O_1_2    ..C4       .       2.50 Ang.
                                     -1/4+y,-1/4+x,-3/4+z  =     12_554 Check
PLAT432_ALERT_2_G Short Inter X...Y Contact  O_1_2    ..C1       .       2.60 Ang.
                                     -1/4+y,-1/4+x,-3/4+z  =     12_554 Check
PLAT432_ALERT_2_G Short Inter X...Y Contact  O_1_2    ..C3       .       2.70 Ang.
                                     -1/4+y,-1/4+x,-3/4+z  =     12_554 Check
PLAT432_ALERT_2_G Short Inter X...Y Contact  N_1_1    ..C1       .       2.62 Ang.
                                              1-x,1+y,1+z  =      2_666 Check
PLAT432_ALERT_2_G Short Inter X...Y Contact  N_1_1    ..C3       .       2.76 Ang.
                                              1-x,1+y,1+z  =      2_666 Check
PLAT432_ALERT_2_G Short Inter X...Y Contact  N_1_2    ..C4       .       2.75 Ang.
                                     -1/4+y,-1/4+x,-3/4+z  =     12_554 Check
PLAT432_ALERT_2_G Short Inter X...Y Contact  N_1_2    ..C3       .       2.89 Ang.
                                     -1/4+y,-1/4+x,-3/4+z  =     12_554 Check
PLAT432_ALERT_2_G Short Inter X...Y Contact  N5       ..N_2_2    .       2.39 Ang.
                                             x,-1/2+y,2-z  =     32_547 Check
PLAT432_ALERT_2_G Short Inter X...Y Contact  N5       ..N_2_2    .       2.39 Ang.
                                           1-x,-1/2+y,2-z  =     20_647 Check
PLAT432_ALERT_2_G Short Inter X...Y Contact  N5       ..N_2      .       2.50 Ang.
                                         3/2-x,1-y,-3/2+z  =      3_663 Check
PLAT432_ALERT_2_G Short Inter X...Y Contact  N5       ..N_2      .       2.50 Ang.
                                        -1/2+x,1-y,-3/2+z  =     15_463 Check
PLAT432_ALERT_2_G Short Inter X...Y Contact  O_3_2    ..C4       .       2.70 Ang.
                                            1-x,1/2+y,2-z  =     20_657 Check
PLAT432_ALERT_2_G Short Inter X...Y Contact  O_1      ..N_2      .       2.56 Ang.
                                                    x,y,z  =      1_555 Check
PLAT432_ALERT_2_G Short Inter X...Y Contact  O_2      ..N_2      .       2.57 Ang.
                                                    x,y,z  =      1_555 Check
PLAT432_ALERT_2_G Short Inter X...Y Contact  O_3      ..N_1      .       2.56 Ang.
                                                    x,y,z  =      1_555 Check
PLAT432_ALERT_2_G Short Inter X...Y Contact  O_3      ..C4       .       2.67 Ang.
                                          1/2+x,1-y,3/2+z  =     15_566 Check
PLAT432_ALERT_2_G Short Inter X...Y Contact  O_4      ..N_1      .       2.50 Ang.
                                                  2-x,y,z  =      2_755 Check
PLAT432_ALERT_2_G Short Inter X...Y Contact  O_4      ..N_1      .       2.60 Ang.
                                                    x,y,z  =      1_555 Check
PLAT432_ALERT_2_G Short Inter X...Y Contact  O_4      ..C4       .       2.70 Ang.
                                          3/2-x,1-y,3/2+z  =      3_666 Check
PLAT432_ALERT_2_G Short Inter X...Y Contact  O_4      ..C4       .       2.70 Ang.
                                          1/2+x,1-y,3/2+z  =     15_566 Check
PLAT432_ALERT_2_G Short Inter X...Y Contact  C4       ..N_2      .       2.50 Ang.
                                        -1/2+x,1-y,-3/2+z  =     15_463 Check
PLAT432_ALERT_2_G Short Inter X...Y Contact  C4       ..N_2_2    .       2.84 Ang.
                                           1-x,-1/2+y,2-z  =     20_647 Check
PLAT432_ALERT_2_G Short Inter X...Y Contact  N_1      ..N_2      .       1.79 Ang.
                                                    x,y,z  =      1_555 Check
PLAT432_ALERT_2_G Short Inter X...Y Contact  N_1      ..N_2      .       2.62 Ang.
```

```
                                                 x,3/2-y,z  =     31_565 Check
PLAT432_ALERT_2_G Short Inter X...Y Contact  N_2      ..N_2      .       2.60 Ang.
                                                 x,3/2-y,z  =     31_565 Check
PLAT720_ALERT_4_G Number of Unusual/Non-Standard Labels .........          15 Note
PLAT779_ALERT_4_G Suspect or Irrelevant (Bond) Angle(s) in CIF ...      28.45 Deg.
              C4   -C1   -C3      1_555   1_555   1_555 ......     #   13 Check
PLAT779_ALERT_4_G Suspect or Irrelevant (Bond) Angle(s) in CIF ...      30.60 Deg.
              C4   -C2   -C3      1_555   1_555   1_555 ......     #   20 Check
PLAT779_ALERT_4_G Suspect or Irrelevant (Bond) Angle(s) in CIF ...      30.60 Deg.
              C4   -C2   -C3      2_655   1_555   2_655 ......     #   23 Check
PLAT779_ALERT_4_G Suspect or Irrelevant (Bond) Angle(s) in CIF ...      30.60 Deg.
              C2   -C4   -C3      1_555   1_555   1_555 ......     #   33 Check
PLAT779_ALERT_4_G Suspect or Irrelevant (Bond) Angle(s) in CIF ...      30.00 Deg.
              C4   -C4   -N5      2_655   1_555   1_555 ......     #   36 Check
PLAT811_ALERT_5_G No ADDSYM Analysis: Too Many Excluded Atoms ....          ! Info
```

---

15 **ALERT level A** = Most likely a serious problem - resolve or explain
11 **ALERT level B** = A potentially serious problem, consider carefully
9 **ALERT level C** = Check. Ensure it is not caused by an omission or oversight
46 **ALERT level G** = General information/check it is not something unexpected

5 ALERT type 1 CIF construction/syntax error, inconsistent or missing data
58 ALERT type 2 Indicator that the structure model may be wrong or deficient
4 ALERT type 3 Indicator that the structure quality may be low
12 ALERT type 4 Improvement, methodology, query or suggestion
2 ALERT type 5 Informative message, check

It is advisable to attempt to resolve as many as possible of the alerts in all categories. Often the minor alerts point to easily fixed oversights, errors and omissions in your CIF or refinement strategy, so attention to these fine details can be worthwhile. In order to resolve some of the more serious problems it may be necessary to carry out additional measurements or structure refinements. However, the purpose of your study may justify the reported deviations and the more serious of these should normally be commented upon in the discussion or experimental section of a paper or in the "special_details" fields of the CIF. checkCIF was carefully designed to identify outliers and unusual parameters, but every test has its limitations and alerts that are not important in a particular case may appear. Conversely, the absence of alerts does not guarantee there are no aspects of the results needing attention. It is up to the individual to critically assess their own results and, if necessary, seek expert advice.

Publication of your CIF in IUCr journals

A basic structural check has been run on your CIF. These basic checks will be run on all CIFs submitted for publication in IUCr journals (*Acta Crystallographica*, *Journal of Applied Crystallography*, *Journal of Synchrotron Radiation*); however, if you intend to submit to *Acta Crystallographica Section C* or *E* or *IUCrData*, you should make sure that full publication checks are run on the final version of your CIF prior to submission.

Publication of your CIF in other journals

Please refer to the *Notes for Authors* of the relevant journal for any special instructions relating to CIF submission.

PLATON version of 12/09/2022; check.def file version of 09/08/2022

Datablock MFM-305-NO2 - ellipsoid plot

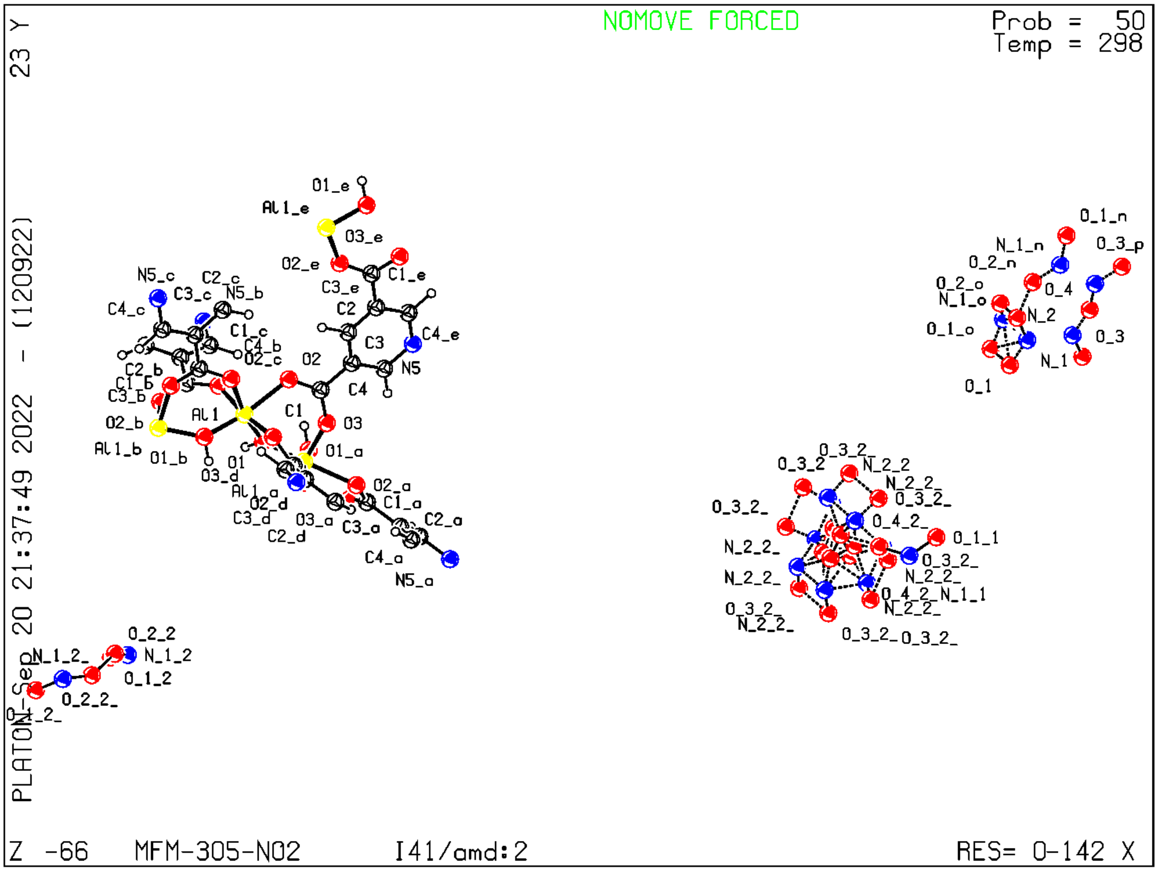

Supplement: Supplementary file 3 — Supporting Information [file ANIE-62-0-s004.pdf]
