# Supplementary material for: Modulation of Uptake and Reactivity of Nitrogen Dioxide in Metal‐Organic Framework Materials
Source: Angew Chem Int Ed Engl. 2023 Jun 2;62(28):e202302602. doi: 10.1002/anie.202302602 (PMC10962595; doi:10.1002/anie.202302602)
Supplement: Supplementary file 5 — Supporting Information [file ANIE-62-0-s003.pdf]

Angewandte Chemie
Eine Zeitschrift der Gesellschaft Deutscher Chemiker

Supporting Information

Modulation of Uptake and Reactivity of Nitrogen Dioxide in Metal-Organic Framework Materials

Z. Wang, A. M. Sheveleva, D. Lee, Y. Chen, D. Iuga, W. T. Franks, Y. Ma, J. Li, L. Li, Y. Cheng, L. L. Daemen, S. J. Days, A. J. Ramirez-Cuesta, B. Han, A. S. Eggeman, E. J. L. McInnes, F. Tuna*, S. Yang*, M. Schröder**

Table of Contents

1. Experimental Section 3

1.1 Synthesis of MFM-305-CH_3, MFM-305-CD_3 and MFM-305 3

1.2 NO_2 safety 3

1.3 Powder X-ray diffraction 3

1.4 TEM experiments 3

1.5 Breakthrough experiments 3

1.6 Powder X-ray diffraction data collection and Rietveld refinement 3

1.7 Inelastic neutron scattering (INS) and DFT calculations for INS spectroscopy 4

1.8 DFT modelling and simulation 5

1.10 Electron paramagnetic resonance (EPR) spectroscopy 6

2. PXRD patterns for MFM-305 materials and NO_2 stability tests 8

3. Views of structures 11

4. Structure refinement data 14

5. Elemental analysis 16

6. Solid-state NMR spectra 18

7. INS data and analysis 22

8. EPR data and analysis 26

References 48

1. Experimental Section

1.1 Synthesis of MFM-305-CH_3, MFM-305-CD_3 and MFM-305.

All reagents were used as received from commercial suppliers without further purification. Synthesis and activation of MFM-305-CH_3, MFM-305-CD_3 and MFM-305 were carried out using our previously reported method.[1]

1.2 NO_2 safety

All systems involved in the supply, delivery and measurement of NO_2 were tested rigorously for leaks and were used within the range of a NO_2 detection system with a sensitivity of 0.1 ppm.

1.3 Powder X-ray diffraction

Powder X-ray diffraction (PXRD) patterns were collected using PANalytical X'Pert Pro MPD diffractometer in Bragg-Brentano geometry using Cu-Kα1 radiation (λ = 1.5406 Å).

1.4 TEM experiments

Scanning-transmission electron microscopy (STEM) images and energy dispersive X-ray spectroscopy (EDX) elemental maps were collected using a Thermo Fisher Talos STEM (G2 20-200) equipped with high-angle annual dark-field (HAADF) detector and ChemiSTEM Super-X EDX detector, operating at 200 kV. TEM samples were prepared by dispersing the powders in EtOH and drop-cast onto a copper grid coated with an amorphous lacey carbon film.

1.5 Breakthrough experiments

In order to measure the NO_2 adsorption of the materials, dynamic breakthrough experiments were performed at 298 K. The MOF samples were activated at 373 K under dynamic vacuum for 16 h before breakthrough experiments, and 100 mg of desolvated sample was packed into a fixed-bed reactor. The sample was then heated at 373 K under He flow for 2 h to achieve further activation. The fixed-bed was cooled to 298 K and the breakthrough experiment performed with a stream of 500 ppm NO_2 diluted in He. The flow rate of the gas mixture was 80 mL min^{-1}. A Matrix MG5 FTIR spectrometer was used to analyse the outlet gases for detection of NO_2 and NO. The gas concentration, C, of NO_2 at the outlet was compared with the corresponding inlet concentration C_0, where $C/C_0 = 1$ indicates complete breakthrough.[2]

1.6 Powder X-ray diffraction data collection and Rietveld refinement

High-resolution X-ray powder diffraction data of activated, and NO_2-loaded MFM-305-CH_3 and MFM-305 was measured on beamline I11 in the Diamond Light Source. A high brightness monochromatic beam was produced by a Si(111) monochromator and double-bounce harmonic rejection mirrors. The samples were ground to provide a uniform particle size, packed into a 0.7 mm borosilicate capillary and mounted into a gas cell for gas dosing. The sample was activated under vacuum (1×10^{-6} mbar) at 353 K (MFM-305-CH_3) and

393 K (MFM-305) for > 10 h to remove residual solvent molecules from the material. Diffraction data for the activated sample were collected and analysed to confirm that no residual solvent molecules are present in the pores. NO_2-loaded MFM-305-CH_3 and MFM-305 was prepared in house lab. Data were collected between 0 and 150° under the wavelength of 0.826844 Å using a step size of 0.001° with five multi-analysing crystal (MAC) detectors.

TOPAS 5[3] was used to perform Pawley and Rietveld refinement. Background, cell parameters and peak profile were first refined by Pawley refinement and then transferred to Rietveld refinement. Stephen fitting[4] was applied to describe the diffraction peaks and their anisotropic broadening. With initial atom positions from the published structure, the SXPD patterns were refined by the Rietveld method. The refined structural parameters include the fractional coordinates (x, y, z) and isotropic displacement factors for all atoms, and the site occupancy factors (SOF) for guest molecules. The final stage of Rietveld refinement involved soft restraints to the C–C bond lengths within the benzene rings. Rigid body refinement was applied to the guest molecules in the pore. The quality of the Rietveld refinements was assured with low weighted profile factors and well-fitted patterns with reasonable isotropic displacement factors within experimental errors.

1.7 Inelastic neutron scattering (INS) and DFT calculations for INS spectroscopy

INS spectra were recorded on the VISION spectrometer at Spallation Neutron Source, Oak Ridge National Laboratory (USA). VISION is an indirect geometry crystal analyser instrument that provides a wide dynamic range with high resolution. The sample of desolvated MFM-305-CH_3 and MFM-305 were loaded into a cylindrical vanadium sample container with an indium vacuum seal and connected to a gas handling system respectively. The samples were degassed at 10^{-7} mbar at 80 and 120 °C for 1 day to remove any remaining trace guest water molecules. The temperature during data collection was controlled using a closed cycle refrigerator (CCR) cryostat (10 ± 0.1 K). The loading of NO_2 was performed volumetrically at room temperature to ensure that NO_2 was present in the gas phase when not adsorbed and also to ensure sufficient mobility of NO_2 inside the crystalline structure. The temperature was then reduced to below 10 K in order to perform the scattering measurements with the minimum achievable thermal motion for the framework host and adsorbed NO_2 and N_2O_4 molecules. Background spectra [sample can plus bare MFM-305-CH_3/MFM-305] were subtracted to obtain the difference spectra.

INS was used to study the binding interaction and structure dynamics in this case because it has several unique advantages:

• INS spectroscopy is ultra-sensitive to the vibrations of hydrogen atoms, and hydrogen is ten times more visible than other elements due to its high neutron cross-section.

• The technique is not subject to any optical selection rules. All vibrations are active and, in principle, measurable.

• INS observations are not restricted to the centre of the Brillouin zone (gamma point) as is the case for optical techniques.

• INS spectra can be readily and accurately modelled: the intensities are proportional to the concentration of elements in the sample and their cross-sections, and the measured INS intensities relate straightforwardly to the associated displacements of the scattering atom. Treatment of background correction is also straightforward.

• Neutrons penetrate deeply into materials and pass readily through the walls of metal containers making neutrons ideal to measure bulk properties of this material.

• INS spectrometers cover the whole range of the molecular vibrational spectrum, 0-500 meV (0-4000 cm^{-1})

• INS data can be collected at below 10 K, where the thermal motion of the MOF material and adsorbed NO_2, and N_2O_4 molecules can be significantly reduced.

• Calculation of the INS spectra by DFT vibrational analysis can be readily achieved, and DFT calculations relate directly to the INS spectra, and, in the case of solid state calculations, there are no approximations other than the use of DFT eigenvectors and eigenvalues to determine the spectral intensities.

1.8 DFT modelling and simulation

Vibrational frequencies and polarization vectors were calculated using CP2K (http://www.cp2k.org)[5] based on the mixed Gaussian and plane-wave scheme[6] and the Quickstep module[7]. The calculation used molecularly optimized Double-Zeta-Valence plus Polarization (DZVP) basis set[8], Goedecker-Teter-Hutter pseudopotentials[9], and the Perdew-Burke-Ernzerhof (PBE) exchange correlation functional[10]. The plane-wave energy cutoff was 400 Ry. The DFT-D3 level correction for dispersion interactions, as implemented by Grimme et al[11], was applied with a cutoff distance of 15 Å. Structural optimization was performed using the Broyden-Fletcher-Goldfarb-Shannon (BFGS) optimizer, until the maximum force is below 0.00045 Ry/Bohr (0.011 eV/Å). The finite displacement method was used for the phonon calculation with incremental displacement of 0.01 Bohr (0.0053 Å). The INS spectrum was then simulated using the OClimax software[12].

1.9 Solid state NMR spectroscopy

Solid-state (ss) NMR spectra were recorded using two regimes. Moderate-field experiments employed a Bruker 9.4 T (400 MHz ^{1}H Larmor frequency) AVANCE III spectrometer equipped with a 4 mm HFX MAS probe. Experiments were acquired at ambient temperature using a magic angle spinning (MAS) frequency of 12 kHz. ^{1}H-pulses of 100 kHz were used and ^{13}C spin-locking at ~50 kHz was applied for 2 ms (1D) and 0.25 ms (2D) with corresponding ramped (70-100 %) ^{1}H spin-locking at ~75 kHz for 1D {^{1}H-}^{13}C CP and 2D ^{1}H-^{13}C heteronuclear dipolar correlation (HETCOR) experiments. ^{27}Al-pulses of 70 kHz and 0.5 μs duration were used for quantitative 1D ^{27}Al NMR spectra and ^{27}Al spin-locking at ~7 kHz was applied for 0.25 ms, with corresponding square ^{1}H spin-locking at ~33 kHz, for 2D ^{1}H-^{27}Al HETCOR experiments. ^{15}N spin-locking at ~31 kHz was applied for 3 ms with corresponding ramped (70-100 %) ^{1}H spin-locking at ~52 kHz for 1D {^{1}H-}^{15}N CP experiments. 100 kHz of SPINAL-64[13] heteronuclear ^{1}H decoupling was used throughout signal acquisition for all experiments. Samples were treated and packed into 4 mm o.d. zirconia rotors under inert conditions and sealed with a Kel-F rotor cap. For NO_2 adsorption, the rotor packed with MFM-305-CH_3 was

opened under inert conditions and the sample remained in this container during dosing with NO_2, before subsequent resealing with the Kel-F cap. The magnetic field was set (indirectly) for the ^{13}C chemical shifts to be relative to TMS (= 0 ppm).

High-field NMR spectra, recorded at the UK High-Field Solid-State NMR Facility, employed a Bruker 23.5 T (1000 MHz ^{1}H Larmor frequency) AVANCE NEO spectrometer equipped with a 1.3 mm HX MAS probe. Experiments were acquired at ambient temperature using a MAS frequency of 60 kHz. ^{1}H-pulses of 100 kHz were used, except during the S_2 recoupling sequence [14],[15] of the ^{1}H homonuclear DQ-SQ dipolar correlation experiments where 30 kHz pulses were required and during the $SR4^{2}_{1}$ recoupling sequence[16] of the PT-D-HMQC ^{35}Cl-^{1}H [17] and SPI-R^3 recoupling sequence[18] of the D-HMQC ^{14}N-^{1}H[19] dipolar correlation MAS NMR experiments where 120 kHz pulses were used. WURST[20],[21] pulses of 5 kHz (central transition) nutation frequency were applied to the ^{35}Cl nuclei during the $SR4^{2}_{1}$ heteronuclear recoupling. One full loop of S_2 recoupling was used to reintroduce the homonuclear dipolar interaction between ^{1}H-spins during both excitation and reconversion periods, giving a total mixing time of 133 μs. Four full loops of $SR4^{2}_{1}$ recoupling were used to reintroduce heteronuclear dipolar interactions between ^{1}H and ^{35}Cl spins during both excitation and reconversion periods, giving a total mixing time of 800 μs. The ^{35}Cl central transition was excited with a 2 μs pulse at a central transition nutation frequency of 120 kHz. ^{35}Cl MAS NMR spectra were acquired using an echo pulse sequence preceded by a WURST saturation. 16384 transients were co-added with a repetition delay of 0.5 s. 16 rotor periods were used for 8 SPI-R^3 (x, -x) pulses (1 pulse per rotor period) to reintroduce heteronuclear dipolar interactions between ^{1}H and ^{14}N spins during both excitation and reconversion periods, giving a total mixing time of 533 μs. The ^{14}N transitions were excited with a 6 μs pulse with RF amplitude of ~50 kHz. For the 2D ^{1}H homonuclear DQ-SQ dipolar correlation experiment, 16 transients were acquired for each of 82 complex (STATES-TPPI) rotor-synchronised t_1 increments (1 rotor period). For the 2D ^{35}Cl-^{1}H dipolar correlation experiments, 128 or 2048 (for the NO_2-dosed sample) transients were acquired for each of 32 complex (STATES) rotor-synchronised t_1 increments (1 rotor period). For the 2D ^{14}N-^{1}H dipolar correlation experiments, 400 transients were acquired for each of 29 complex (STATES-TPPI) rotor-synchronised t_1 increments (1 rotor period). Samples were treated and packed into 1.3 mm o.d. zirconia rotors under inert conditions and sealed with a Vespel rotor cap. The magnetic field was set (indirectly) for the ^{1}H chemical shifts to be relative to TMS (= 0 ppm).

1.10 Electron paramagnetic resonance (EPR) spectroscopy

Low temperature CW EPR spectra (10 K) were carried out at X-band (*ca.* 9.5 GHz) on a Bruker EMX 300 EPR spectrometer equipped with a liquid He cryostat. A modulation amplitude of 1 mT was used with microwave power of ~ 2.0 mW after testing for saturation of spectral lines. Field corrections were applied by measuring relevant EPR standards (Bruker Strong Pitch). For accuracy, the tube size and tube position in the cavity were kept constant. Pulsed electron paramagnetic resonance (EPR) measurements were detected at X-band (ca. 9.7 GHz) on a Bruker Elexsys E580 spectrometer. The microwave frequency was measured with a built-in digital counter and the magnetic field was calibrated using a Bruker strong pitch reference sample.

Echo-detected field swept (EDFS) spectra were measured at X-band using the pulse sequence ($\pi/2 - \tau - \pi - \tau$ – echo) with $\pi/2$ and π pulse lengths of 200 and 400 ns, respectively. The interpulse delay τ was 200 ns. Electron-nuclear double resonance (ENDOR) measurements used the Davies sequence (π_{inv} – RF –$\pi/2 - \tau - \pi - \tau$ – echo) with microwave inversion and radiofrequency (RF) π-pulse durations of 200 and 1200 ns, respectively. HYSCORE spectra were measured at X-band using standard pulse sequence ($\pi/2 - \tau - \pi/2$ – T1 – π – T2 – $\pi/2 - \tau$ – echo); the length of mw pulses was $\pi/2$ = 16 ns and π = 26 ns. The time delay between first two pulses was taken as τ = 200 ns, and starting values of T1 and T2 incrementing times were 100 ns. The (128x128) HYSCORE data array was recorded with a time increment of 16 ns, and then two-dimensional Fourier transform (FT) magnitude spectra were calculated. Simulation of the EPR spectra was performed with the EasySpin/MATLAB toolbox, which employs the exact diagonalization of the spin Hamiltonian matrix.[22]

EPR samples were prepared *via in situ* activation and gas-loading process, and the material was transferred into a J. Young X-band EPR tube (4 and o.d.). The samples were then evacuated at 10^{-5} mbar for 2 h at room temperature, and activated under dynamic vacuum for 12 h at T = 373 K (MFM-305-CH_3 and MFM-305-CD_3) and T = 393 K (MFM-305). The activated sample was placed into a J. Young EPR quartz tube (4 mm o.d.) equipped with a vacuum valve connected to a NO_2 cylinder equipped with a pressure regulator. The whole system was checked for leakage and the MOF sample was left for 1 h at 1 bar pressure under NO_2. After gas adsorption was complete the tube was evacuated at 10^{-2} mbar for 1 min, sealed and disconnected from the system at low temperature. The NO_2 gas was condensed into the EPR tube using a cold trap method where the sample volumes containing the MOF material were cooled at 77 K. The application of the cold trap method during the sealing of the quartz glass tubes ensured that the entire amount of loaded NO_2 was fully trapped within the EPR tubes. Loading of NO_2 was rigorously leak tested and used only within range of a NO_2 detection system with a sensitivity of 0.1 ppm.

2. PXRD patterns for MFM-305 materials and NO_2 stability tests

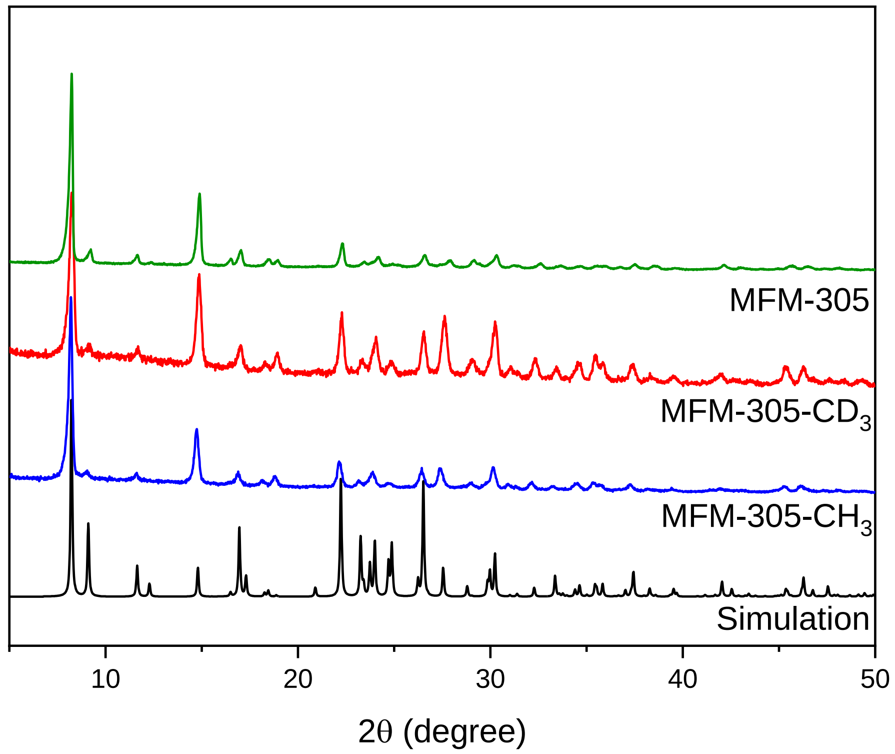

Figure S1. PXRD patterns of as-prepared MFM-305-CH_3, MFM-305 and MFM-305-CD_3.

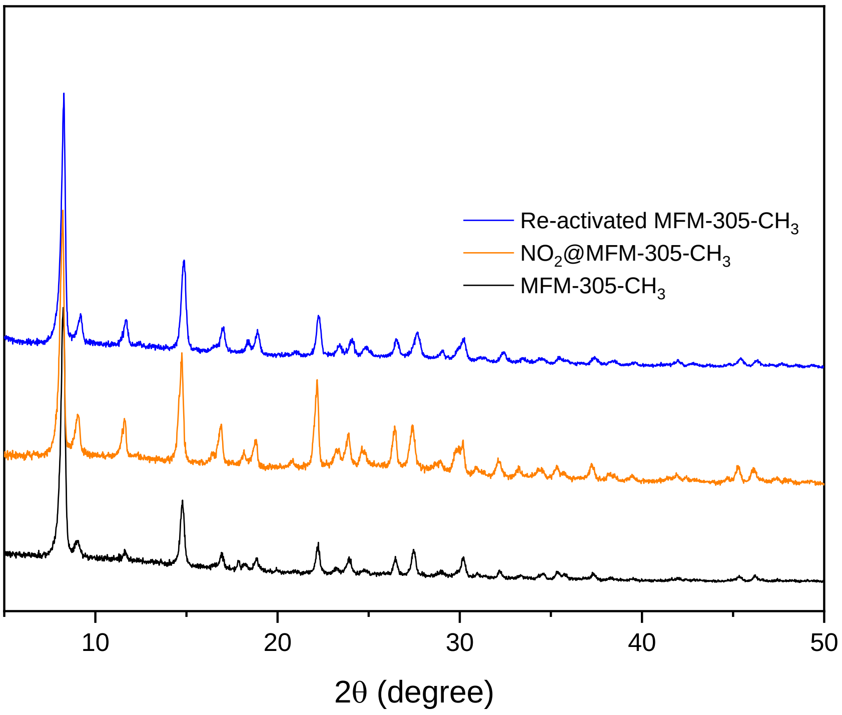

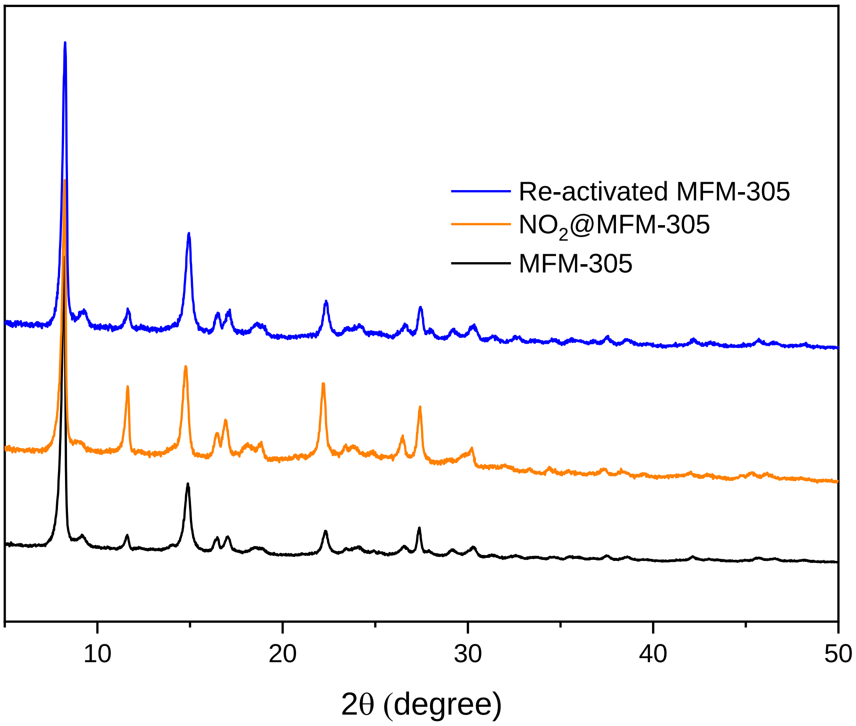

Figure S2. PXRD patterns of MFM-305-CH_3 (upper) and MFM-305 (lower) after adsorption of NO_2 and after reactivation.

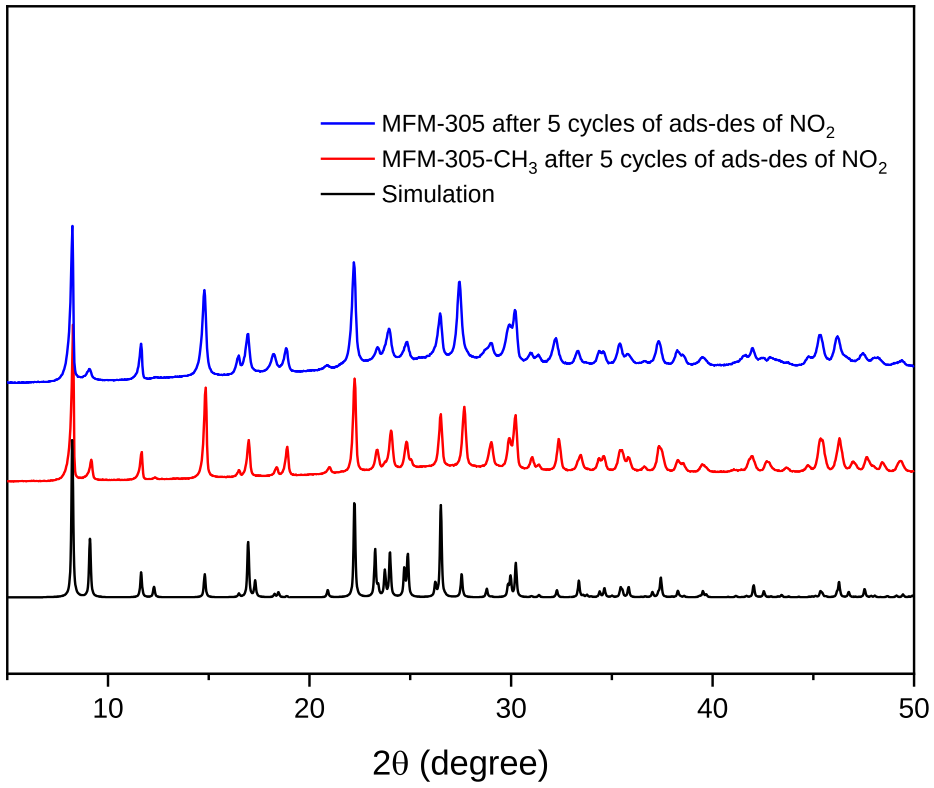

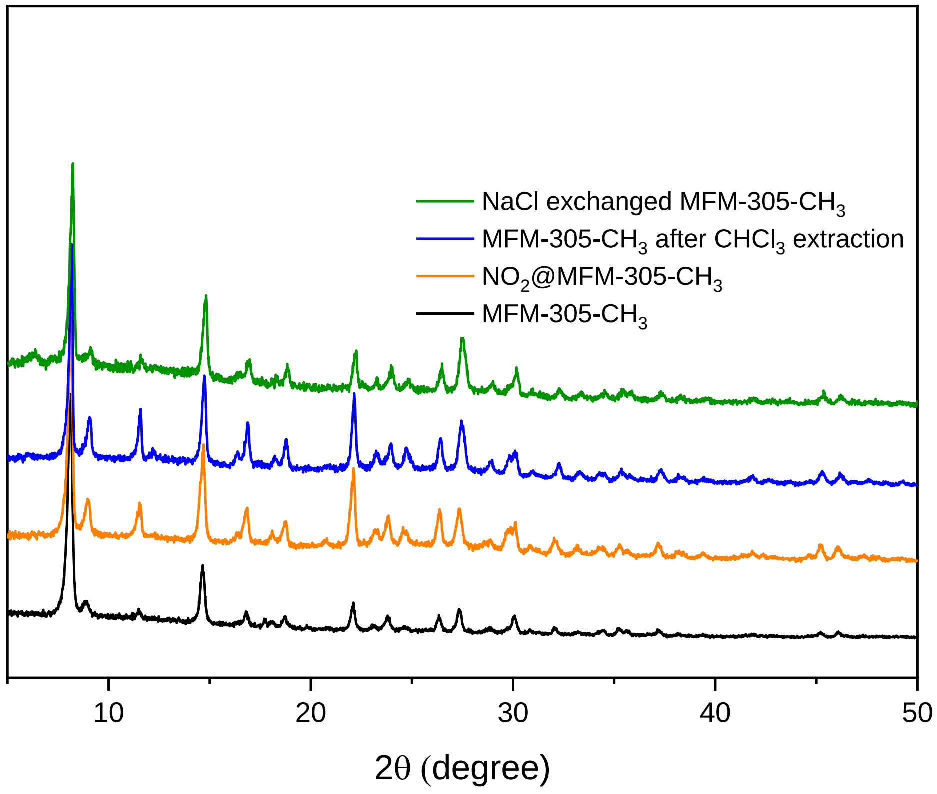

Figure S3. PXRD patterns of MFM-305-CH_3 and MFM-305 after 5 cycles of adsorption and desorption of NO_2 (upper); PXRD patterns of MFM-305-CH_3 before and after adsorption of NO_2, after extraction with $CHCl_3$, and after ion-exchange with a solution of NaCl in MeOH (lower).

3. Views of structures

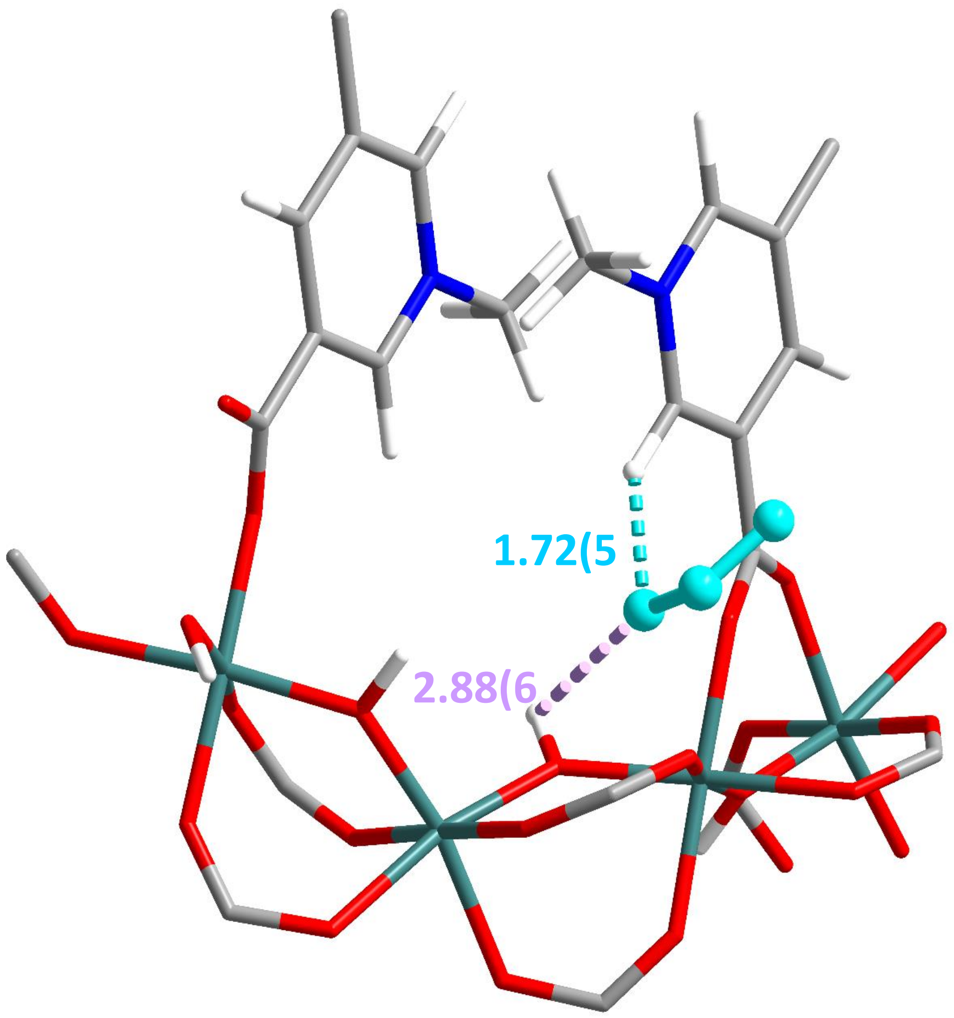

Figure S4. View of the binding site of NO_2 in MFM-305-CH_3 (atom colour scheme as in Figure 1).

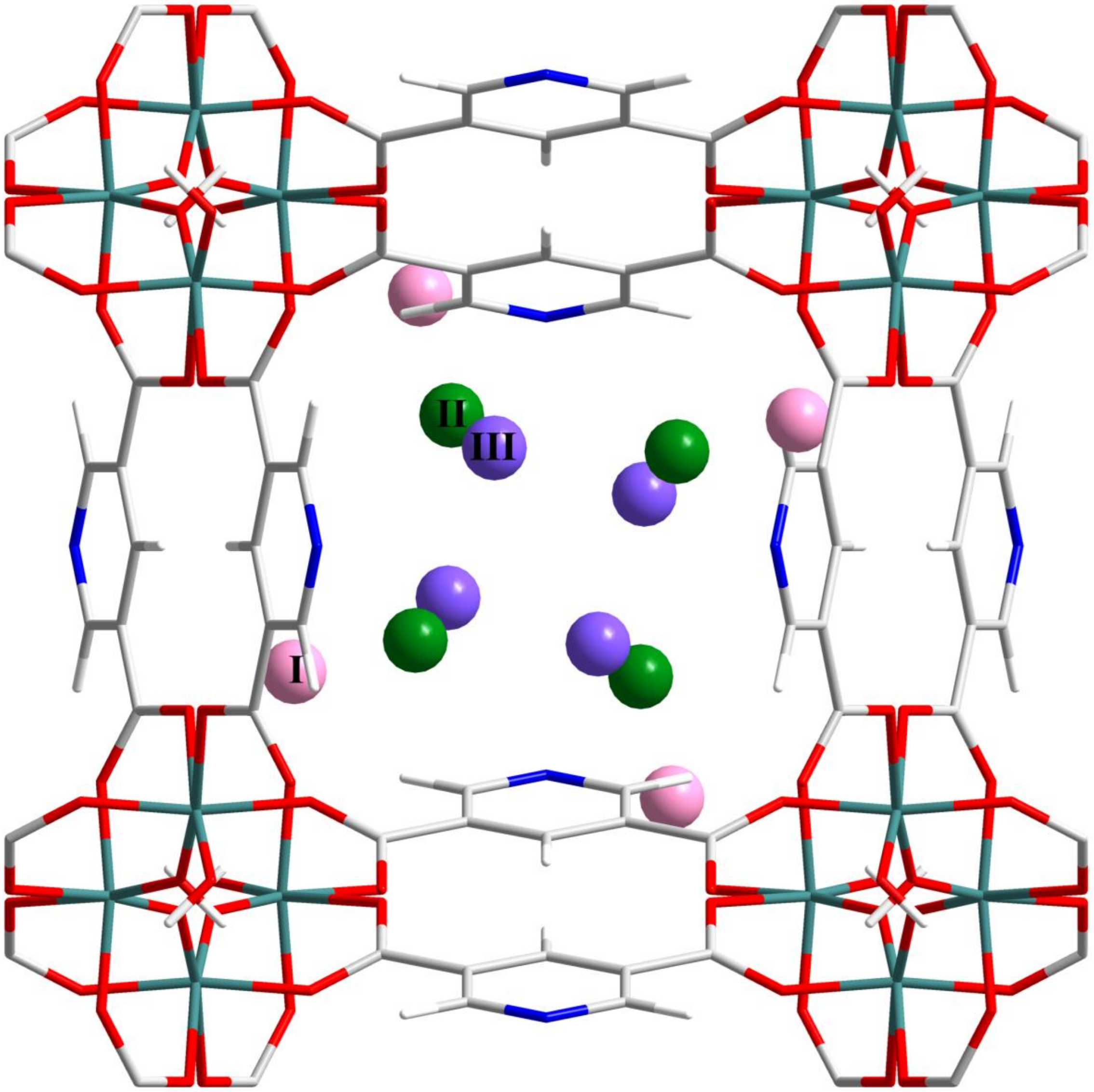

Figure S5. View of binding sites of NO_2 in MFM-305 (atom colour scheme as in Figure 1; pink: NO_2 at site I; green: NO_2 at site II; purple: NO_2 at site III).

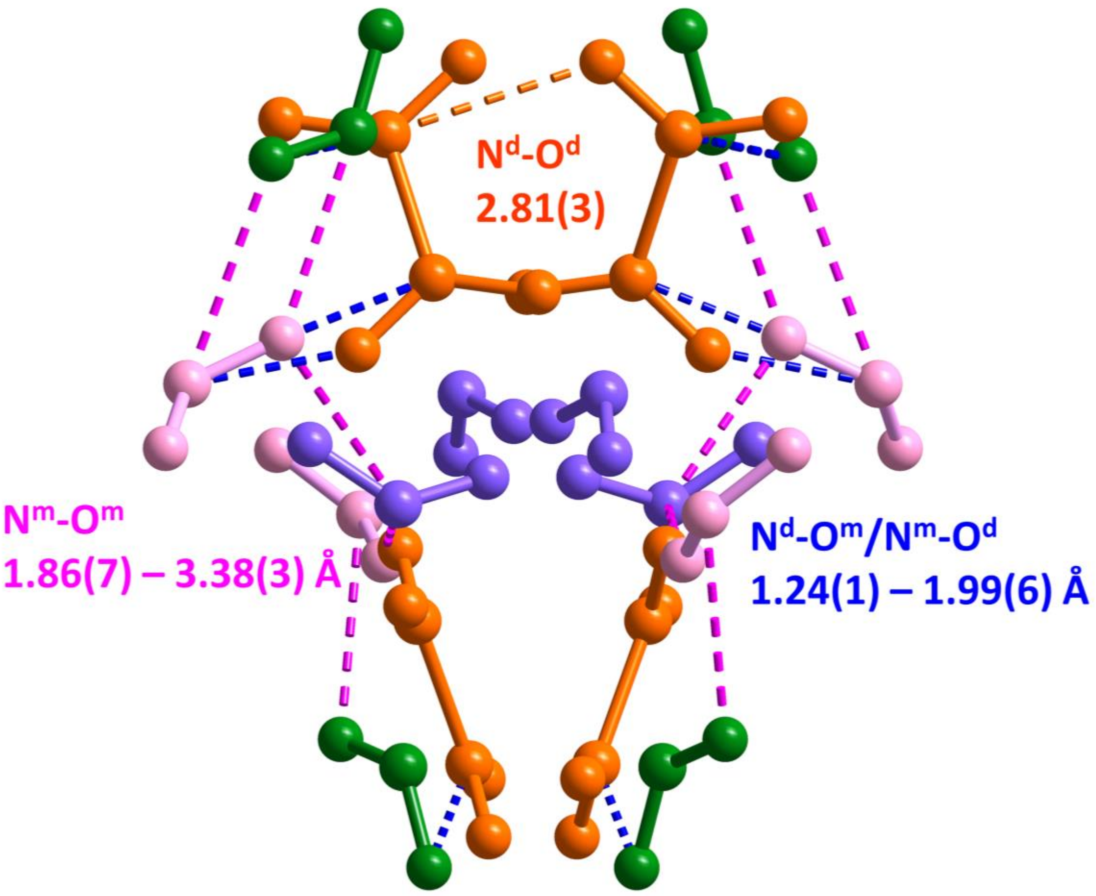

Figure S6. Packing of NO_2-N_2O_4 in the pore of MFM-305 (key dipole-dipole interactions are labelled) ('d': N_2O_4 dimer; 'm':NO_2 monomer).

4. Structure refinement data

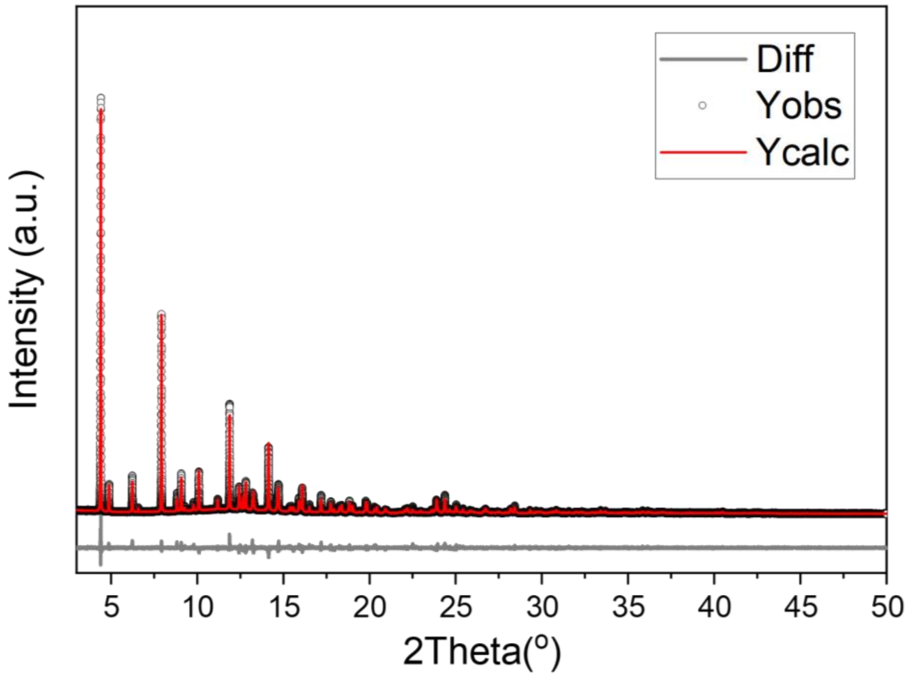

Figure S7a. Experimental data (black), Rietveld refinement (red) and the difference (grey) for SPXRD patterns of NO_2-loaded MFM-305-CH_3 at room temperature at a 2θ range of 3 - 50° [λ = 0.826844 Å].

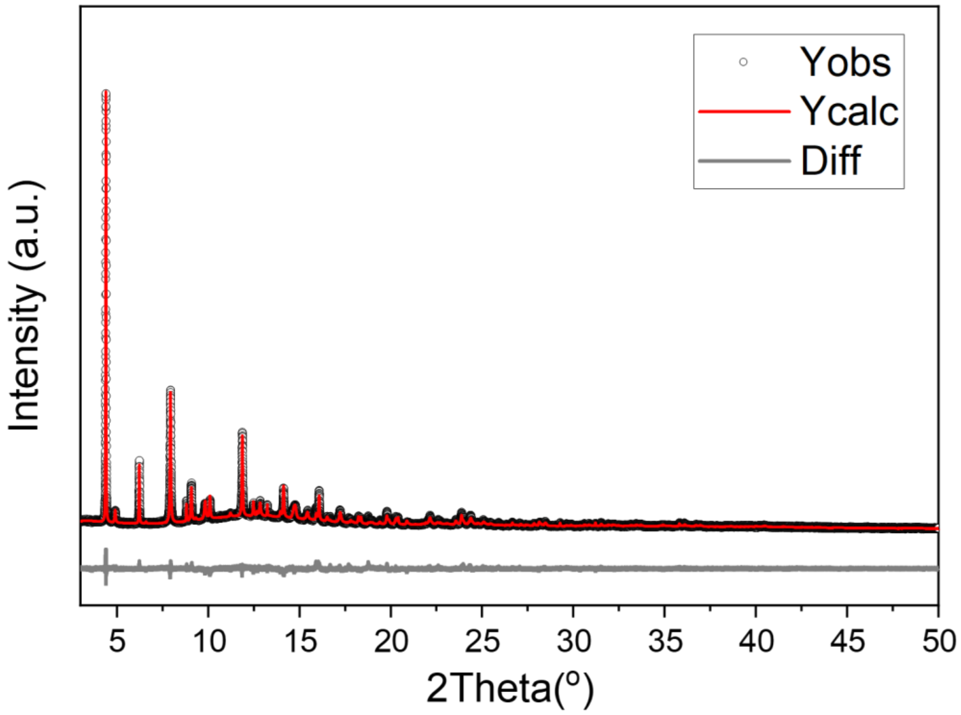

Figure S7b. Experimental data (black), Rietveld refinement (red) and the difference (grey) for SPXRD patterns of NO_2-loaded MFM-305 at room temperature at a 2θ range of 3 - 50° [λ = 0.826844 Å].

Table S1. Details of crystal data for gas-loaded MFM-305 and MFM-305-Me determined by synchrotron X-ray powder diffraction.

Materials	NO_2@MFM-305	NO_2@MFM-305-CH_3
Formula	$Al(OH)(C_7H_3NO_4)$ $(NO_2)_{3.15}\cdot(N_2O_4)_{0.175}$	$Al(OH)(C_8H_6NO_4)Cl_{0.78}$ $(NOCl)_{0.22}\cdot(NO_3^-)_{0.22}\cdot(NO_2)_{0.5}$
Crystal System	Tetragonal	Tetragonal
Space group	$I4_1/amd$	$I4_1/amd$
a [Å]	21.5132(6)	21.4771(3)
c [Å]	10.7854(3)	10.84984(17)
V [Å^3]	4991.6(3)	5004.64(17)
Radiation type	Synchrotron X-ray λ = 0.826844 Å	
Scan method	Capillary mode, MAC detector mode	
R_{wp}(%)	8.1187	9.4745
R_p(%)	5.9689	6.9942
R_{bragg}(%)	4.3515	5.7194
R_{exp}(%)	3.9599	3.7832
GOF	2.0502	2.5043
CCDC	2209046	2209047

5. Elemental analysis

Table S2. Elemental composition of MFM-305-CH_3 materials obtained by means of CHN and halide analysis.

			**C**	**H**	**N**	**Cl**
As-synthesized MFM-305-CH_3	$Al(OH)(C_8H_6NO_4)Cl{\cdot}3H_2O$	Expected (%)	30.6	4.18	4.47	11.3
		Found (%)	31.9	4.24	4.60	10.2
Re-activated MFM-305-CH_3 (after NO_2 adsorption)	$Al(OH)(C_8H_6NO_4)\ {\cdot}NO_3$	Expected (%)	33.6	2.1	9.8	/
		Found (%)	33.8	3.27	7.84	6.15
NaCl-exchanged MFM-305-CH_3	$Al(OH)(C_8H_6NO_4)Cl{\cdot}3H_2O$	Expected (%)	30.6	4.18	4.47	11.3
		Found (%)	31.2	3.86	4.97	9.72

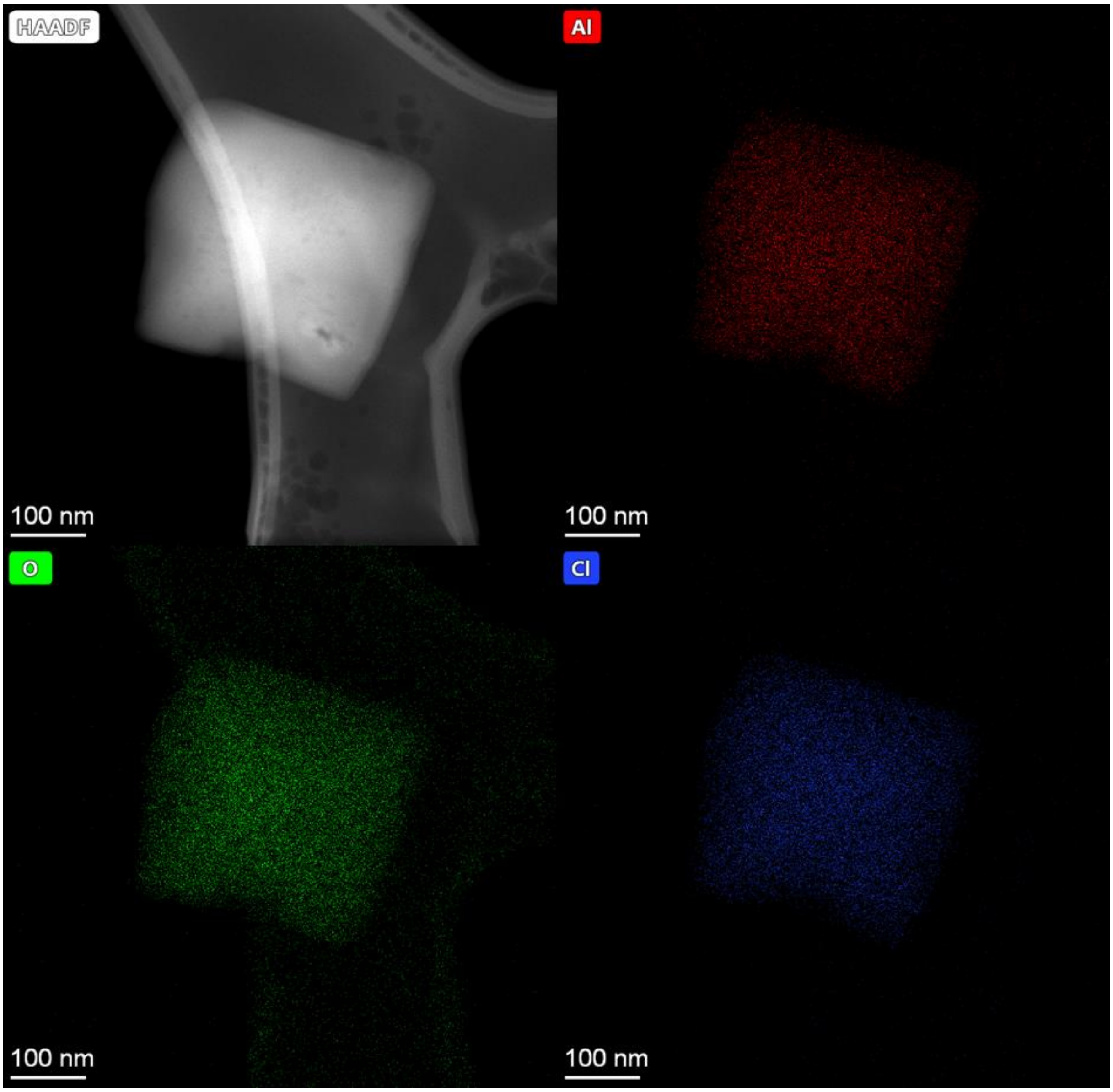

Figure 8. High resolution HAADF-STEM image and EDX mapping of as-synthesised MFM-305-CH_3.

6. Solid-state NMR spectra

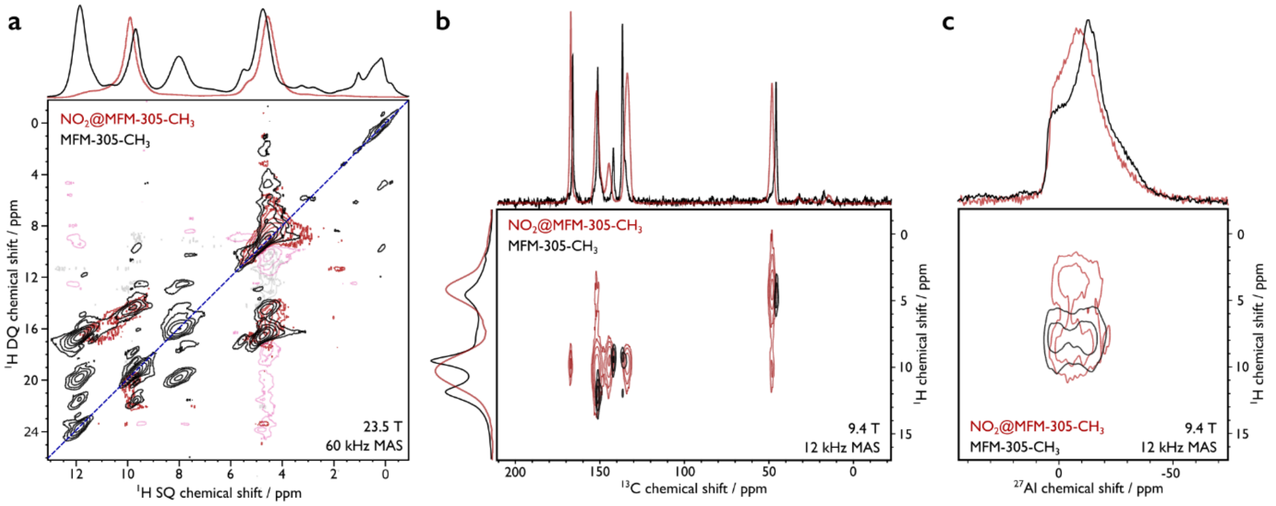

Figure S9. MAS NMR spectroscopy. (a) 2D ^{1}H homonuclear DQ-SQ dipolar correlation spectra of MFM-305-CH_3 (black) and NO_2@MFM-305-CH_3 (red), with corresponding ^{1}H NMR spectra (top), recorded at 23.5 T using a MAS frequency of 60 kHz; (b) 2D ^{1}H–^{13}C dipolar correlation (HETCOR) spectra of MFM-305-CH_3 (black) and NO_2@MFM-305-CH_3 (red), with corresponding ^{1}H (left) and {^{1}H-}^{13}C CP (top) NMR spectra, recorded at 9.4 T using a MAS frequency of 12 kHz; (c) 2D ^{1}H–^{27}Al dipolar correlation (HETCOR) spectra of MFM-305-CH_3 (black) and NO_2@MFM-305-CH_3 (red), with corresponding ^{27}Al NMR spectra (top), recorded at 9.4 T using a MAS frequency of 12 kHz. All spectra were recorded at ambient temperature.

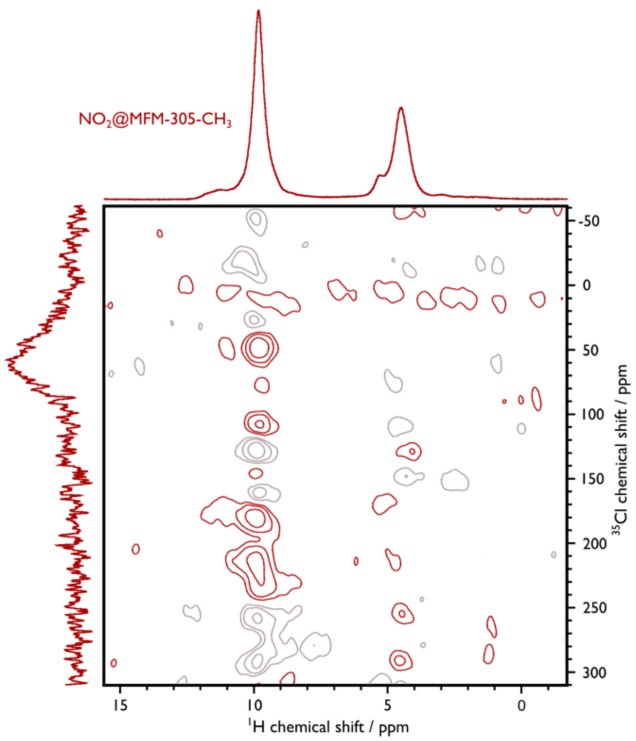

Figure S10. 23.5 T MAS NMR spectroscopy. 2D ^{35}Cl–^{1}H dipolar correlation spectrum of NO_2@MFM-305-CH_3 with corresponding ^{1}H (top) and ^{35}Cl (left) NMR spectra, all recorded at ambient temperature using a MAS frequency of 60 kHz. No correlation peaks are observed above the noise level. Positive and negative intensities are represented by red and grey contours, respectively.

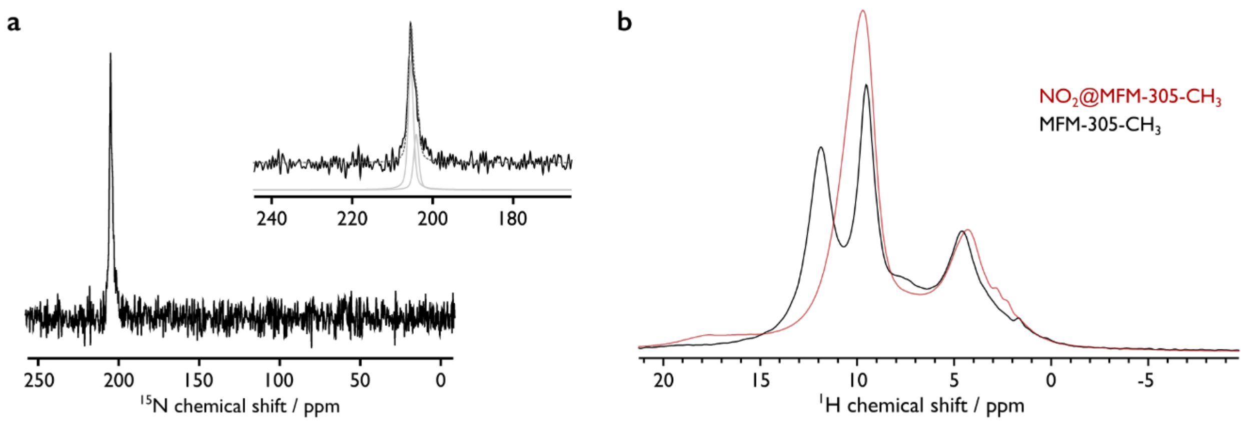

Figure S11. 9.4 T MAS NMR spectroscopy. (a) {^{1}H-}^{15}N CPMAS NMR spectrum of MFM-305-CH_3 and corresponding magnification including a fit to two Gaussian peaks (inset); (b) ^{1}H Hahn-echo NMR spectra of MFM-305-CH_3 (black) and NO_2@MFM-305-CH_3 (red). All spectra were recorded at ambient temperature using a MAS frequency of 12 kHz.

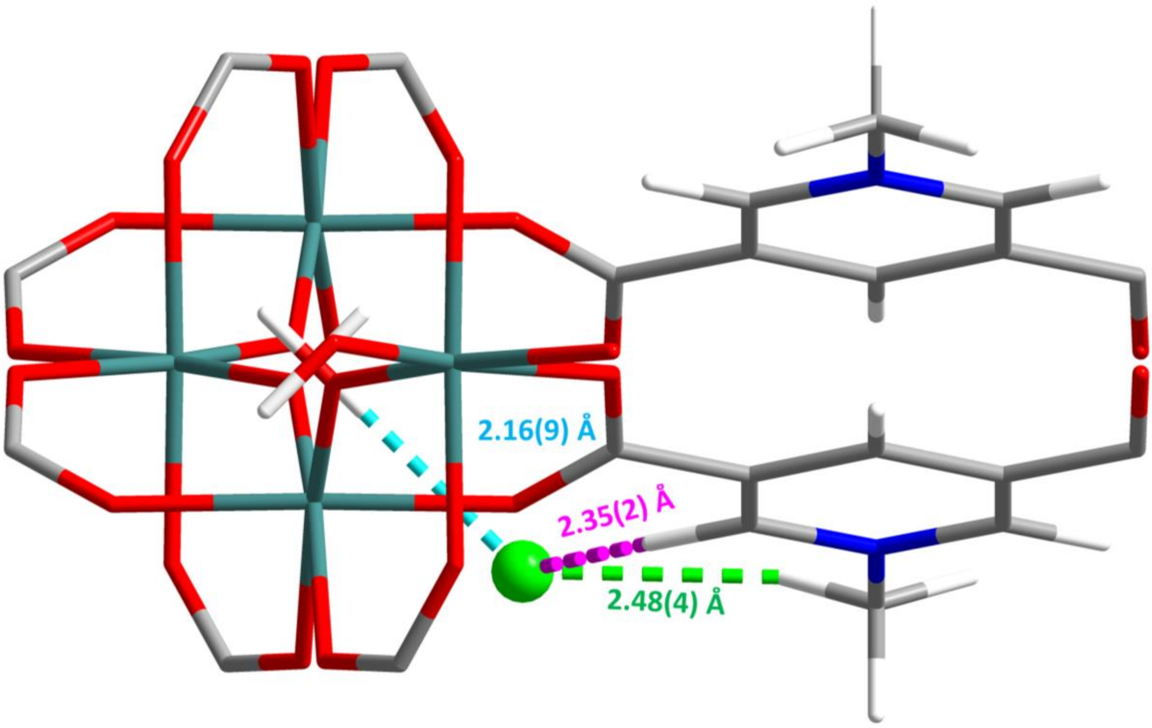

Figure S12. View of Cl…H hydrogen bonding interaction between Cl^- and framework protons in MFM-305-CH_3 (atom colour scheme as in Figure 1).

7. INS data and analysis

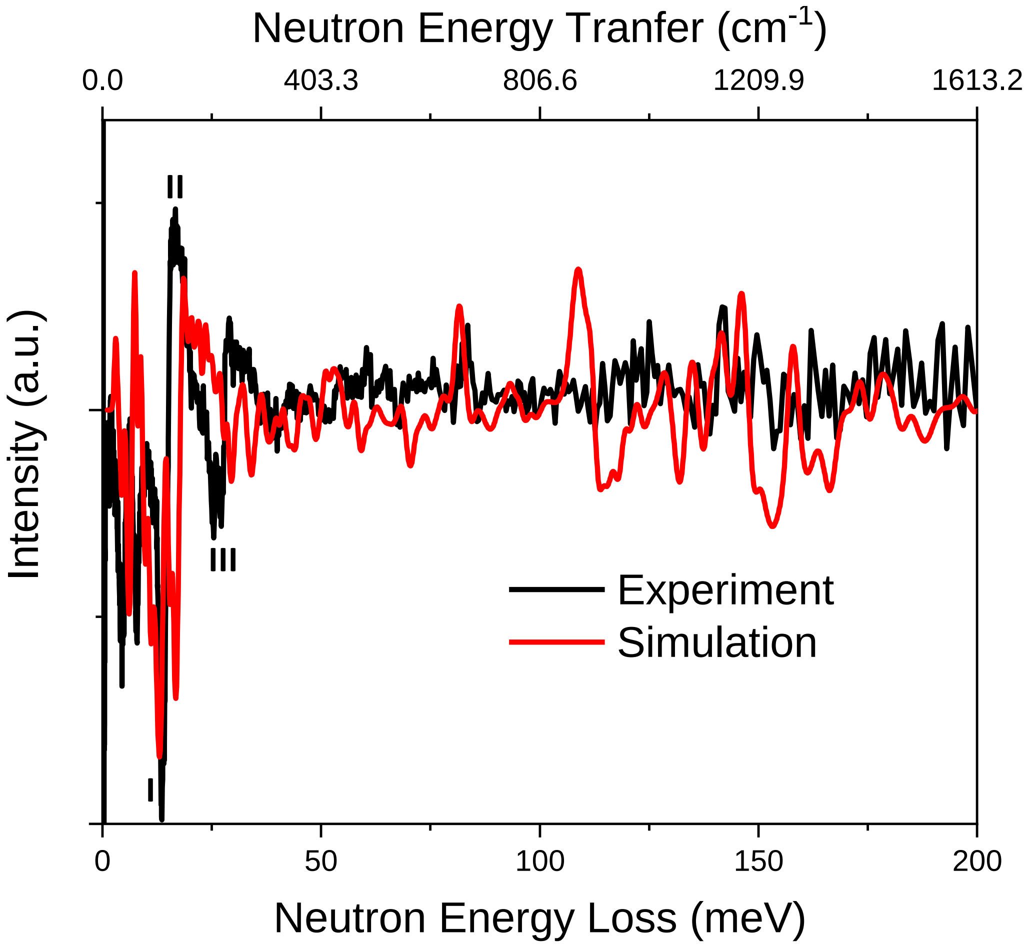

Figure S13. Comparison of the difference plots for experimental and DFT-calculated INS spectra of bare and NO_2-loaded MFM-305-CH_3.

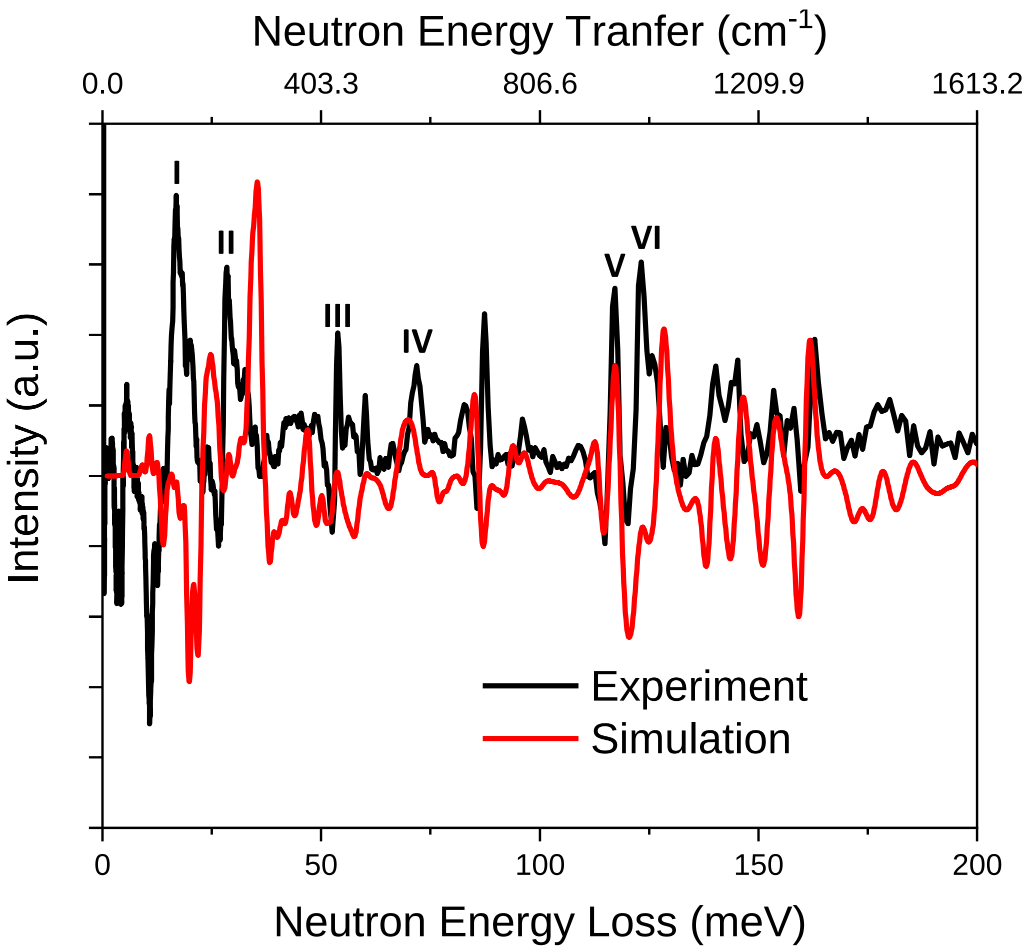

Figure S14. Comparison of the difference plots for experimental and DFT-calculated INS spectra of bare and NO_2-loaded MFM-305.

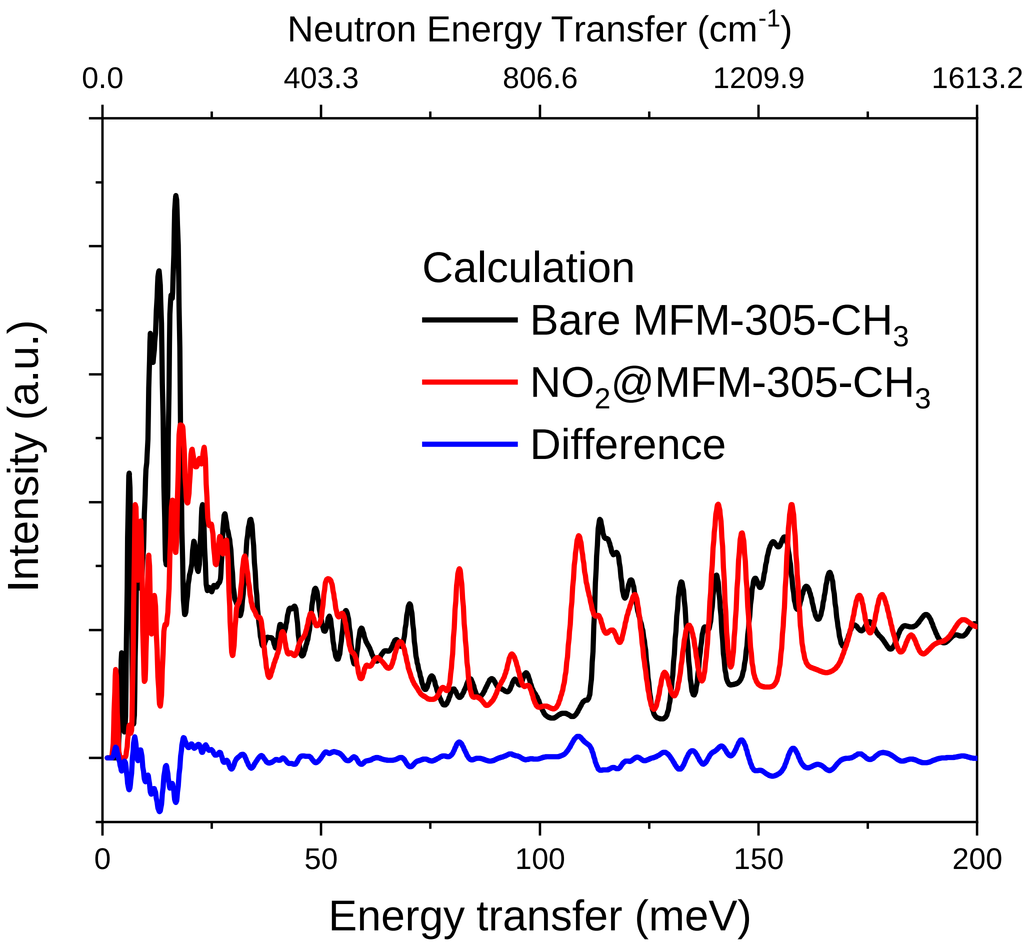

Figure S15. Comparison of the DFT-simulated INS spectra for bare and NO_2-loaded MFM-305-CH_3.

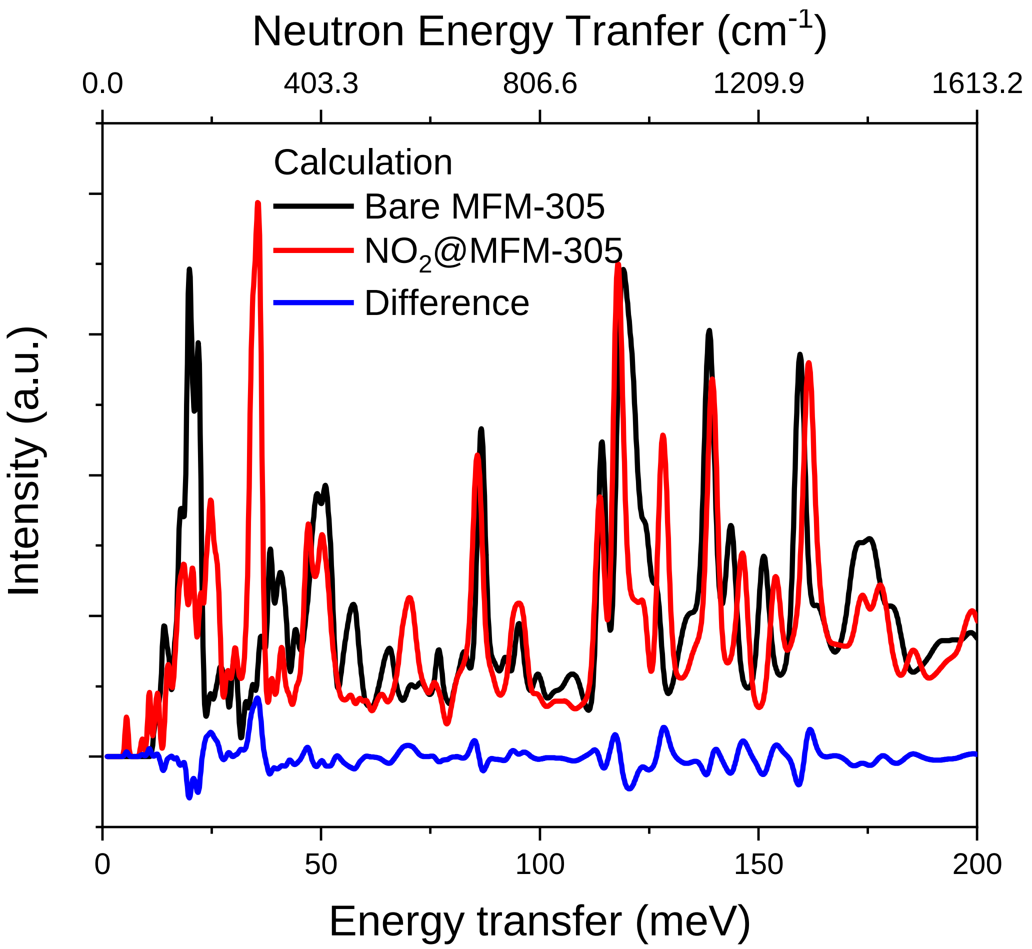

Figure S16. Comparison of the DFT-simulated INS spectra for bare and NO_2-loaded MFM-305.

8. EPR data and analysis

ENDOR and HYSCORE spectra show hyperfine interactions between the NO_2-based electron spin and ^1H nuclear spins of the MOF. The simulated model was calculated based on the purely dipolar (through space) interactions between the NO_2 and the nearest ^1H nuclei. The 3×3 dipolar ^1H hyperfine interaction matrices (A^{dip}) were calculated according to:

$$A^{\mathrm{dip}} = \frac{\mu_0}{4\pi h}\beta_e\beta_n \sum_k \rho_k \frac{3(\mathrm{g.n_k})(\mathrm{\tilde{n}_k}.g_n 1) - \mathrm{g}.g_n 1}{r_k^3}$$

Where h is Planck's constant (6.63×10^{-34} J s), μ_0 is vacuum permittivity (1.26×10^{-6} T^2 J^{-1} m^3), β_e is Bohr magneton (9.27×10^{-24} J T^{-1}) and β_n is nuclear magneton (5.05×10^{-27} J T^{-1}) respectively. The nuclear *g*-values for ^1H: $\boldsymbol{g_H}$ = 5.586. ***g*** and $\boldsymbol{g_n}$ are the electron and nuclear ***g*** (3×3) matrixes (g_n is the nuclear *g*-value, a scalar; **1** is the unit matrix), ρ_k is the electron spin population at atom *k* ($0 \leq \rho_k \leq 1$ and dimensionless), n_k is the *n…k* unit vector expressed in the molecular frame and r_k is the *n…k* distance ($O_2\underline{N}\cdots H$). The *g*-matrix is known from the EPR spectrum. The NO_2 molecular reference frame was defined as the principal axes of the C_{2v} symmetry NO_2 molecule, with which the molecular g and A_N (the ^{14}N hyperfine matrix of NO_2) axes are coincident:

z: parallel to the C_2 axis of NO_2, calculated as the unit vector between N and the midpoint of the O...O atoms;

y: parallel to the O...O direction, calculated as the unit vector;

x: perpendicular to the plane of the NO_2 molecule, calculated as the cross-product of the *y* and *z* unit vectors.

The vectors n are given by $\begin{pmatrix}\cos\alpha\\ \cos\beta\\ \cos\gamma\end{pmatrix}$ where α, β and γ are the angles of the NO_2...H vectors to the molecular NO_2 axes.

For NO_2@MFM-305-CH_3, we initially calculated r, α, β and γ from the SPXRD refined atomic coordinates for the nearby ^1H nuclei (Table S3) using the NO_2 atomic coordinates, since this carries the bulk of the electron spin density. The calculated spectra (Figure S19) in which the closest proton (H1, with an N…H distance of 2.159 Å) gives a large ^1H coupling of up to 4 MHz is not consistent with the experimental spectra. This suggests that the NO_2 molecules are further away from the framework at lower temperature and stabilized by the guest-guest interactions between multiple guest molecules. Therefore, we allowed movement of NO_2 towards the center of the pore to give a longer N…H distance of about 3.4 Å corresponding to the largest experimental ^1H coupling based on a dipole-point model. It should be noted that the N…H(methyl) distance (4.3 Å) was fixed from the ENDOR spectra of MFM-305-CH_3 and MFM-305-CD_3 (Figure S23). We found that the closest protons are H3a and H3b, which gives a N…H3 distance of 3.33(7) Å and 3.64(4) Å, respectively. Orientation-selective ENDOR measurements also show that the largest ^1H coupling to the closest protons are observed along the C_2 axis of NO_2 molecule. Thus, for a better simultaneous agreement to the *x*, *y* and *z* orientation

selective data, we found it necessary to rotate the NO_2 and make the C_2 axis point towards to H3 protons (Table S4, Figure S18).

For NO_2@MFM-305, the calculated spectra do not fit the experimental spectra in the first instance (Table S5, Figure S22). Due to the similarity of experimental spectra of NO_2@MFM-305 and NO_2@MFM-305-CH_3 we started the calculation based on the NO_2 atomic coordinates obtained for MFM-305-CH_3. As a larger ^{1}H coupling is observed in MFM-305, it was reasonable to allow movement of NO_2 towards the framework to achieve better agreement. It should be noted that to simplify the model, only binding site I (which has the highest occupancy) is considered in the calculated spectra (Table S6).

The initial and new positions of NO_2 relative to the framework are shown in Figure S18 (for MFM-305-CH_3) and Figure S21 (for MFM-305).

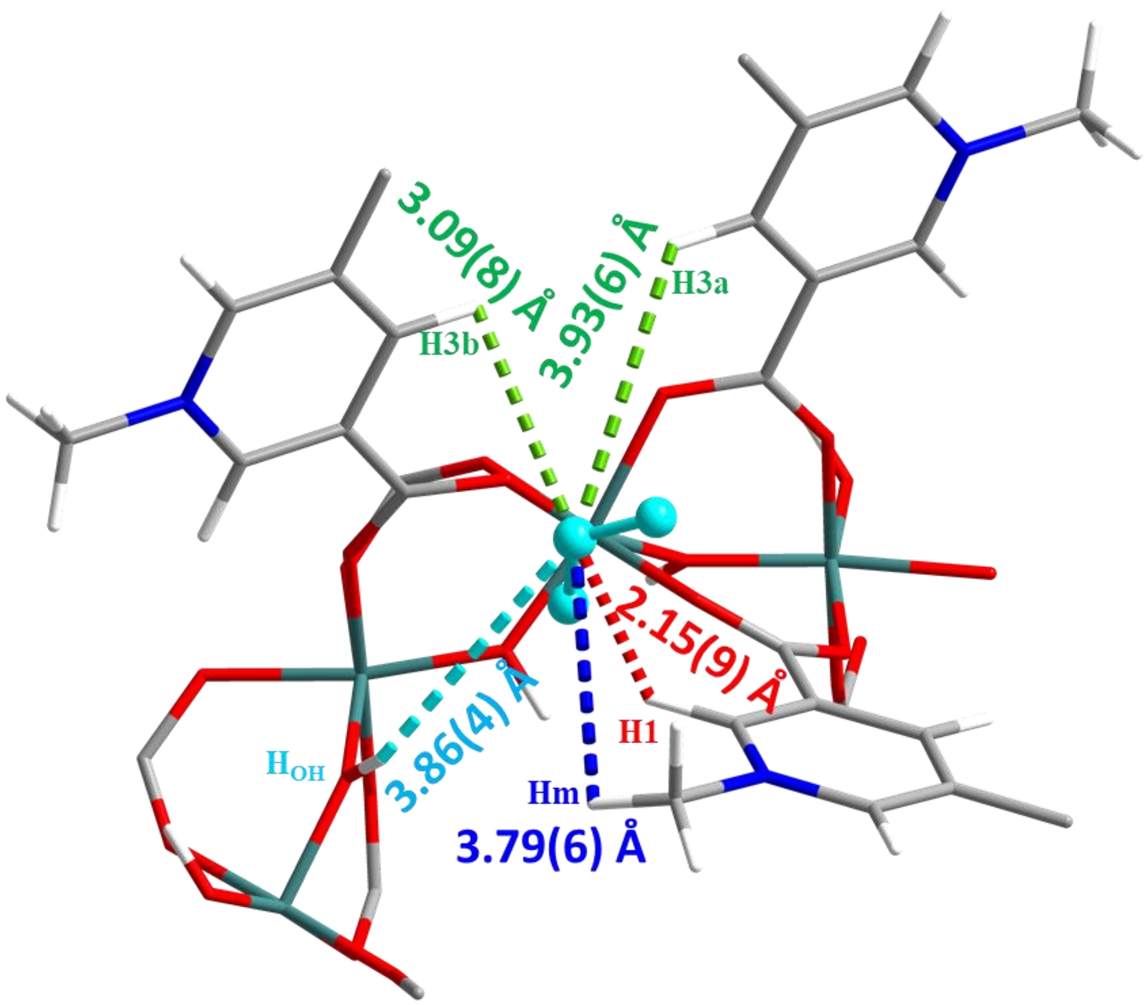

Figure S17. View of O_2<u>N</u>…<u>H</u> interactions between NO_2 and MFM-305-CH_3. The structure was obtained using Rietveld refinement of *in situ* synchrotron X-ray powder diffraction data collected at 298 K (atom colour scheme as in Figure 1; cyan: NO_2 molecule).

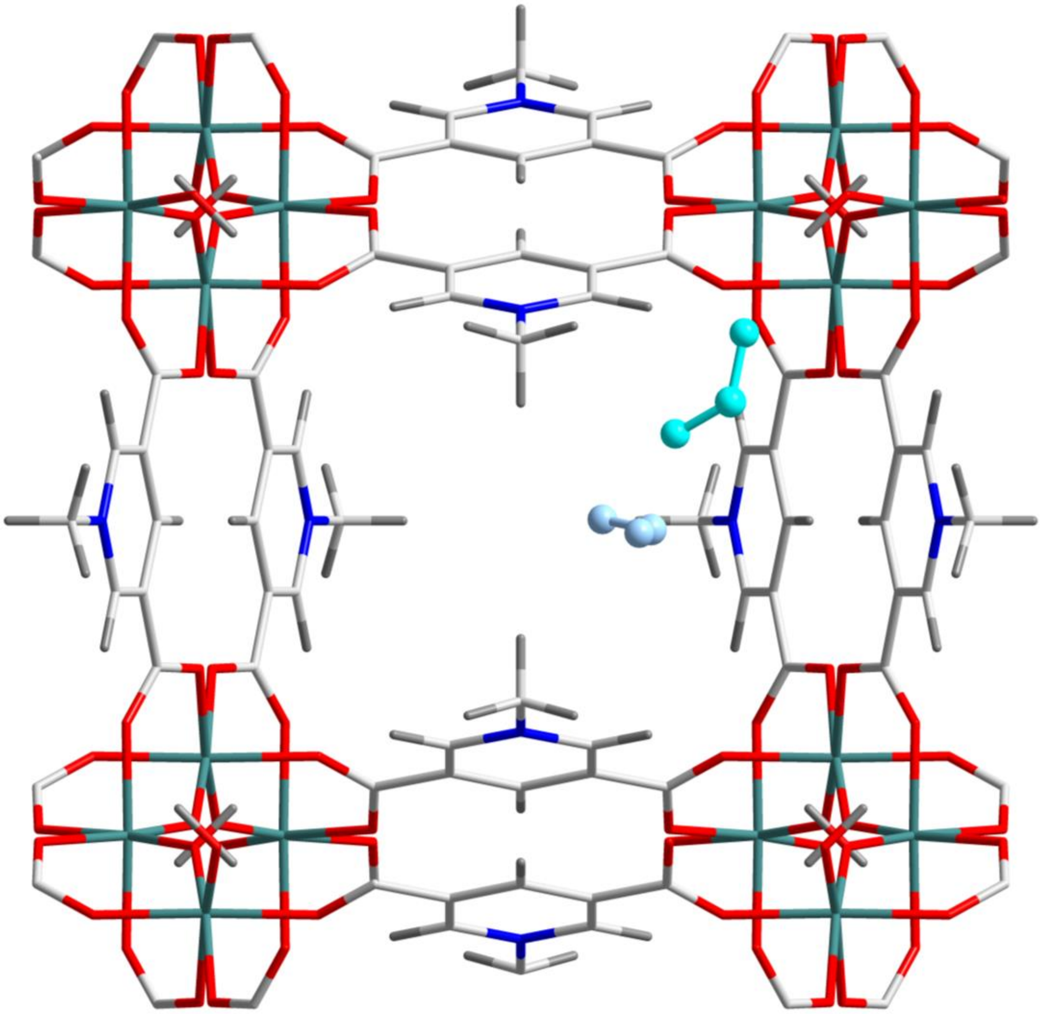

Figure S18. View of initial (cyan) and final (light blue) position of NO_2 in NO_2@MFM-305-CH_3. Initial position is obtained from Rietveld refinements of *in situ* synchrotron X-ray powder diffraction data; new position is obtained from movement and rotation of NO_2 molecule according to ENDOR spectra (Figure 5c) (atom colour scheme as in Figure 1).

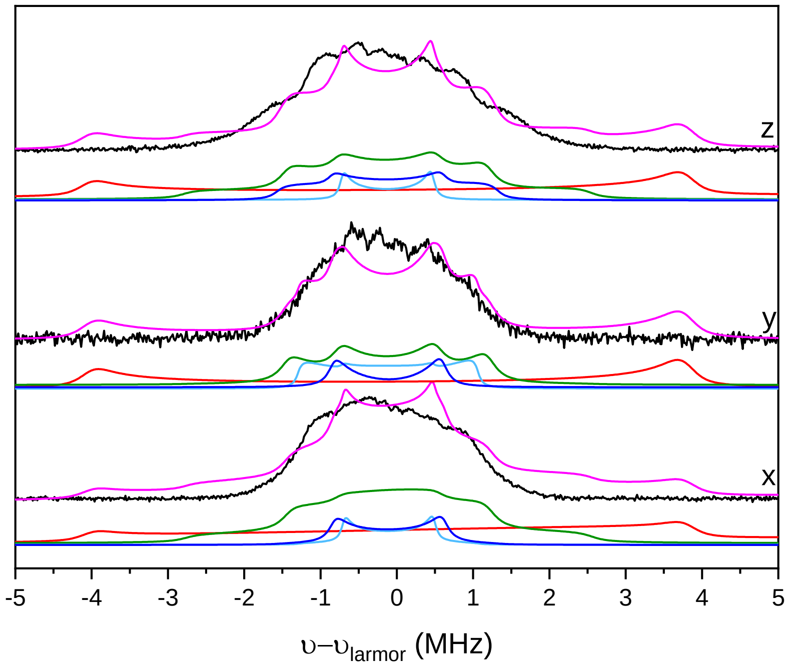

Figure S19. X-band Davies ENDOR spectrum of NO_2@ MFM-305-CH_3 (black) and calculated spectra based on the SPXRD refined structure (magenta: sum; red: H1; green: H3; blue: H_{methyl}; cyan: H_{OH}).

Table S3 Structural details for NO_2@MFM-305-CH_3 derived from SPXRD used for calculation of ENDOR spectra in Figure S19 (atom labelling in Figure S17).

	SPXRD refined N…H distance / Å	α / degrees	β / degrees	γ / degrees
H1	2.159	35.8	74.6	58.6
H3a	3.936	97.5	113.8	154.9
H3b	3.098	138.5	100.0	129.8
H_{OH}	3.864	66.9	25.0	99.2
H_{methyl}	3.796	69.7	88.6	20.3

Table S4. Structural details for NO_2@MFM-305-CH_3 derived from SPXRD after allowing movement of NO_2 and used for calculation of ENDOR spectra in Figure 5c (atom labelling in Figure 5c).

	ENDOR N···H distance / Å	α/ degrees	β/ degrees	γ/ degrees
H1	4.027	77.5	102.0	17.5
H3a	3.337	159.7	70.8	83.6
H3b	3.644	153.0	114.5	79.4
H_{methyl}	4.216	40.6	115.7	61.0

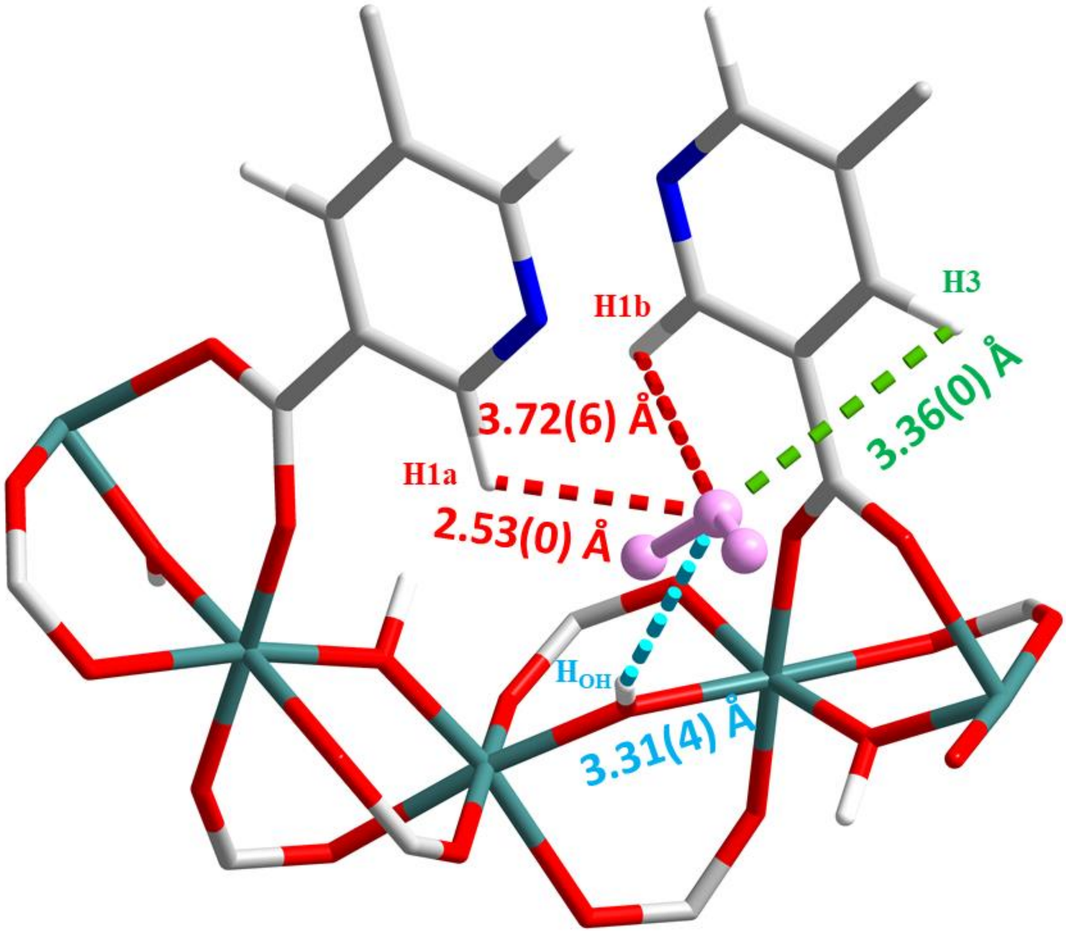

Figure S20. View of O_2<u>N</u>…<u>H</u> interaction between NO_2 and framework in MFM-305. The structure was obtained by Rietveld refinement of *in situ* synchrotron X-ray powder diffraction data collected at 298 K (Atom colour scheme as in Figure 2.1).

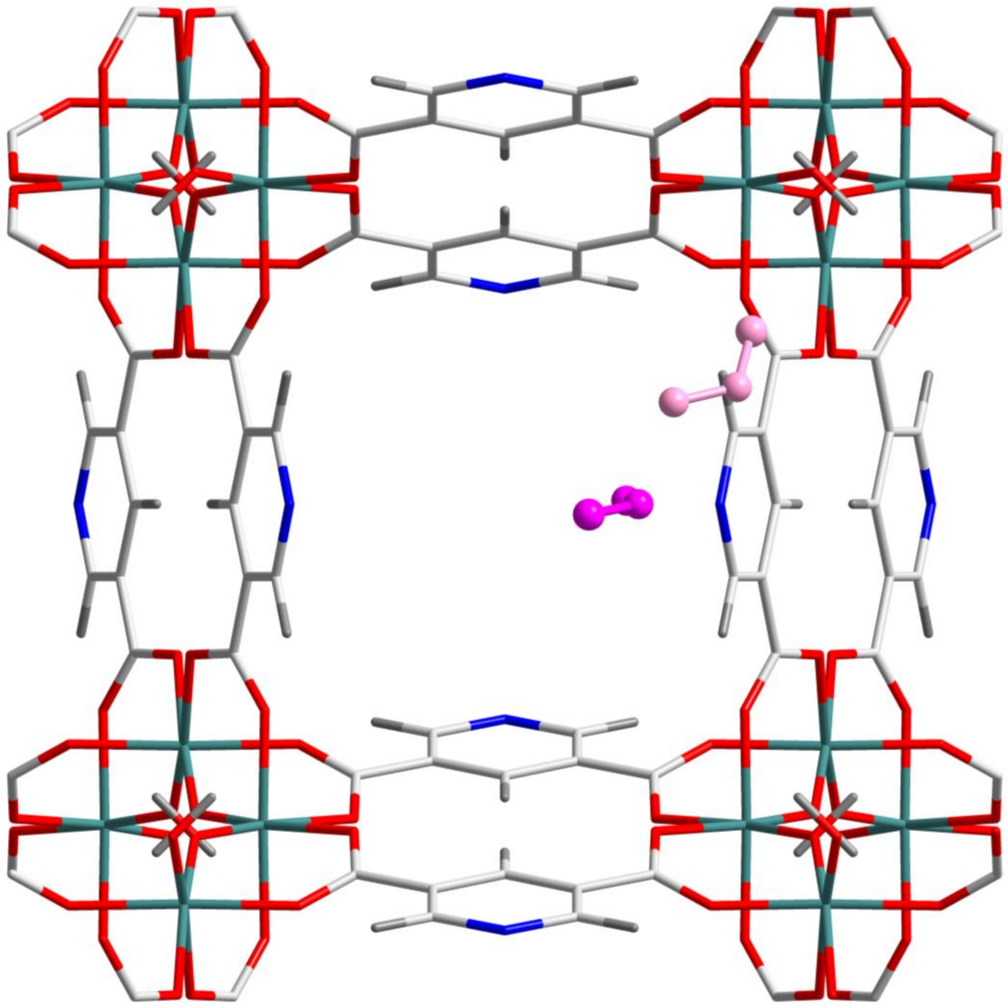

Figure S21. Initial (light pink) and final (magenta) position of NO_2 in NO_2@MFM-305. Initial position is obtained from Rietveld refinements of *in situ* synchrotron X-ray powder diffraction data; new position is obtained from movement and rotation of NO_2 molecule according to ENDOR spectra (Atom colour scheme as in Figure 1).

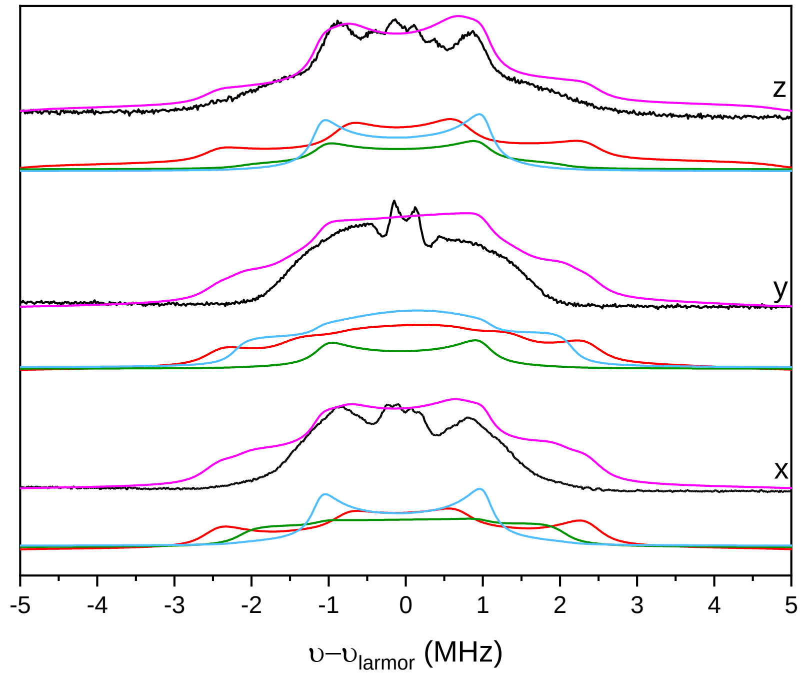

Figure S22. X-band Davies ENDOR spectrum of NO_2@ MFM-305 (black) and calculated spectra based on the SPXRD refined structure (magenta: sum; red: H1; green: H3; cyan: H_{OH}).

Table S5. Structural details for NO_2@MFM-305 derived from SPXRD used for calculation of ENDOR spectra in Figure S22 (atom labelling in Figure S20).

	SPXRD refined N…H distance / Å	α / degrees	β / degrees	γ / degrees
H1a	2.530	69.9	123.1	139.9
H1b	3.726	123.0	146.3	95.4
H3	3.360	157.9	74.8	74.2
HOH	3.314	68.4	146.6	65.6

Table S6. Structural details for NO_2@MFM-305 derived from SPXRD after allowing movement of NO_2 and used for calculation of ENDOR spectra in Figure 5d (atom labelling in Figure 5d).

	ENDOR N···H distance / Å	α/ degrees	β/ degrees	γ/ degrees
H1	3.878	77.2	77.6	162
H3a	3.333	160.3	109.3	93.8
H3b	3.621	154.0	65.5	98.2

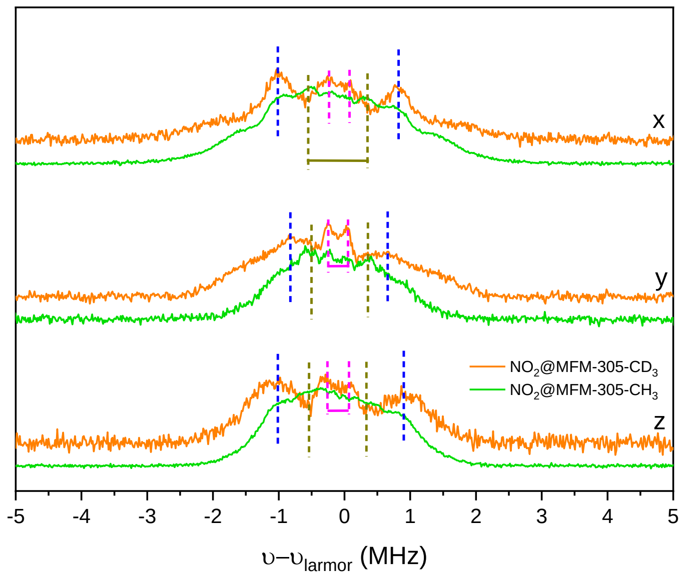

Figure S23. Comparison of X-band Davies ENDOR spectra of NO_2@MFM-305-CH_3 and NO_2@MFM-305-CD_3 (blue and magenta dotted line: positions of ^1H hyperfine peaks appeared in both NO_2@MFM-305-CH_3 and NO_2@MFM-305-CD_3 spectra; olive dotted line: positions of ^1H hyperfine peaks only appeared in NO_2@MFM-305-CH_3 spectra, indicating these peaks are contributed from -CH_3 protons).

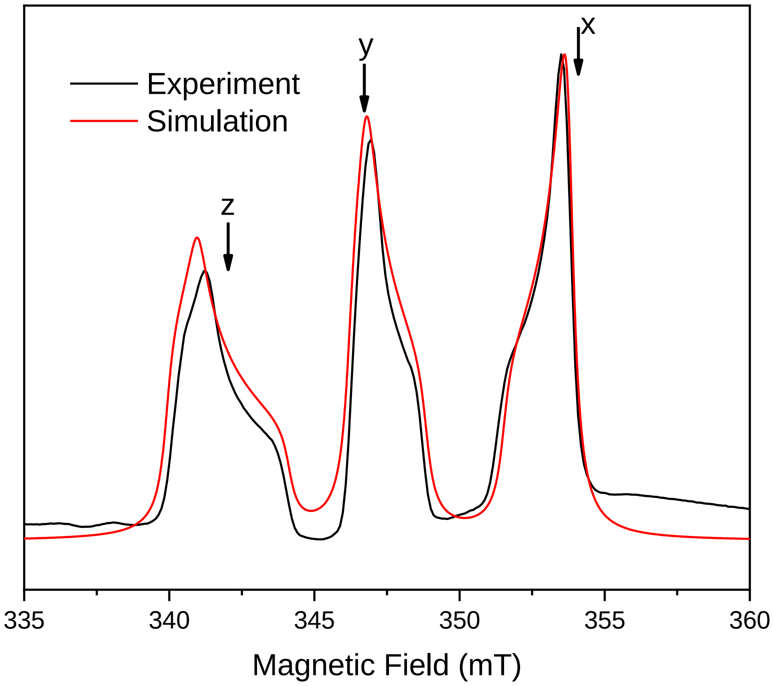

Figure S24. X-band (9.73 GHz) EDFS spectrum of NO_2-loaded MFM-305-CH_3 at 10 K (simulation parameters in Table S7).

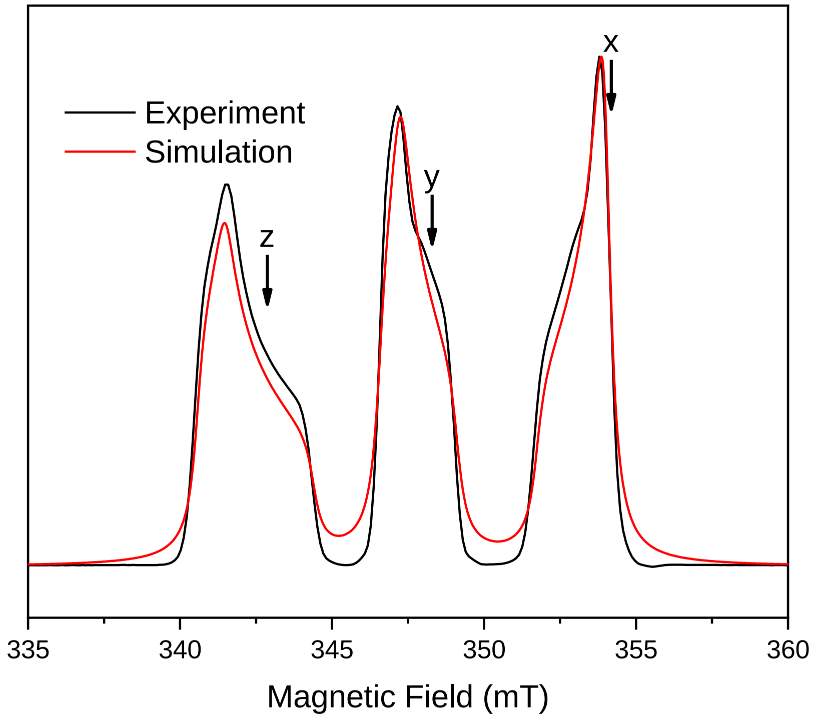

Figure S25. X-band (9.73 GHz) EDFS spectrum of NO_2-loaded MFM-305 at 10 K (simulation parameters in Table S7).

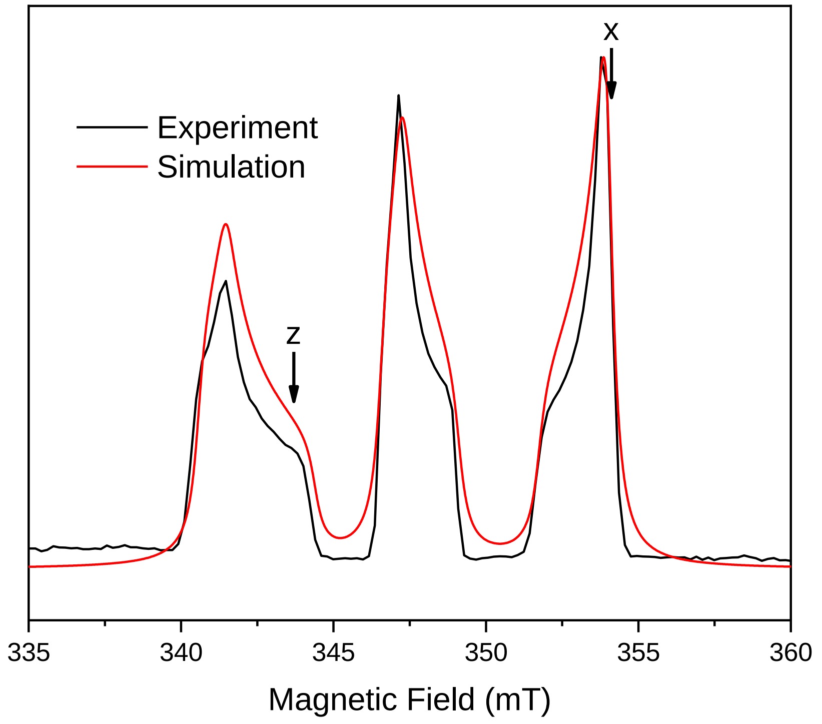

Figure S26. X-band (9.73 GHz) EDFS spectrum of NO_2-loaded MFM-305-CD_3 at 10 K (simulation parameters in Table S7).

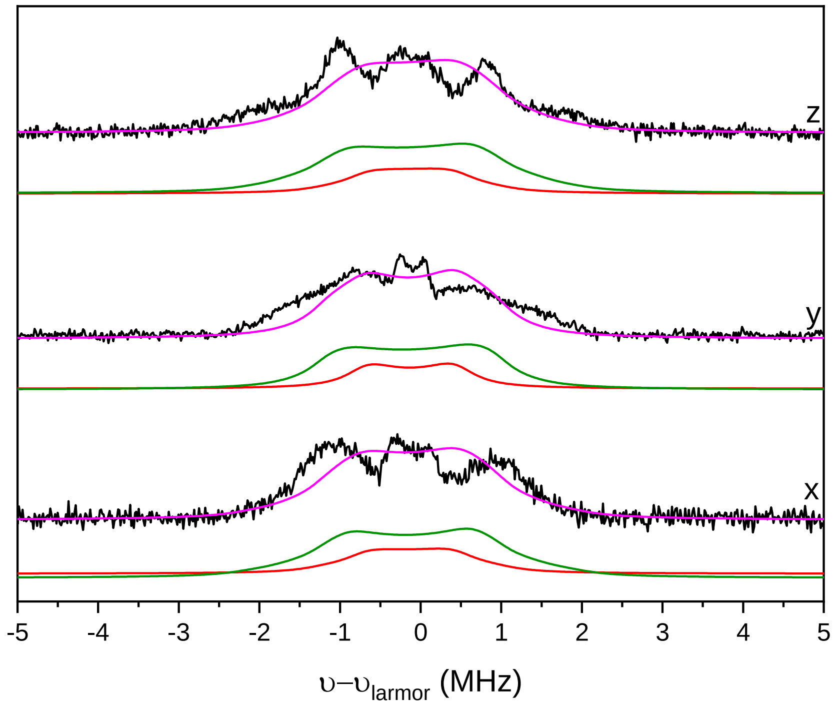

Figure S27. X-band Davies ENDOR spectrum of NO_2@MFM-305-CD_3 (black) at 10 K and simulated spectra with identical parameters of NO_2@MFM-305-CH_3. (Magenta: sum; red: H1; green: H3; definition of H1/H3 are identical with MFM-305-CH_3, shown in Figure 5c).

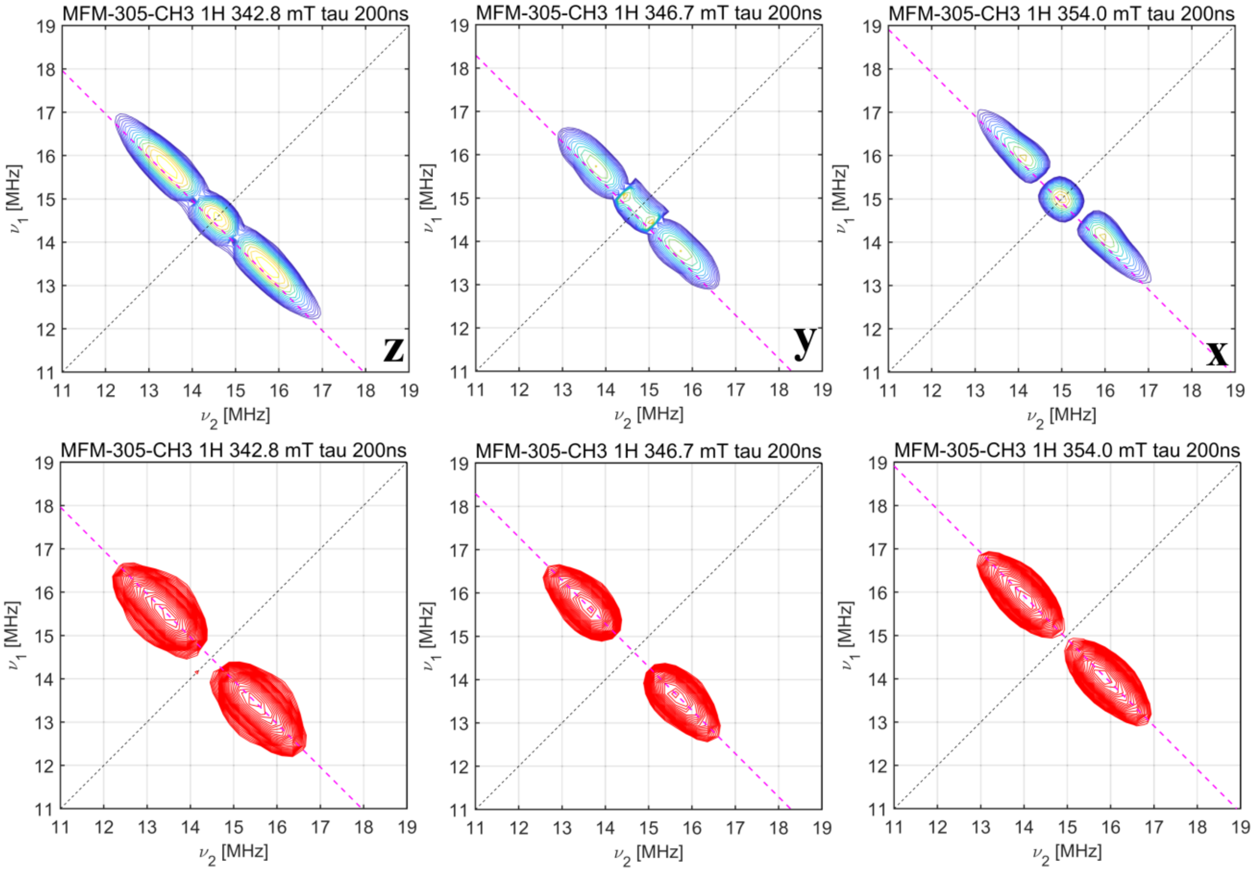

Figure S28. X-band (9.73) ^{1}H HYSCORE spectra (recorded at 10 K) of NO_2@MFM-305-CH_3 measured at static field (z: 342.8, y: 346.7 and x: 354.0 mT) (upper); simulated spectra (lower) from identical parameters with ENDOR spectra (from Table S4). The anti-diagonal dashed lines (magenta) cross the diagonal at the Larmor frequencies for [1]H.

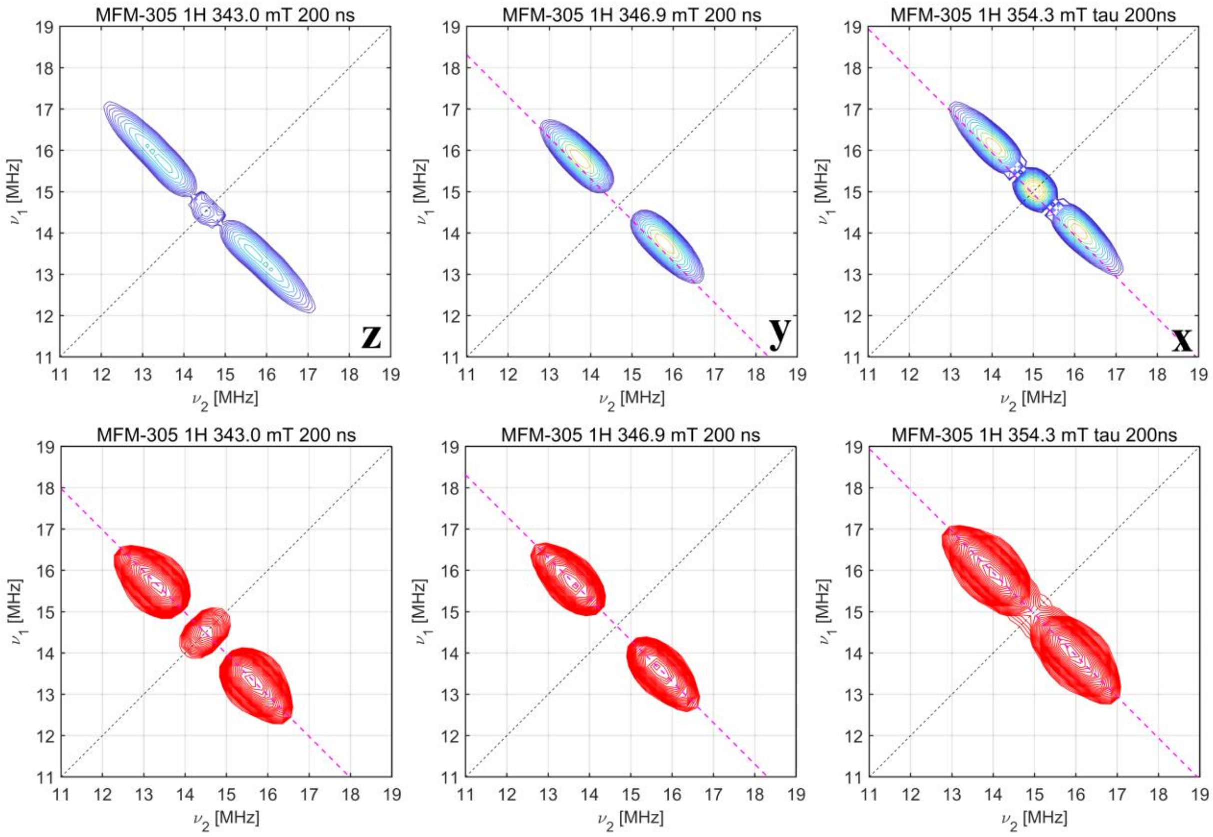

Figure S29. X-band (9.73) ^{1}H HYSCORE spectra (recorded at 10 K) of NO_2@MFM-305 measured at static field (z: 343.0, y: 346.9 and x: 354.3 mT) (upper); simulated spectra (lower) from identical parameters with ENDOR spectra (from Table S6). The anti-diagonal dashed lines (magenta) cross the diagonal at the Larmor frequencies for ^{1}H.

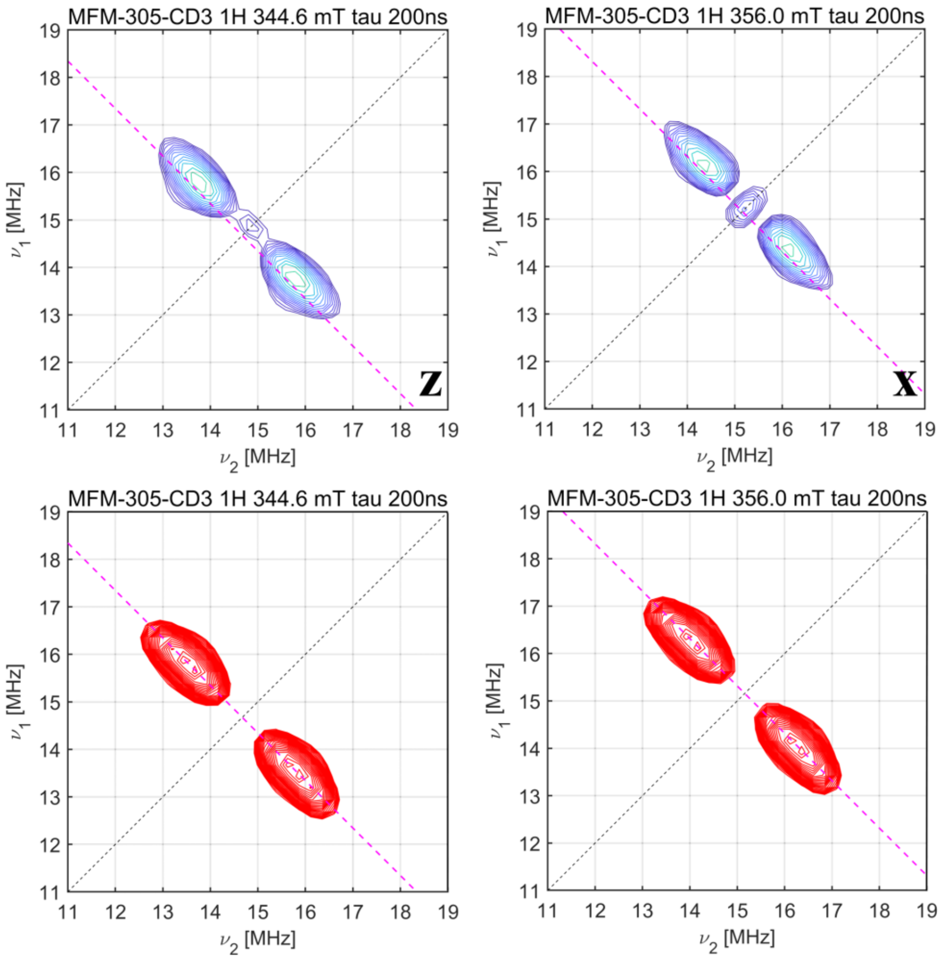

Figure S30. X-band (9.73) ^{1}H HYSCORE spectra (recorded at 10 K) of NO_2@MFM-305-CD_3 measured at static field (z: 344.6 and x: 356.0 mT) (upper); simulated spectra with identical parameters of NO_2@MFM-305-CH_3 (Table S4). The anti-diagonal dashed lines (magenta) cross the diagonal at the Larmor frequencies for [1]H.

Table S7. Simulation parameters of pulse ^{14}N HYSCORE spectra at X-band of NO_2@MFM-305.

NO_2@MFM-305							
Nuclei	Electron Spin	Nuclei Spin	*g*-matrix	Euler angle /degree	T/MHz	A_{iso} /MHz	$\underline{N}O_2$-$\underline{N}_2O_4$ Distance/ Å
^{14}N	1/2	1	[2.006 1.9913 2.0022]	[0 0 0]	1.1	0.2	1.85

* It was not necessary to introduce nuclear quadrupole effects in the simulation.

The spectra were modelled considering contributions from the electron-nuclear (^1H) dipolar and isotropic hyperfine interactions: $A = A_{dip} + A_{iso}$, A_{dip} = [-T; -T; +2T]. Here, $T = \mu_0 g_e g_n \mu_e \mu_n / 4\pi r^3$, where μ_0 is the vacuum permeability, μ_n is the nuclear magneton, g_n is the nuclear *g*-factor and *r* is the $\underline{N}O_2$-$\underline{N}_2O_4$ distance.

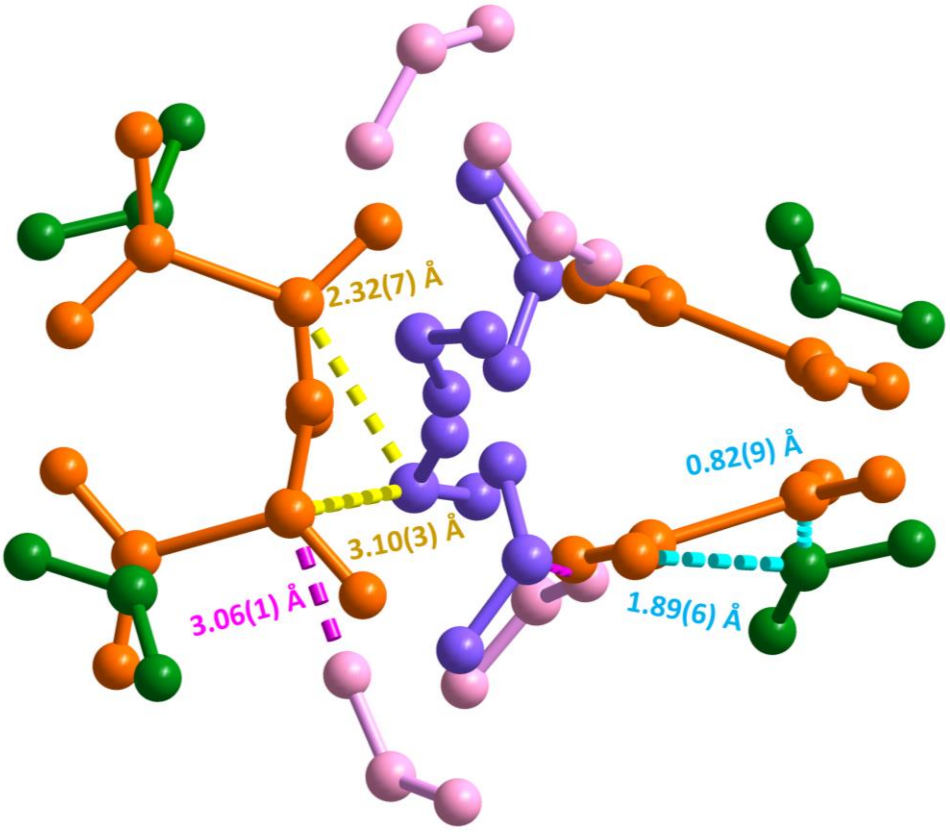

Figure S31. View of $O_2\underline{N}\ldots\underline{N}_2O_4$ interaction between NO_2 and N_2O_4 in MFM-305. The structure was obtained by Rietveld refinement of *in situ* synchrotron X-ray powder diffraction data collected at 298 K (orange: N_2O_4; pink: NO_2 at site I; green: NO_2 at site II; purple: NO_2 at site III).

References

[1] L. Li, I. da Silva, D. I. Kolokolov, X. Han, J. Li, G. Smith, Y. Cheng, L. L. Daemen, C. G. Morris, H. G. W. Godfrey, N. M. Jacques, X. Zhang, P. Manuel, M. D. Frogley, C. A. Murray, A. J. Ramirez-Cuesta, G. Cinque, C. C. Tang, A. G. Stepanov, S. Yang, M. Schröder, *Chem. Sci*. **2019**, 10, 1472–1482.

[2] X. Han, Y. Hong, Y. Ma, W. Lu, J. Li, L. Lin, A. M. Sheveleva, F. Tuna, E. J. L. McInnes, C. Dejoie, J. Sun, S. Yang, M. Schröder, *J. Am. Chem. Soc*. **2020**, 142, 15235–15239.

[3] A. A. Coelho, *J. Appl. Cryst*. **2018**, 51, 210-218.

[4] J. R. Carvajal, *J. Appl. Cryst*. **1999**, 32, 281-289.

[5] J. Hutter, M. Iannuzzi, F. Schiffmann, J. VandeVondele, WIREs Comput. *Mol. Sci*. **2014**, 4, 15–25.

[6] G. Lippert, J. Hutter, M. Parrinello, *Mol. Phys*. **1997**, 92, 477–487.

[7] J. VandeVondele, M. Krack, F. Mohamed, M. Parrinello, T. Chassaing, J. Hutter, *Comput. Phys. Commun*. **2005**, 167, 103–128.

[8] J. VandeVondele, J. Hutter, *J. Chem. Phys*. **2007**, 127, 114105.

[9] S. Goedecker, M. Teter, J. Hutter, *Phys. Rev. B* **1996**, 54, 1703–1710.

[10] J. P. Perdew, K. Burke, M. Ernzerhof, *Phys. Rev. Lett*. **1996**, 77, 3865–3868.

[11] S. Grimme, J. Antony, S. Ehrlich, H. Krieg, *J. Chem. Phys*. **2010**, 132, 154104.

[12] Y. Q. Cheng, L. L. Daemen, A. I. Kolesnikov, A. J. Ramirez-Cuesta, J. *Chem. Theory Comput*. **2019**, 15, 1974–1982.

[13] B. M. Fung, A. K. Khitrin, K. Ermolaev, *J. Magn. Reson*. **2000**, 142, 97–101.

[14] G. Teymoori, B. Pahari, B. Stevensson, M. Edén, *Chem. Phys. Lett*. **2012**, 547, 103–109.

[15] G. Teymoori, B. Pahari, M. Edén, *J. Magn. Reson*. **2015**, 261, 205–220.

[16] A. Brinkmann, A. P. M. Kentgens, *J. Am. Chem. Soc*. **2006**, 128, 14758–14759.

[17] D. Iuga, E. K. Corlett, S. P. Brown, *Magn. Reson. Chem*. **2021**, 59, 1089–1100.

[18] S.-J. Huang, S.-B. Liu, J. C. C. Chan, *Solid State Nucl. Magn. Reson*. **2009**, 36, 110–117.

[19] Z. Gan, J. P. Amoureux, J. Trébosc, *Chem. Phys. Lett*. **2007**, 435, 163–169.

[20] E. Kupce, R. Freeman, *J. Magn. Reson. A* **1995**, 115, 273-276.

[21] K. K. Dey, S. Prasad, J. T. Ash, M. Deschamps, P. J. Grandinetti, *J. Magn. Reson*. **2007**, 185, 326–330.

[22] S. Stoll, A. Schweiger, *J. Magn. Reson*. **2006**, 178, 42–55.
